# *N*‐acetylglucosamine sensing in the filamentous soil fungus *Trichoderma reesei*

**DOI:** 10.1111/febs.70015

**Published:** 2025-02-15

**Authors:** Sadia Fida Ullah, Mislav Oreb, Eckhard Boles, Vaibhav Srivastava, Verena Seidl‐Seiboth, Bernhard Seiboth, Lisa Kappel

**Affiliations:** ^1^ Division of Glycoscience, Department of Chemistry, KTH Royal Institute of Technology AlbaNova University Centre Stockholm Sweden; ^2^ Faculty of Biological Sciences, Institute of Molecular Biosciences Goethe University Frankfurt Germany; ^3^ Research Division Biochemical Technology, Institute of Chemical, Environmental and Bioscience Engineering TU Wien Vienna Austria; ^4^ Present address: Institute of Science and Technology, IST – Austria Klosterneuburg Austria; ^5^ Present address: Division of Glycoscience, Department of Chemistry KTH Royal Institute of Technology, AlbaNova University Centre Stockholm Sweden

**Keywords:** major facilitator superfamily, *N*‐acetylglucosamine, secondary metabolism, signaling, soil fungi, *Trichoderma* spp.

## Abstract

*N*‐acetylglucosamine (GlcNAc) is involved in diverse signaling pathways in dimorphic yeasts and bacteria and is related to morphogenetic switching, mating, stress, virulence, and cell death. Recently, GlcNAc has been shown to promote plant growth by shaping the bacterial soil community. However, the role of GlcNAc sensing in filamentous soil fungi has not been investigated. By using *Trichoderma reesei* as a model organism, we show here that GlcNAc impacts the expression of around 2100 genes. Carbohydrate metabolism, amino acid metabolism, and secondary metabolism were the three most strongly affected classes of eukaryotic orthologous groups (KOG classes). Two key regulators of GlcNAc catabolism, the NDT80 domain‐containing transcriptional regulator RON1, and a GlcNAc sensor, NGS1, are needed for differential regulation of two‐thirds of these genes. *In silico* structural modeling of NGS1 identified a domain with homology to the GCN5‐related histone acetyltransferase from *Candida albicans*, which serves as a GlcNAc catabolism regulator and GlcNAc sensor. Finally, we characterized the third regulator of GlcNAc sensing in *T. reesei*, which is the highly specific GlcNAc transporter *N*‐acetylglucosamine transporter (NGT1). Using a deletion mutant of *ngt1*, we demonstrate that GlcNAc has to enter the cell to activate the GlcNAc catabolic gene expression. Interestingly, in contrast to dimorphic yeasts, the pathways for defense and pathogenicity seem to be induced in *T. reesei* by external GlcNAc. Given the ancestral role of *Trichoderma* spp. in the fungal kingdom and the highly conserved GlcNAc catabolism cluster that includes their regulators in many species of fungi, we propose a regulatory network for GlcNAc sensing in soil fungi.

## Graphical abstract

*N‐*acetylglucosamine (GlcNAc) is involved in diverse signaling pathways in dimorphic yeasts and bacteria, including virulence and cell death. Here, we investigated GlcNAc sensing in *Trichoderma reesei*. GlcNAc signaling influences around 2100 genes in this filamentous soil fungus. RON1 and NGS1 regulate two‐thirds of these genes and promote GlcNAc catabolism, while CRE1 acts as a repressor. The major facilitator superfamily transporter NGT1 imports GlcNAc and may distinguish external GlcNAc from internal sources. Internalized GlcNAc activates catabolic gene expression, whereas external GlcNAc may regulate pathogenicity and defense‐related genes.

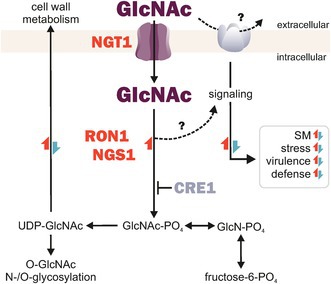

**Abbreviations**
CCR: carbon catabolite repression
CRE1: catabolite responsive element
DAC1: *N*‐acetylglucosamine‐6‐phosphate deacetylase
DAM1: Glucosamine‐6‐phosphate deaminase
DEG: differentially expressed gene
FET: fisher's exact test
GIG1: GlcNAc‐induced gene
GlcNAc: *N*‐acetylglucosamine
GNAT: Gcn5‐related *N*‐acetyltransferases
HAT: histone acetyl transferase
HXK3: *N*‐acetylglucosamine hexokinase
JGI: Joint Genome Institute
KOG: classes of eukaryotic orthologous groups
MFS: major facilitator superfamily
NAG: *N*‐acetyl glucosaminidase
NGS1: *N*‐acetylglucosamine sensor
NGT1: *N*‐acetylglucosamine transporter
*P*adj: adjusted *P*‐value
PCR: polymerase chain reaction
PDA: potato dextrose agar
PDB: protein data bank
RON1: Regulator of *N*‐acetylglucosamine catabolism
VMD: visual molecular dynamics
WT: wild‐type

## Introduction

*N*‐acetylglucosamine (GlcNAc) is an omnipresent and abundantly available amino sugar that serves as a building block for various structural cell components, such as chitin, peptidoglycan, and glucosaminoglycans (hyaluronan, heparin, and keratan), and is an integral part of the sugar coat of glycosylated proteins (reviewed in [1, 2, 3]). Due to its prevalence, microorganisms, especially pathogens, can utilize GlcNAc as carbon‐ and nitrogen source [1, 4]. Notably, in dimorphic yeasts, GlcNAc also activates gene expression associated with virulence, oxidative stress and defense against antifungal agents, and even induces cell death [5, 6, 7, 8, 9]. Recent findings have shown that GlcNAc can also promote plant growth by shaping the bacterial soil community associated with the plant roots [10]. Soil fungi are equally important players in plant health and growth [11]. Previously, we showed that the GlcNAc catabolism cluster is conserved in soil‐dwelling filamentous fungi and that most of the investigated species use GlcNAc efficiently as an alternative carbon source [12, 13]. However, the role of GlcNAc beyond catabolism in filamentous fungi remained unexplored.

*Trichoderma* spp. are filamentous soil fungi with important roles in agriculture as biocontrol agents and in industry as efficient producers of secreted carbohydrate‐active enzymes [14]. In *Trichoderma* spp. GlcNAc catabolism is coordinated by a Ndt80‐like transcription factor RON1 which activates expression of three structural genes organized in a gene cluster on chromosome 6 in *T. reesei* [13, 15, 16]. The cluster organization is conserved within several other previously investigated fungal genomes [8, 13, 17]. The gene products, a GlcNAc hexokinase (HXK3), a GlcNAc deacetylase (DAC1), and a GlcN deaminase (DAM1), convert GlcNAc in three consecutive steps to fructose‐6‐phosphate to enter glycolysis [13]. In *Candida albicans*, the gene CaNgs1, encoding a GCN5‐related histone acetyltransferase, serves as second regulator of GlcNAc catabolism [18]. An orthologous gene, jgi|Trire2|79 669, with an additional family 3 glycoside hydrolase (GH3) domain (*nag3*) was identified, previously. It is present in the GlcNAc utilization gene cluster (hereafter referred to as ‘GlcNAc gene cluster’) and the expression of *nag3* is dependent on RON1, but it has not been further characterized [13]. In *T. reesei* and *C. albicans*, deletion of any of these clustered genes results in a complete loss of the ability to use GlcNAc for catabolism [13, 18, 19]. In addition, depletion of these genes or their transcripts also impacted hyphal morphogenesis in *C. albicans* and other dimorphic yeasts [8, 20, 21]. This was confirmed also for the transcription factor homolog, MoNdt80, in the plant pathogen *Magnaporthe oryzae*, which is also important for pathogenicity [22]. NGT1 is the GlcNAc sugar porter in dimorphic yeasts [23], and a homolog in filamentous fungi has been identified [13]. NGT1 belongs to the major facilitator superfamily (MFS), which represents an ancient group of secondary carriers and members of MFS are found throughout all kingdoms of life. NGT1 is needed to induce the dimorphic switch in *Candida* spp. [23, 24]. In plant beneficial filamentous soil fungi, such as *Trichoderma*, the contribution of GlcNAc utilization cluster genes and the transporter NGT1 to GlcNAc sensing has not been characterized.

This work delves into GlcNAc sensing in filamentous soil fungi, employing the filamentous ascomycete *T. reesei* as a model organism. We reveal that two key regulators (RON1 and NGS1) and the GlcNAc transporter NGT1 are crucial checkpoints for specific signaling cascades in *T. reesei*. We determine that NGT1 is the only specific GlcNAc transporter in *T. reesei*. By using a deletion mutant of *ngt1* we provide evidence that in contrast to the data from dimorphic yeasts, distinct GlcNAc signaling cascades may be activated differentially by intra‐ or extracellular GlcNAc. Given the conservation of the GlcNAc utilization genes in Ascomycota [13], we propose that the regulators present in the cluster and the GlcNAc specific transporter play a major role in GlcNAc sensing in these filamentous (soil) fungi, which might impact their interaction with the environment.

## Results

### Transcriptional level of regulation of GlcNAc sensing

We previously showed that GlcNAc can serve as an efficient carbon source and that its catabolism is facilitated by joint expression of a conserved gene cluster in filamentous fungi [12, 13]. To get an insight beyond its role in GlcNAc catabolism in filamentous fungi, we investigated the impact of the presence of GlcNAc on the gene expression profile of *T. reesei*.

### *N*‐acetylglucosamine affects expression of genes from primary and secondary metabolism

**Fig. 1:**
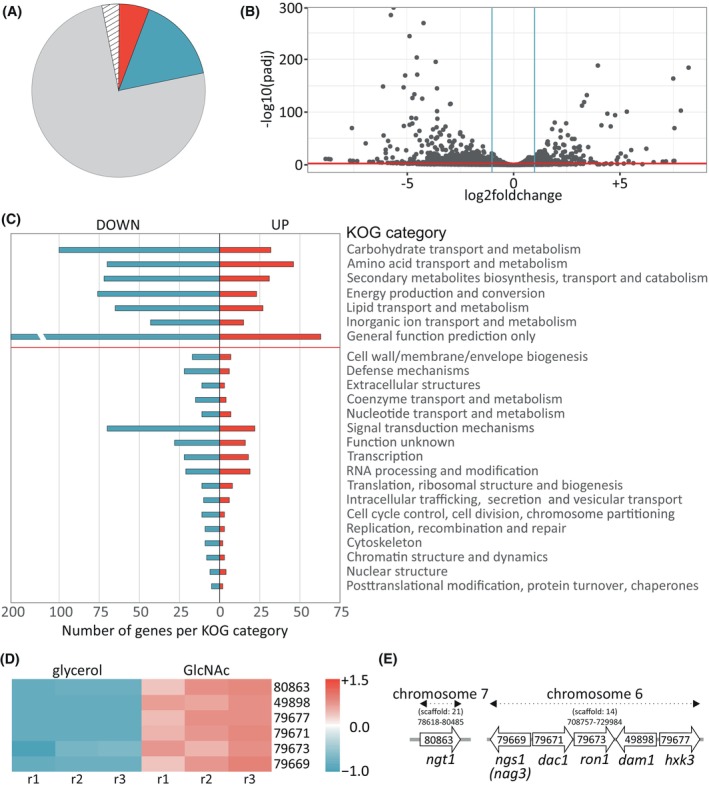
*N*‐acetylglucosamine (GlcNAc) affects expression of genes from primary and secondary metabolism. (A) Pie chart depicting gene expression changes in *T. reesei* in the presence of 1% GlcNAc compared with 1% glycerol. The relative amounts of upregulated (red) and downregulated (blue) genes, unaffected genes (gray) and genes not expressed (hatched) in the RNA‐seq analysis of three biological replicates per condition (*n* = 3) in response to GlcNAc are shown. (B) Volcano plot of significantly differentially regulated genes from A, *P*adj <0.05 (red bar), log_2_‐fold change >|1.0| (blue bars) *n* = 3. (C) Eukaryotic orthologous groups (KOG) enrichment analysis of 2102 differentially expressed genes in the presence of GlcNAc compared with glycerol. The number of up‐ and downregulated genes per KOG class is indicated at the x‐axis. Upregulated genes are presented in red, downregulated genes in blue. A red line separates the significantly enriched KOG categories from the others *n* = 3. (D) Heat map of normalized counts for growth on glycerol versus GlcNAc of all GlcNAc cluster genes and the GlcNAc transporter. The JGI‐identifiers (joint genome institute database) are given on the right. r1–r3 represent biological replicates. (E) Schematic representation of the location and relative position of the GlcNAc cluster genes and the GlcNAc transporter. Chromosome and scaffold position are indicated, JGI‐identifiers and proposed names are shown. Distance and gene size are not to scale.

First, the gene expression in the presence of GlcNAc compared with glycerol, a noninducing carbon source for GlcNAc expression, was investigated. Glycerol is another omnipresent carbon source [25] that, in contrast to glucose, does not act as carbon catabolite repressor. Remarkably, growth on GlcNAc induced significant differential regulation of 2102 genes (*P*adj <0.05, log_2_‐fold change >|1.0|, Fig. 1A,B; Tables S1 and S10) out of the 9143 annotated genes in the *T. reesei* genome [26]. In the presence of GlcNAc, 561 genes exhibited a significant upregulation, while 1541 genes were downregulated. Additionally, we identified 295 genes that were not expressed in either of the tested conditions.

KOG annotation [27] revealed that six KOG categories are strongly enriched in the presence of GlcNAc (Fig. 1C; Table S2). Furthermore, a seventh KOG category for genes that cannot be clearly grouped, that is, ‘General function prediction only’, was also enriched (264 DEGs, FET: 5.2E‐03). Five categories are related to primary metabolism, including 132 differentially expressed genes (DEGs) from ‘carbohydrate transport and metabolism’ (fishers exact test (FET): 2.27E‐12), 116 DEGs from ‘amino acid transport and metabolism’ (FET: 2.39E‐12), 99 DEGs from ‘energy production and conversion’ (FET: 6.45E‐05), 92 DEGs from ‘lipid transport and metabolism’ (FET: 1.57E‐04), and 58 DEGs from ‘inorganic ion transport and metabolism’ (FET: 5.35E‐04). Within the group ‘carbohydrate transport and metabolism’, the genes from the GlcNAc gene cluster were most strongly upregulated (Fig. 1D,E, Tables S1 and S10). These results confirm findings from our previous work and indicate that these genes are not only derepressed in the absence of glucose [13], but that they are actively expressed in the presence of GlcNAc compared with the noninducing carbon source glycerol. Interestingly, one additional category related to ‘secondary metabolite biosynthesis, transport and catabolism’ (FET: 1.27E‐10) was strongly enriched with 103 DEGs in the presence of GlcNAc. Furthermore, genes from another 17 KOG categories showed differential expression, but these were not enriched within their respective class (Fig. 1C; Table S2).

### A gene product highly homologous to GCN5‐related histone acetyltransferases is encoded in the GlcNAc gene cluster

**Fig. 2:**
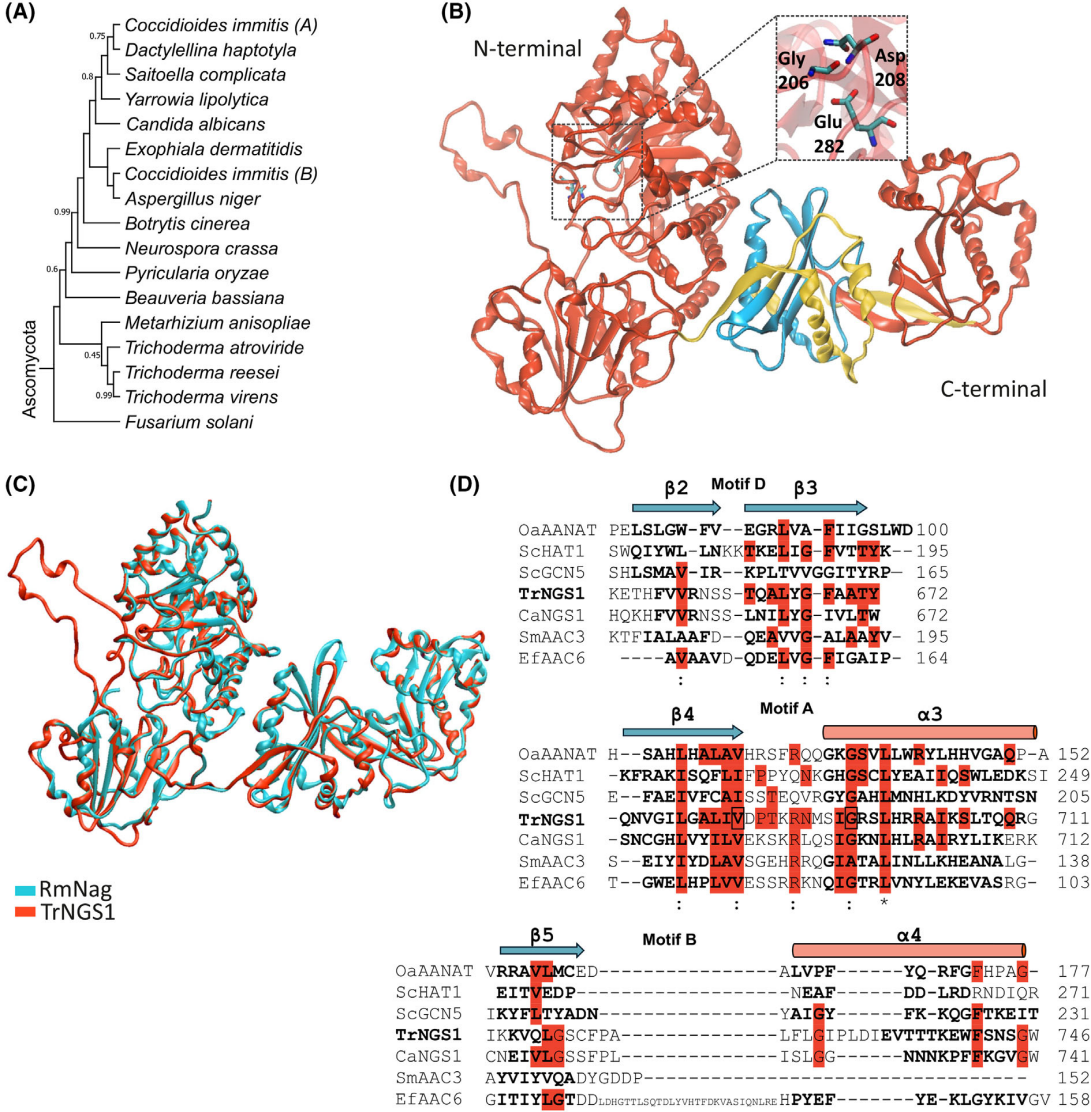
NGS1 is highly homologous to GCN5‐related histone acetyl transferases. (A) Phylogenetic analysis of putative NGS1 homologs in Ascomycota. Mega11 with neighbor joining was used and stability of clades using 1000 bootstraps iterations is indicated if probability values were below 1. (B) Homology model of TrNGS1. The *N*‐ and *C*‐terminal region of β‐*N*‐acetylglucosaminidase is depicted in red with a zoom in view of the variant catalytic residues Gly206, Asp208 and Glu282 presented as color sticks. These residues are highlighted in blue in the alignment file (Fig. S4). The Gcn5‐related *N*‐acetyltransferase (GNAT) region is highlighted in yellow and the histone acetyl transferase (HAT) region in cyan. (C) Monomeric overlapping structures of TrNGS1 (in red) and RmNag (in blue). Graphics in B and C were created using VMD [74]. The UniProt accession numbers are G0RNA9 for TrNGS1 and V9M3A9 for RmNAG. (D) Sequence alignment of structurally similar GCN5‐related *N*‐acetyltransferases with UniProt accession numbers Q29495 for AANAT from *Ovis aries*, Q12341 for *Sc*Hat1, and Q03330 for *Sc*Gcn5 from *S. cerevisiae*, G0RNA9 for *Tr*NGS1 from *T. reesei*, A0A1D8PRM0 for *Ca*Ngs1 from *C. albicans*, Q53396 for *Sm*AAC3 from *Serratia marcescens*, and Q47764 for *Ef*AAC6 from *Enterococcus faecium* were aligned. Residues highlighted in red are conserved. Asterisks below the alignment indicate a conserved residue in all aligned sequences. Bold residues represent the start and end of a particular secondary structure since their length varies between species. Blue arrows represent β‐sheets and pink rods α‐helices. Val685 and Gly695 (boxed) in motif A are known to interact with Acetyl‐CoA. Sequences were aligned using EMBOSS online pairwise sequence alignment tool and the structural annotation were retrieved from PDB.

In addition to the transcriptional regulatory gene *ron1* [13], we previously identified another gene jgi|Trire2|79 669 (*nag3*) in *T. reesei* [13] that is conserved and present in most of the GlcNAc gene clusters in Ascomycota (Figs 1E, 2A; Table S3). Similar to the other genes in the cluster, the expression of jgi|Trire2|79 669 is induced on GlcNAc but repressed by glucose in a RON1‐dependent manner [13]. Deletion of the gene resulted in impaired growth on medium containing GlcNAc, but not glucose, whereas overexpression of the gene in the wild‐type (WT) background did not affect growth or colony morphology [13] (Fig. S1, Table S4). The high sequence similarity between the protein encoded by jgi|Trire2|79 669 and glycoside hydrolase (GH) family 3 enzymes suggested a catabolic activity related to GlcNAc containing carbon sources [13]. Interestingly, although Su and collaborators confirmed similarity of the N‐terminal domain of the homolog in *C. albicans* (CaNgs1), they revealed in addition a similarity to an *N*‐acetyltransferase domain in a GCN5‐related histone acetyltransferase family [18]. The ORF of jgi|Trire2|79 669 is 2796 nucleotides long containing six exons which code for a 932 amino acids long protein with a predicted molar mass of 102.11 kDa and pI value of 6.46. To assess the putative function of the protein encoded by jgi|Trire2|79 669, we performed a structure–function prediction. Since a structural model for the closest homolog from *C. albicans* is not available, the secondary structure was predicted using a recently crystallized GH3 multidomain β‐*N*‐acetylglucosaminidase from *Rhizomucor miehei* (RmNag) (Fig. 2B,C; Fig. S2). A pairwise sequence alignment of the full‐length translated ORF of jgi|Trire|79 669 with CaNgs1, RmNag, and EcNagZ showed similarity percentages of 51.2%, 44.2%, and 14.0%, respectively. NagZ cleaves the terminal β‐1,4‐linked *N*‐acetylglucosaminyl residues from peptide‐linked peptidoglycan fragments for recycling of peptidoglycan [28]. The N‐terminal region from amino acids 22–386 of jgi|Trire2|79 669 resembles a β‐*N*‐acetylglucosaminidase with a typical GH3 family (β/α)8 TIM barrel fold. The GH3 domain carries the conserved residues for GlcNAc binding, but, similar to CaNgs1, lacks the nucleophile aspartate and catalytic histidine dyad that is important for enzyme function (Fig. 2B,D; Fig. S3). The β‐*N*‐acetylglucosaminidase homology domain is followed by a subfamily β‐glucosidase domain (amino acids 387 to 599, Fig. S4), which displays α/β/α sandwich fold with glutamic acid as an (acid/base) active site residue. The region from 667 to 717 amino acids is related to the *N*‐acetyltransferase (GNAT) superfamily [29] with a conserved fold consisting of 6 β‐strands and 4 α‐helices (Fig. 2B, represented in yellow). The GNAT region shares structural similarity to histone acetyltransferases (GCN5). The conserved folds of the GNAT superfamily proteins are present and connected in the order β1‐α1‐α2‐β2‐β3‐β4‐α3‐β5‐α4‐β6. The GNAT region protein sequence is further divided into the three structurally conserved motifs A, B, and D, while motif C is not conserved (Fig. 2B,D). The two strands β5 and α4 are present in motif B, β2 and β3 are connected in motif D, and the third strand β4 with a central helix α3 at the C terminus resembles motif A (Fig. 2B,D). Motif A is the most important or core of the GNAT domain [29] and contains all the conserved residues (Leu, Val, Arg, and Gly, Fig. 2D).

In summary, the protein encoded by jgi|Trire2|79 669 lacks the residues for catalytic cleavage of amino sugar substrates. Due to its presence in the GlcNAc gene cluster, its homology to CaNgs1 and our 3D‐modeling, which confirmed a structurally conserved GCN5‐domain in the C terminus, we propose that this enzyme, similar to CaNgs1, may serve as a GlcNAc sensor and not as GH3‐carbohydrate‐active enzyme, as suggested previously [13]. Therefore, we renamed the gene and will refer to it as *ngs1* and NGS1 to its gene product in the following sections.

### Response to *N*‐acetylglucosamine is dependent on RON1 and NGS1

**Fig. 3:**
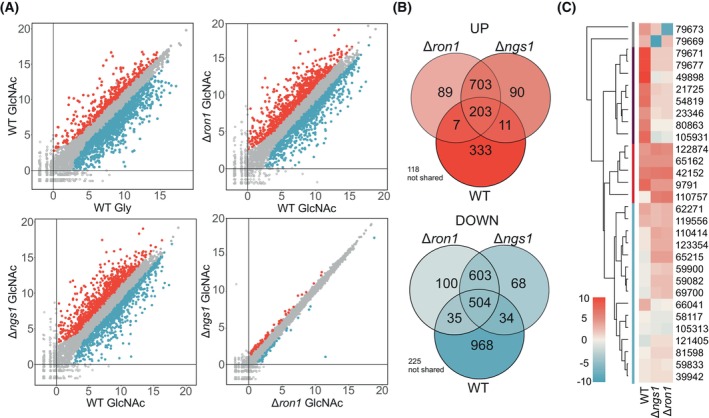
Response to *N*‐acetylglucosamine is dependent on the two regulators present in the GlcNAc gene cluster. (A) Scatter plots comparing log_2_ ratios of counts obtained from wild‐type (WT) and mutant strains (Δ*ron1* or Δ*ngs1*) in the presence of glycerol (Gly) or *N*‐acetylglucosamine (GlcNAc). Upregulated or downregulated genes in the strain shown on the y‐axis are highlighted in red or blue, respectively; gray, not significantly regulated *n* = 3. (B) VENN diagrams comparing the number of shared and unshared up‐ (shades of red) and downregulated (shades of blue) genes in WT (grown on GlcNAc compared with glycerol) and mutant strains (Δ*ron1* or Δ*ngs1*, grown on GlcNAc and compared with WT on GlcNAc). (C) Heat map and hierarchical clustering of differentially expressed genes (DEGs, jgi|Trire2| *gene identifiers*) related to amino sugar metabolism in WT and mutant strains (Δ*ron1* or Δ*ngs1*). The regulator genes (Δ*ron1* or Δ*ngs1*) are indicated with a gray bar at the left. Genes assumed to be related directly to GlcNAc catabolism are marked with a purple bar. Genes assumed to be related to chitin catabolism or chitin metabolism in the cell wall are marked with a red or blue bar, respectively.

To investigate the role of the transcription factor RON1 and the proposed histone acetyltransferase NGS1 in GlcNAc sensing, we analyzed gene expression in single knockout mutants of *ron1* and *ngs1* during growth on GlcNAc compared with glycerol in a transcriptomic approach. The respective knockout mutants were created in previous work [13] and are listed in Table S4. Phenotypes of a replacement strain (*ron1* ^+^RPM) and an *ngs1*‐overexpression (oe‐*ngs1*) strain are indistinguishable from the WT parental strain (Fig. S1). Remarkably, the absence of either one of the genes leads to a dramatic change in the expression profile following transfer to GlcNAc as sole carbon source (Fig. 3; Tables S5 and S6). First, a 180% increase in upregulated genes (Δ*ngs1*: 1034, Δ*ron1*: 1024) and a 20% reduction of downregulated genes (Δ*ngs1*: 1210, Δ*ron1*: 1242) compared with the parental strain QM9414 Δtku70 was observed. Second, a strong overlap of the regulatory activities of RON1 and NGS1 was evident, as demonstrated by regression‐ and Venn‐analysis (Fig. 3A,B). The mutants shared 1306 co‐regulated genes, but only 707 genes were co‐regulated between the WT and both mutants after transfer to GlcNAc. Within the KOG category related to carbohydrate metabolism and transport, genes from the GlcNAc gene cluster were among the most strongly downregulated genes in the knockout strains (Fig. 3C). Differential regulation was especially strong for the gene encoding the glucosamine deaminase, *dam1* (jgi|Trire2|49 898), and the gene putatively coding for the GlcNAc transporter, *ngt1* (jgi|Trire2|80 863). These findings show that the GlcNAc catabolic genes are equally dependent upon two regulators, RON1 and NGS1 (Fig. 3C) [13]. Additional 25 DEGs related to amino sugar metabolism but not present in the GlcNAc gene cluster were differentially regulated upon deletion of either of the regulators (Fig. 3C). In addition to the *N*‐acetylglucosaminidases (glycoside hydrolase GH20) NAG1 (jgi|Trire2|21 725) and NAG2 (jgi|Trire2|23 346) [13, 30], a third gene (jgi|Trire2|105 931) encoding a GH20 family enzyme with a transmembrane domain showed strong dependence on positive regulation by NGS1 and RON1 (Fig. 3C, purple bar). Five additional genes related to chitin catabolism were slightly upregulated in the Δ*ngs1* and Δ*ron1* strains (Fig. 3C, red bar). Moreover, genes categorized in chitin metabolism (chitinases, pred. chitinases, chitosanases) and genes that have been proposed to be involved in cell wall metabolism or pathogenicity were differentially regulated in both knockout strains (Fig. 3C, blue bar).

Apart from their regulatory function in amino sugar catabolism, deletion of *ngs1* or *ron1* impacted the differential gene expression in all over‐represented KOG categories (Fig. 1). The number of upregulated genes increased strongly (Tables S5, S6, S11, S12) with one exception, genes related to ‘amino acid transport and metabolism’ were not strongly affected. ‘Carbohydrate metabolism and transport’, ‘Secondary metabolites biosynthesis transport and catabolism’, and ‘Lipid transport and metabolism’ were the most affected categories with around 180% increase in upregulated genes and a decrease by 50–60% in downregulated genes. Thus, our findings indicate that, in the presence of GlcNAc, RON1, and NGS1, might partially act as negative regulators or repressors for two‐thirds of the genes of the investigated KOG classes. However, both are crucial for activating genes related to GlcNAc catabolism and the differential regulation of a subset of chitin catabolic and chitin metabolic genes related to cell wall metabolism and pathogenicity.

### CRE1 is a repressor of structural GlcNAc gene expression

**Fig. 4:**
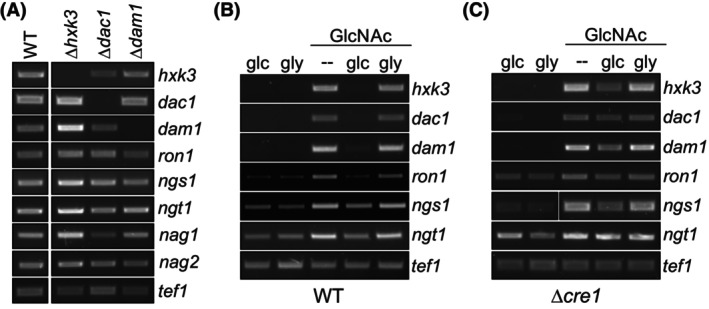
CRE1 is a repressor of structural GlcNAc gene expression. (A) Gene expression analysis (semi‐quantitative RT‐PCR) in the structural gene knockout strains. Expression of GlcNAc cluster genes, *ngt1*, and the genes encoding for the *N*‐acetylglucosaminidases *nag1* and *nag2* after replacement to 1% GlcNAc for 3 h was assessed. *tef1* was used as reference gene. (B, C) Gene expression analysis (semi‐quantitative RT‐PCR) of the GlcNAc cluster genes and NGT1 after growth for 24 h on medium supplemented with 1% glucose (glc), 1% glycerol (gly), or 1% GlcNAc either alone or combined with glc or gly. *tef1* was used as reference gene. Expression was analyzed in (B) the wild‐type (WT) or (C) the carbon catabolite derepressed Δ*cre1* mutant. Representative gels from two independent experiments are shown.

In *C. albicans*, GlcNAc is phosphorylated by a hexokinase immediately after import to enter metabolism [1, 24]. In *T. reesei*, HXK3 was identified as the enzyme catalyzing the transfer of the phosphate group to the amino sugar, and deletion of *hxk3* leads to a GlcNAc catabolic deficiency phenotype [13]. The absence of *dac1* (encoding a deacetylase) or *dam1* (encoding a GlcN‐phosphate isomerase) negatively impacted the ability to use GlcNAc as carbon source [13]. In *N. crassa*, growth on GlcNAc is inhibited even in the presence of an alternative carbon source, probably due to a dysfunctional *dam1* gene [12]. Therefore, we investigated interdependence of transcript levels in the single GlcNAc catabolic gene knockout strains (Δ*hxk3*, Δ*dac1*, and Δ*dam1*) in the presence of GlcNAc, and compared it with the parental strain (Fig. 4A) in a carbon source replacement experiment with 3 h of induction by 1% GlcNAc. The genes were transcribed in all of the tested knockout strains, and transcript levels in the Δ*hxk3* mutant were slightly elevated when compared to the parental strain. Similarly, *nag1* and *nag2*, as well as *ngt1* and *ron1* levels, were not influenced (Fig. 4A).

A second question concerns the impact of repressors on activation of the GlcNAc catabolic genes. In the presence of the preferred carbon source glucose, expression of genes encoding for other carbon catabolic pathways is often downregulated, referred to as carbon catabolite repression (CCR). In *T. reesei* and many other fungi, one of the regulators of CCR is the transcription factor CRE1 [31, 32]. To determine the dependence of GlcNAc catabolism on CCR, we investigated the expression of catabolic genes on GlcNAc in the presence of glucose, a strong repressing carbon source. Interestingly, glucose, but not glycerol, led to a strong reduction in expression of the structural genes (*hxk3*, *dac1*, *dam1*) 24 h after the addition of the carbon source to medium containing GlcNAc (Fig. 4B). Transcript levels of *ron1*, *ngs1*, and *ngt1* were not markedly decreased under both conditions. By contrast, in the Δ*cre1* strain, expression of the structural genes was derepressed on medium containing both GlcNAc and glucose (Fig. 4C). Therefore, the absence of any of the structural genes (*hxk3*, *dac1*, and *dam1*) does not negatively affect the expression of the remaining genes, their regulators or *ngt1*. Notably, the expression of the structural genes is repressed by the presence of glucose but is derepressed in absence of *cre1*, while the expression of the GlcNAc transcriptional regulators or *ngt1* is not strongly affected.

### GlcNAc transport and sensing

In our previous study, we showed that the lack of *ngt1* in *T. reesei* almost completely abolished biomass formation in submerged cultivations on GlcNAc, indicating that NGT1 is the major GlcNAc transporter in *T. reesei* [13]. In *T. reesei*, *ngt1* is located distantly from the GlcNAc gene cluster on chromosome 7 [15, 16]. In this work, we wanted to get a deeper insight into the role of NGT1 in GlcNAc sensing.

### NGT1 is a checkpoint for GlcNAc catabolism

**Fig. 5:**
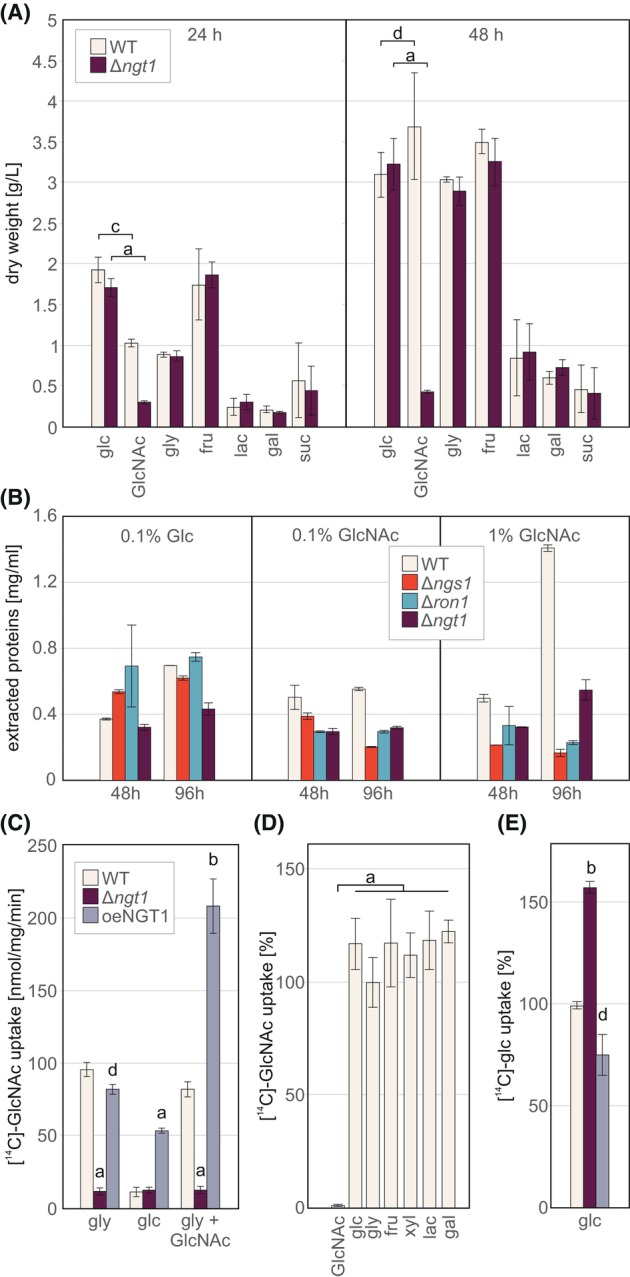
NGT1 is the only strong specific importer of GlcNAc in *T. reesei*. (A) Biomass formation of the wild‐type (WT) and the Δ*ngt1* mutant on 1% of different carbon sources for 24 h and 48 h. Sugars represented in (A) and (D) are glucose (glc), *N*‐acetylglucosamine (GlcNAc), glycerol (gly), fructose (fru), lactose (lac), galactose (gal), sucrose (suc), and xylose (xyl). (B) Biomass formation on 0.1% carbon sources (glc or GlcNAc) compared with 1% GlcNAc of the WT compared with the mutant strains Δ*ngs1*, Δ*ron1*, or Δ*ngt1*. (C) [^14^C]‐GlcNAc uptake in the WT, the Δ*ngt1* mutant or a *ngt1* overexpression strain (*oe‐ngt1*). Strains were grown on glucose (glc) or glycerol (gly) and shifted to 0.1% GlcNAc for 30 min (gly + GlcNAc) prior to the assay. (D) [^14^C]‐GlcNAc uptake in the WT in the presence of excess competitor sugars as indicated. (E) [^14^C]‐glucose uptake in WT, the Δ*ngt1* mutant or *oe‐ngt1*. The mean and standard deviation of two biological and technical replicates are shown. a, b, c, and d indicate significance at *P* < 0.001, 0.005, 0.01, and 0.05 according to student's *t*‐test, respectively.

In agreement with our previous results, growth on GlcNAc was significantly reduced in Δ*ngt1* strains, whereas the parental strain showed even higher biomass accumulation after 48 h on GlcNAc than on glucose (Fig. 5A) [13]. Growth of the *ngt1* deletion strain on all other tested carbon sources (glycerol, fructose, lactose, galactose, sucrose, and cellobiose) did not significantly affect biomass formation as compared to the parental strain (Fig. 5A).

When grown on solid minimal medium with 1% GlcNAc as sole carbon source, the mycelial density of *ngt1* deletion strains was decreased compared with the control [13]. In *S. cerevisiae*, other endogenous hexose transporters can compensate for the specific GlcNAc transporter when high concentrations of GlcNAc are present [33]. Therefore, we tested the growth on a lower concentration of GlcNAc. Due to the low mycelial density created during growth on low concentrations of a carbon source, intracellular extracted proteins were used as a measure of biomass formation, as established previously [34]. With 0.1% (4.5 mm) GlcNAc, the biomass formation of Δ*ngt1*, Δ*ngs1* and Δ*ron1* was more strongly delayed compared with the WT control strain and to 1% GlcNAc (Fig. 5B). Interestingly, on 1% GlcNAc, where almost no growth of Δ*ngs1* and Δ*ron1* is detected, Δ*ngt1* showed less growth reduction (Fig. 5B). This suggests that, as seen for *S. cerevisiae*, other hexose transporters with a lower affinity for GlcNAc might compensate for NGT1 when GlcNAc is present in high concentrations. In *Histoplasma capsulatum*, a second MFS transporter (NGT2) was identified that could partially compensate for HcNgt1 [8]. The expression of HcNgt2 was slightly upregulated in the presence of GlcNAc and was required for filamentation in the dimorphic yeast [8]. A blast search with *C. albicans* CaNgt1 or *H. capsulatum* HcNgt1 and HcNgt2 in the *T. reesei* database showed TrNGt1 as the first hit with a pairwise similarity of 65.8% to CaNgt1, 57.8% to HcNgt1 and 40.3% to HcNgt2 (Table S7). The blast search yielded three additional putative MFS transporters (jgi|Trire2|64 049, jgi|Trire2|76 713, jgi|Trire2|112 034; Table S7), but with lower sequence similarity than TrNGT1 to each of the queries. Moreover, RNA‐seq data revealed a downregulation of these three genes in the WT strain in the presence of GlcNAc compared with glycerol (Fig. S5). In the regulatory mutant strains (Δ*ron1* and Δ*ngs1*), the expression of these putative MFS transporters did not compensate for the downregulation of *ngt1* expression (Fig. S5). Thus, a compensatory role for GlcNAc uptake by these putative *T. reesei* MFS transporters is unlikely.

### NGT1 is the only strong specific importer of GlcNAc in *T. reesei*

In *Candida* spp., NGT1 is the only GlcNAc importer and GlcNAc needs to be taken up to induce GlcNAc catabolism and virulence gene expression [23, 24]. To monitor GlcNAc import in *T. reesei*, we first performed uptake assays using radiolabelled [^14^C]‐GlcNAc when the fungus was grown either on glycerol or glucose. In the presence of glycerol, *T. reesei* was able to import ca. 100 nmol GlcNAc per mg cell dry weight per minute, while the import was nearly abolished when the strain was grown on glucose (Fig. 5C). These findings indicate that glucose, which does not completely block *ngt1* expression (Fig. 4B), may negatively influence GlcNAc uptake further by an alternative mechanism, such as glucose inhibition or catabolite inactivation of the transporter. In contrast to the parental strain, the Δ*ngt1* mutant showed nearly abolished GlcNAc import in the presence of both carbon sources, glycerol or glucose (Fig. 5C). To gain further insights into the role of NGT1, we constructed an overexpression strain with an extra copy of *ngt1* integrated ectopically in the parental strain QM9414, under control of a *tef1*‐promoter and with an eGFP sequence at the 3′ end (see Materials and Methods for details). The phenotype of oeNGT1‐GFP on PDA, and minimal medium containing 1% glucose or GlcNAc was similar to the WT (Fig. S1). Interestingly, the strong, constitutive overexpression of NGT1 in the WT background was partially able to compensate for the inhibition by glucose (Fig. 5C). The addition of 0.1% GlcNAc to the glycerol containing medium 30 min before the uptake assay doubled the uptake to over 200 nmol/mg/min (Fig. 5C), indicating that both the integrated extra copy of oeNGT1‐GFP and the native NGT1 in this mutant significantly contribute to GlcNAc uptake. To analyze the specificity of NGT1 for GlcNAc, we monitored import of [^14^C]‐GlcNAc in the parental strain in the presence of excess competing nonlabeled sugars (20 mm). Only excess amounts of GlcNAc and none of the other sugars significantly outcompeted the import of [^14^C]‐GlcNAc, indicating that NGT1 is highly specific for GlcNAc (Fig. 5D). Glucose is transported by various sugar‐porters into the cell, and many sugar‐porters with high specificity for the respective sugar can also partly import glucose. Therefore, we investigated whether NGT1 has an affinity for glucose. The uptake assay using [^14^C]‐glucose instead of GlcNAc revealed increased import of glucose in the Δ*ngt1* mutant and a slight decrease of import in the oeNGT1‐GFP strain, in comparison to the WT (Fig. 5E). Consequently, NGT1 does not appear to participate in glucose uptake but the data suggest that its presence might negatively impact import of glucose.

### Expression of the catabolic genes is not influenced by deletion of *ngt1* at high GlcNAc levels

**Fig. 6:**
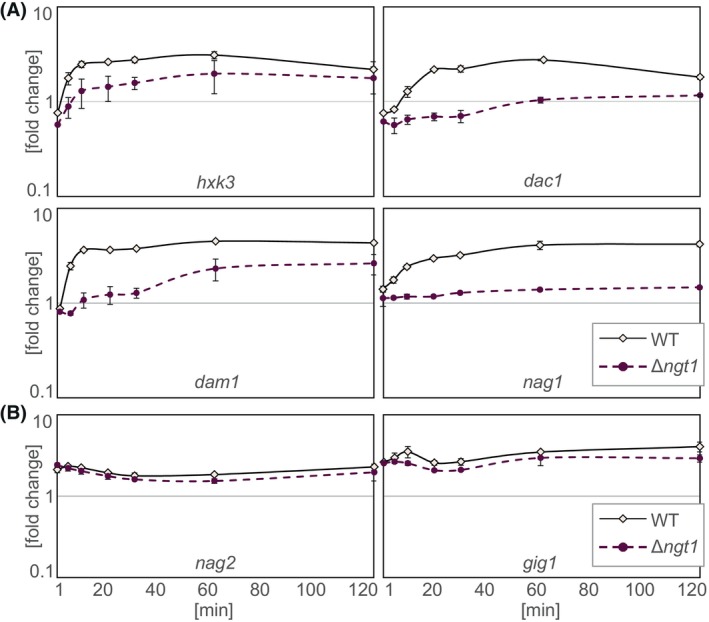
Expression of the catabolic genes is not influenced by deletion of *ngt1* at high GlcNAc levels. (A) Fold change of semi‐quantitative expression analysis of catabolic genes from wild‐type (WT) and Δ*ngt1* grown on minimal medium containing 0.1% GlcNAc and compared with 0.1% glucose. (B) Fold change of semi‐quantitative expression analysis of genes, related to virulence in other ascomycetes species [35, 36], from WT and Δ*ngt1* grown on minimal medium containing 0.1% GlcNAc and compared with 0.1% glucose. Standard deviation from two independent experiments is shown.

GlcNAc is a potent inducer of approximately 500 genes, which is dependent upon the transcriptional activators RON1 and NGS1. This regulation could proceed via two potential routes: (a) GlcNAc may be sensed by a receptor that is located in the cell wall/plasma membrane, such as a G‐protein coupled receptor, and the signal is then transduced intracellularly to the transcriptional machinery, or (b) GlcNAc needs to be taken up into the cell to signal its availability as described for *C. albicans* [23]. To test these two possibilities, we performed a time course analysis using the parental and the Δ*ngt1* strain. Since Δ*ngt1* strains exhibit impaired growth on medium containing GlcNAc as sole carbon source, the strains were pregrown on 1% glycerol for 24 h and shifted to either 0.1% glucose or 0.1% GlcNAc. Expression of the GlcNAc cluster genes was then monitored from 1 to 120 min after c‐source replacement and a fold change was calculated by comparing the expression at the indicated time on GlcNAc with glucose. Interestingly, Δ*ngt1* showed a significant delay in the initiation of catabolic gene expression of all three tested GlcNAc cluster genes compared with the parental strain (Fig. 6A). While the parental strain showed strong and stable expression of the catabolic genes after 20 min, Δ*ngt1* did not reach the same expression levels even after 120 min (Fig. 6A). Conversely, when 1% GlcNAc was used, the expression was already similar to WT levels after 10 min (Fig. S6A,B). Thus, we conclude that only intracellular GlcNAc induces gene expression and only when GlcNAc is present in higher concentrations other transporters may potentially compensate for the loss of NGT1 and induce expression of GlcNAc cluster genes in the Δ*ngt1* strain. These findings align with observations in *C. albicans*, where GlcNAc needs to enter the cell to induce transcription of the catabolic genes [23]. Similarly, the expression of genes encoding upstream enzymes, such as the *N*‐acetylglucosaminidase NAG1 that mobilizes GlcNAc from chitin, is induced only after 20 min, and *nag1* expression does not reach WT levels after 120 min on 0.1% GlcNAc in the *ngt1* deletion strain (Fig. 6A).

By contrast, the expression of *nag2*, encoding a β‐*N*‐acetylglucosaminidase related to mycoparasitism in some *Trichoderma* spp. [30, 35], remained constantly high after 1 min of addition of 0.1% GlcNAc (Fig. 6B). This is not due to constitutive expression of *nag2*, as 1% glucose or 1% glycerol did not lead to *nag2* transcription (Fig. S6C), in line with findings for orthologs in *T. atroviride* [30]. Moreover, the expression of a gene previously identified as highly activated by GlcNAc and important for virulence in *C. albicans*, *gig1* [36], was strongly increased in *T. reesei* just 1 min after the addition of 0.1% GlcNAc (Fig. 6B). Thus, we propose that expression of genes related to GlcNAc catabolism depends on the import of GlcNAc, while the expression of other genes, such as *nag2* or *gig1*, appears to be independently regulated, probably already by external GlcNAc. These findings diverge from previous observation in *C. albicans* and *H. capsulatum* [8, 23].

## Discussion

Using *T. reesei* as a model organism for filamentous soil fungi, we investigated the impact of GlcNAc on growth and gene expression and identified key elements of their GlcNAc sensing apparatus.

The expression of over 2000 genes was influenced in *T. reesei* by GlcNAc. The presence of two regulators, *ron1* and *ngs1*, in the GlcNAc gene cluster is important for differential regulation of two‐thirds of the GlcNAc responsive genes. We demonstrated that these regulators play a role in GlcNAc sensing similar to *C. albicans* CaRep1 (RON1 homolog) and CaNgs1 [18], respectively. The Ndt80‐like transcription factor RON1 has previously been described as a regulator of the GlcNAc gene cluster [13]. Here, we characterized TrNGS1 in detail. TrNGS1 contains an N‐terminal GH3‐domain with similarity to bacterial NagZ [13]. Another GH3 β‐N‐acetylglucosaminidase with an additional GNAT domain, was previously crystallized from *Rhizomucor miehei* (RmNag), and its structure was solved at 2.3 Å resolution [37, 38]. RmNag served as structural model for TrNGS1. By contrast to RmNag, the highly conserved DSH triad in the β‐*N*‐acetylglucosaminidase domain (sequence identifier: K‐H‐(FI)‐P‐G‐(HL)‐G‐x(4)‐D‐(ST)‐H) that is important for the hydrolase activity of GH3‐family proteins is not conserved in TrNGS1 (G‐S‐N at position 206–208). Litzinger and collaborators reported that mutating Asp to Gly in NagZ of *Bacillus subtilis* reduced the catalytic efficiency but the substrate affinity for 4‐Mu‐β‐GlcNAc increased [39]. Furthermore, in TrNGS1, the nucleophile aspartate at position 282 is exchanged for glutamate. Even though glutamate can perform a similar role to aspartate in the catalytic site of proteases and lipases, this substitution can significantly decrease hydrolase activity. This implies that the catalytic β‐*N*‐acetylglucosaminidase site is inactive in TrNGS1, similar to CaNgs1 [18]. However, increased GlcNAc affinity might benefit TrNGS1, if it acted as a GlcNAc sensor. Contrary to CaNgs1, *T. reesei* harbors a second domain identified as β‐glucosidase which contains Glu_465_ as the putative acid/base catalyst, suggesting that TrNGS1 might have retained glucosidase activity. Finally, the C terminus of the TrNGS1 shares similarity to GNAT domains. Both, CaNgs1 and RmNag, exhibit histone acetyltransferase activity from a GNAT‐domain in the C terminus [18, 38]. CaNgs1 is recruited by CaRep1 (the TrRON1 homolog) to the promotor region, where acetyl‐residues at histone H3 are introduced by CaNgs1 [18]. The transferase activity for RmNag onto GlcN monomers to create GlcNAc has been shown *in vitro* in *R. miehei*, while a GNAT activity for histones was not investigated due to a divergent consensus sequence [38]. GNAT members universally share a conserved domain structure, scaffolded in four motifs [29]. In TrNGS1, motifs A, B, and D are structurally conserved, and most of the residues important for binding and catalytic activity are present in motif A. However, these catalytic residues are not conserved in the GNAT superfamily. For instance, while His_700_ is present in CaNgs1 most other members, including AANAT (*Ovis aries*), AAC6′ (*E. faecium*), and Gcn5 (*S. cerevisiae*) have glutamate at the analogous position [18, 29]. On the contrary, the corresponding residues in Hat1 (*S. cerevisiae*) and AAC3 (*S. marcescens*) are substituted with lysine and tyrosine, respectively [40], and these residues are unlikely to act as a base. In this scenario, amino acids near the active site presumably perform the deprotonation and act as a general base, such as the respective residues Asp_255_ and Asp_110_ in Hat1 and AAC3 and Asp_686_ in TrNGS1. Our findings suggest that TrNGS1 lost its β‐*N*‐acetylglucosaminidase function, but we cannot rule out a β‐glucosidase activity. Furthermore, the C‐terminal region strongly resembles a histone acetyl transferase domain, suggesting that TrNGS1 has a similar function as GlcNAc signal sensor‐transducer as described for CaNgs1 [18].

Our data confirmed that the presence of GlcNAc has the strongest impact on the expression of genes related to GlcNAc catabolism [13] and showed that they are strictly dependent on RON1 and NGS1. These findings are in line with results in *C. albicans* [18]. In addition, the increased expression of the three β‐*N*‐acetylglucosaminidases (GH20) NAG1, NAG2 [30], and jgi|Trire2|105 931 [41] is strictly dependent on both regulators. Furthermore, approximately one‐third of the genes related to the KOG class carbohydrate metabolism were differentially regulated by GlcNAc, including a high number of glycoside hydrolases (GH). Here, we show that GlcNAc is an efficient carbon source for *T. reesei* with slightly higher biomass formation than after prolonged growth on glucose. This indicates that the presence of GlcNAc signals the availability of an easily utilizable carbon and nitrogen source, induces expression of genes directly involved in GlcNAc catabolism, and concomitantly reduces expression of unrelated carbon catabolism pathways. Interestingly, genes with predicted chitinase function and a GH75 chitosanase gene, *cho3* [42], were also upregulated in a RON1/NGS1 independent manner. On the contrary, 24 genes assigned to the KOG category cell wall metabolism were differentially regulated in the presence of GlcNAc, indicating that GlcNAc sensing and/or catabolism impacts cell wall plasticity. Moreover, the regulation of a class II hydrophobin (jgi|Trire2|106 538) and genes related to GlcNAc metabolism and pathogenicity, such as, CHI18‐15 [43, 44], CDA4, a chitin deacetylase implicated in self‐/non‐self‐recognition [42], and a homolog of the *C. albicans* virulence gene *GIG1* [36] depends on RON1 and NGS1. In *M. oryzae* MoNdt80 is related to GlcNAc catabolism but is also involved in pathogenesis [22].

Remarkably, secondary metabolism genes were the third most GlcNAc regulated KOG class, with one‐third up‐ and two‐thirds downregulated genes. These findings are consistent with data from primary sugar catabolism, which impacts beta‐lactam biosynthesis by carbon catabolite repression. Primary sugars, such as glucose or sucrose, downregulate entire secondary metabolism gene clusters [45, 46]. Furthermore, deletion of related Ndt80‐like transcription factors, such as *Aspergillus nidulans xprG*, strongly reduces secondary metabolites, such as characteristic pigments, sterigmatocystin and penicillin [47]. On the contrary, glucose and sucrose also negatively impact the expression of genes related to metabolism of other carbon sources. Our data are a first hint that GlcNAc catabolic genes are negatively regulated by glucose in a *cre1* dependent manner. Interestingly, the homolog of VIB1 (jgi|Trire2|54 675) was upregulated in *T. reesei* by GlcNAc. VIB1 next to RON1 is a member of the VIB1/XprG group of Ndt80‐like transcription factors and is involved in heterokaryon incompatibility and cellulase production in *T. reesei* [48]. In *N. crassa*, VIB‐1 is required to produce several CAZymes, including cellulase, and is an upstream negative regulator of CRE‐1 [49]. Katz recently proposed that the ancestral role of Ndt80 like transcription factors is based on nutrient sensing [50]. Our data corroborate this hypothesis and show that different sensors may respond to the same nutrient source to induce or repress the expression of whole signaling pathways. Moreover, regulators with a central role in fungal physiology are conserved throughout the fungal tree of life but their functions may have diversified [51, 52, 53].

To investigate whether and how GlcNAc enters the cell for signaling we further characterized the major facilitator protein NGT1 and showed its high specificity and affinity for GlcNAc. NGT1 is an MFS transporter, and MFS members are found throughout all kingdoms of life and represent an ancient group of secondary carriers. They are the largest family of uniporters, symporters, and antiporters [54, 55, 56, 57]. GlcNAc transporters are highly specific for import of the extracellular aminosugar [23]. NGT1 is conserved in fungi with an intact GlcNAc gene cluster, and in *T. reesei*, NGT1 shows 65.8% pairwise similarity with its ortholog in *C. albicans*. The role of NGT1 seems to be conserved even in higher organisms, such as plants, since an ortholog of NGT1 has been identified in rice [58]. Similar to CaNgt1 [23], low concentrations of GlcNAc are sufficient for activation of gene expression. 4.5 mm GlcNAc were sufficient for induction of gene expression in *T. reesei* WT within 1 min and 1.96 μm GlcNAc sufficed to activate visible CaNgt1‐GFP expression [59]. Deletion of *T. reesei ngt1* negatively impacts growth on GlcNAc [13]. The residual growth of the Δ*ngt1* strain on solid medium with high amounts of GlcNAc could be attributed to the inefficient incorporation of GlcNAc via other hexose transporters. Although *S. cerevisiae* is not capable of utilizing GlcNAc as a carbon source due to a complete loss of the GlcNAc gene cluster and the *ngt1* ortholog [60], other hexose transporters can transport small amounts of GlcNAc into the cell [33].

An important finding that distinguishes the GlcNAc signaling pathway of *T. reesei* from *C. albicans* is that NGT1 seems to be dispensable for activation of some of the GlcNAc‐related catabolism genes, which are not part of the GlcNAc gene cluster. In *C. albicans* GlcNAc import is a prerequisite for activation of expression of the catabolic genes [19, 20, 61], which we confirmed also for *T. reesei*. Importantly, in contrast to *C. albicans*, already within the first minute of GlcNAc addition a set of genes related to GlcNAc and pathogenicity, such as *nag2* or *gig1* were induced in *T. reesei* [36, 62]. NAG1 and NAG2 are mycoparasitism‐related genes, and play a role in chitin and chitobiose degradation, but are not associated with starvation or cell wall recycling [30, 35]. NAG1 is a secreted enzyme, whereas NAG2 was found to be cell wall associated [30, 41]. Interestingly, in contrast to expression of *nag1*, which showed stable induction only after 20 min of GlcNAc addition, *nag2* expression was activated within a minute by GlcNAc. This supports the hypotheses drawn in previous work that NAG1 does not function as extracellular sensor for GlcNAc or chitin. In the absence of NAG1, the chitinase activity was strongly decreased, but not in the Δ*nag2* or the double knockout strain [30]. According to Ramot and collaborators [63], *T. asperellum* has the ability to store a high amount of the NAG1 homolog in an active form in the fungi before it is secreted into the medium.

It should be mentioned that the previous data showed carbon starvation for 5 h or longer impacted expression of some of the GlcNAc cluster genes considerably (ca. 50 × fold increase of *dac1‐* and *ngt1* expression) in a *ron1* dependent manner, while only a minor impact on the other GlcNAc cluster genes was observed [13]. In this study, gene expression analysis involving single knockout strains of GlcNAc cluster genes and *ngt1* was therefore assessed in carbon source replacement experiments to avoid prolonged carbon starvation. Nevertheless, carbon starvation in those mutants that are not able to utilize GlcNAc as carbon source could possibly contribute to differential regulation of some of the genes. In the case of *ngt1*, the overall low and strongly delayed expression of the GlcNAc cluster genes does not hint at a carbon starvation event. Thus, our data provide evidence that *T. reesei* may be able to distinguish extracellular from intracellular GlcNAc by a mechanism that is yet to be identified, but which seems to be important for inducing expression of different sets of genes related to, for example, primary metabolism or defense.

In conclusion, we propose that GlcNAc signaling is a common trait among filamentous soil fungi. Similar to findings from dimorphic yeasts and bacteria, GlcNAc may be crucial in morphogenetic programs, mating, stress, and pathogenicity in filamentous soil fungi. Particularly, in the natural environment of soil‐dwelling fungi, GlcNAc is highly abundant due to its structural importance in bacteria, fungi, and insects. Consequently, soil fungi, such as *Trichoderma* spp., encounter GlcNAc frequently, potentially signaling the presence of nutritious resources, hosts, or competitors, thereby inducing several signaling cascades related to metabolic processes, stress, and defense reactions. Our findings contribute to the understanding of sugar metabolism and sensing in filamentous soil fungi, which in turn might impact microbiome composition and thus soil fertility.

## Materials and methods

### Strains and cultivation conditions

*Trichoderma reesei* QM9414 (ATCC 26921) and overexpression mutants constructed in this work, the knockout mutants [13], and their parental strain *T. reesei* QM9414 Δ*tku70* (C. Ivanova and B. Seiboth; unpublished and [64]) were maintained on potato dextrose agar (PDA, BD, Franklin Lakes, USA) and are listed in Table S4. Two independent clones from each mutant strain were used in all experiments. Stock cultures were kept in 50% glycerol at −80 °C. Mandels‐Andreotti (MA) medium with 0.05% peptone was used for all cultivations (1.4 g·L^−1^ (NH_4_)_2_ SO_4_, 2.0 g·L^−1^ KH_2_PO_4_, 0.3 g·L^−1^ MgSO_4_·7H_2_O, 0.3 g·L^−1^ CaCl_2_·2H_2_O, 5 mg·L^−1^ FeSO_4_·7H_2_O, 1.6 mg·L^−1^ MnSO_4_·H_2_O, 1.4 mg·L^−1^ ZnSO_4_·7H_2_O, and 2 mg·L^−1^ CoCl_2_·2H_2_O (adapted from [65])), and culture conditions were standardized at 28 °C and incubation without a light source throughout all experiments. Different carbon sources were added at 0.1% or 1% concentration as indicated in the manuscript: d‐Glucose Monohydrate, glycerol and cellobiose (Carl Roth GmbH + Co. KG, Karlsruhe, Germany); *N*‐acetylglucosamine (GlcNAc), d‐galactose, and sucrose (Sigma‐Aldrich, St. Louis, MO, USA); d‐fructose and lactose (Calbiochem, Merck, Darmstadt, Germany). Cultivation in liquid standing culture in small petri dishes (3 cm diameter) or shake flask cultivation at 220 rpm using Erlenmeyer flasks sealed with sterile wound dressings were performed as indicated in the manuscript. 1 × 10^6^ conidia·mL^−1^ were used to start all cultivations. For assessing expression in mutant strains that do not support growth on GlcNAc as sole carbon source, mutant strains and their respective biological controls were cultured by carbon source replacement, as described previously [13]. The strains were first precultured for 16–24 h in liquid shake flask culture on 1% glycerol, harvested by filtration through Miracloth (Calbiochem, Merck), washed carefully with water, and transferred to fresh MA medium, containing 0.1 or 1% of the indicated carbon sources or combinations thereof (glycerol, glucose, or GlcNAc) and cultured for an indicated additional time. These steps were performed under sterile conditions, to prevent contamination during the transfer from one carbon source to the other. Mycelial dry weight was determined by withdrawing 50 mL aliquots from shake flask cultivations, suction filtration through a glass wool filter disk (Whatman, Maidstone, UK), followed by extensive washing with water, and drying at 80 °C to constant weight. For determining total protein as a determination of cell growth from small volumes, the method described in [34] was used. Briefly, from liquid standing cultivation in small Petri dishes (3 cm diameter), mycelium (including insoluble carbon sources) was harvested after the given incubation times by filtration, using Miracloth (Calbiochem, Merck) placed in small funnels, followed by extensive washing with water. Proteins were extracted by incubation for 3.5 h in 1 m sodium hydroxide at RT. The extracted proteins were diluted with water and measured in 96‐well microplates following a procedure based on the Bradford method [66] and a commercial protein assay reagent (Bio‐Rad Laboratories, Hercules, CA, USA). Bovine serum albumin was used as standard.

### Generation of overexpression mutants

An *ngt1*‐overexpression construct was cloned using the vector pRBVII as backbone. Full‐length *ngt1* (jgi|Trire2|80 863) was integrated into the vector downstream of 750 bp of the *T. reesei*‐*tef1*‐promotor region (jgi|Trire2| 300 828) and in frame with a C‐terminal eGFP sequence. An *ngs1*‐overexpression construct was cloned using the vector pPcdna1 as backbone [67]. Full‐length *ngs1* (jgi|Trire2|79 669) was integrated into the vector downstream of the *T. reesei*‐*Cdna1*‐promotor region (jgi|Trire2|110 879). The vectors were constructed using the yeast recombination system described in [68] with the primers listed in Table S7. The constructs were ectopically integrated in *T. reesei* QM9414 by protoplast transformation essentially as described in [69]. Integration of the constructs was verified by PCR using primers Tr_NGTL_RTs and GFP_ctrl_a for oeNGT1‐GFP and primers CTRL_cDNA1s and Tr_GH3_RTa for oeNGS1 (listed in Tables S8 and S9) after two rounds of purification by single spore isolation on selective medium containing 100 μg·mL^−1^ hygromycin B (Roth).

### Isolation and purification of fungal DNA

Genomic DNA from *T. reesei* was isolated for cloning using the Wizard Genomic DNA Purification Kit (Promega, Madison, WI, USA). For verification of knockouts, genomic DNA was isolated using a rapid DNA purification protocol [70].

### RNA‐seq analysis

For RNA‐seq analysis, the parental strain QM9414 Δ*tku70*, the Δ*ron1* and Δ*ngs1* mutant strains were cultured by carbon source replacement from 24 h pregrowth on 1% glycerol to either 1% glycerol or 1% GlcNAc followed by incubation for another 3 h in liquid shaking culture. Mycelial samples from biological triplicates were harvested, washed with cold distilled water, and immediately frozen in liquid nitrogen. For RNA isolation, the samples were ground in liquid nitrogen and total RNA was isolated using the guanidinium thiocyanate method [71]. Illumina stranded TruSeq RNA library preparation, including poly(A) enrichment RNA isolation (Microsynth, Balgach, Switzerland) and sequencing on an Illumina NextSeq, v2, 1 × 75 bp was performed. After demultiplexing and trimming of Illumina adaptor residuals, the Illumina reads were mapped against the published *T. reesei* QM6a genome (v.2.0; http://fungi.ensembl.org/Trichoderma_reesei/Info/Index) using tophat (2.1.0) and the overall transcriptional activity was determined for each gene by calculating the number of reads per kilobase of exon model per million mapped reads (RPKM). R (Version 4.2.1) with the bioconductor package for DESeq2 was used for differential expression analysis employing a threshold of adjusted *P*‐value *P*adj <0.05 and a log_2_ fold change of ≥1.0 or ≤−1.0 [72]. A summary of all genes with detectable levels of expression is presented in Tables S1, S5 and S6. A full list of RNA‐seq data can be found in Tables S10–S12.

### Gene expression analysis

For RNA isolation for semi‐quantitative RT‐PCR, the samples were ground twice with a retch mill shaker for 2 min at a frequency of 30 shakes per second and total RNA was isolated using the guanidinium thiocyanate method [71]. Isolated RNAs were treated with DNAse I (Fermentas, St Leon‐Roth, Germany), and cDNAs were subsequently generated with the Revert Aid H‐minus cDNA synthesis kit (Fermentas). RT‐PCR was performed using the gene‐specific primers listed in Table S9. The *tef1* gene (jgi|Trire2|46 958) was used as internal reference. Representative images from gel electrophorese of one out of three experiments are depicted. For semi‐quantitative assessment of expression, the PCR products from at least two independent experiments were quantified using imagej software (https://imagej.net/ij/download.html).

### Sugar uptake assay

The assay was adapted from [73]. *T. reesei* strains were grown in liquid shaking culture in MA containing either 1% glycerol or 1% glucose. To investigate the effect on GlcNAc uptake by preincubation with the amino sugar, selected samples cultured on 1% glycerol were amended with 0.1% GlcNAc 30 min prior to harvesting. After a total growth period of 20 h, mycelium was harvested from all conditions and the mycelium was resuspended to reach 400 mg·mL^−1^ in cold 0.2 m K_i_PO_4_‐buffer, pH 6.5. Aliquots of the suspensions were incubated in a water bath at 30 °C. Uptake was initiated by addition of 0.1 nmol of radioactively labeled sugars (either [^14^C]‐N‐acetylglucosamine: 55 μCi·μmol^−1^ or [^14^C]‐Glucose: 290 μCi·μmol^−1^). To determine competition of uptake of GlcNAc by other sugars, an excess (20 mm) of one of the indicated unlabeled sugars (GlcNAc, glucose, glycerol, fructose, xylose, lactose, and galactose) was added to selected samples. After 5 s, the uptake was terminated by addition of cold quenching‐buffer (containing a 1 m solution of the respective unlabeled sugar (GlcNAc or glucose) in 0.2 m K_i_PO_4_‐buffer, pH 6.5) and the mycelium was collected on glass microfiber filters (Whatman). Retained radioactivity was determined on a Wallac 1409 Liquid Scintillation Counter (Perkin Elmer) by placing the filters in scintillation vials containing 5 mL of MicroSint (Perkin Elmer). Dry weight of equal amounts of the mycelium was determined to calculate sugar uptake in nmol·mg^−1^ cell dry weight·min^−1^. At least two biological and two technical replicates were used in each experiment, and significance was estimated using student's *t*‐test.

### Bioinformatics and statistics

The protein sequence of NGS1 (jgi|Trire2|79 669) was aligned using ClustalW for structural modeling by applying default parameters and Blast algorithm applied to find the homology structure. The best Modeller model was chosen based on the QMEAN analysis, and steric and geometric quality of the model, which was evaluated by MolProbity. ESPript 3.0 online tool employed to depict the secondary structure by using RmNag (PDB.4zm6) as a template. The 3D structure was modeled by a Swiss model and visualization was done in VMD [74]. Sequence alignment for the C‐terminal GCN5 domain was performed using ClustalW followed by manual correction with sequences retrieved from Uniprot with the following accession numbers: Q29495 (AANAT) from *Ovis aries*, Q12341 (Hat1) from *S. cerevisiae*, Q03330 (Gcn5) from *S. cerevisiae*, G0RNA9 (NGS1) from *T. reesei*, A0A1D8PRM0 (Ngs1) from *C. albicans*, Q53396 (AAC3) from *Serratia marcescens*, and Q47764 (AAC6) from *Enterococcus faecium*. Sequence alignment of full‐length TrNGS1 and TrNGT1 was calculated using pairwise alignment with the online tool EMBOSS Needle from EMBL. For statistical analysis of RNA‐seq data, three biological replicates for each condition were analyzed using R (Version 4.2.1) with the bioconductor package and DESeq2 [72]. Fisher's exact test was performed for KOG enrichment analysis based on the KOG annotation provided in the JGI database https://mycocosm.jgi.doe.gov/cgi‐bin/kogBrowser?db=Trire2. For statistical analysis of growth assays and semi‐quantitative RT‐PCR results, at least two biological and three technical replicates were used. NGS1 orthologs for phylogenetic analyses were derived from clustering data with other GlcNAc catabolic genes from our previous work [13] and (Table S3). The phylogenetic tree analysis was performed in Mega11 [75], using neighbor joining as distance algorithmic method. Stability of clades was evaluated by 1000 bootstrap rearrangements.

## Conflicts of interest

The authors declare no conflict of interest.

## Author contributions

LK, VSS, SFU, and MO planned experiments. SFU and LK performed experiments. LK and SFU analyzed data and wrote the manuscript. VSS, BS, MO, EB, and VS contributed to data acquisition. All authors revised, corrected, and approved the manuscript.

## Supporting information


**Fig. S1.** Mutant phenotyping of *ngs1* and *ngt1*.
**Fig. S2.** Overlapping structure of TrNGS1 homodimer.
**Fig. S3.** Predicted surface structure of TrNGS1.
**Fig. S4.** Sequence alignment of *T. reesei* jgi|Trire2|79 669 (TrNGS1) with *C. albicans* CaNgs1 and *R. miehei* RmNag.
**Fig. S5.** RNA‐seq analysis of gene expression of *ngt1* and putative homologs.
**Fig. S6.** Gene expression analysis (semi‐quantitative RT‐PCR).
**Table S1.** RNA‐Seq data for wild‐type grown on GlcNAc and compared with control (WT glycerol).
**Table S2.** Fisher's exact test values for enriched KOG categories of DEGs in the WT on GlcNAc.
**Table S3.** Species and accession numbers for TrNGS1 orthologs.
**Table S4.** Strains used in this study.
**Table S5.** RNA‐Seq data for Δ*ngs1* (jgi|Trire2|79 669) grown on GlcNAc and compared with control (WT grown on GlcNAc).
**Table S6.** RNA‐Seq data for Δ*ron1* (jgi|Trire2|79 673) grown on GlcNAc and compared with control (WT grown on GlcNAc).
**Table S7.** Protein sequence similarity (%) of TrNGT1 and orthologues with other fungal NGTs.
**Table S8.** Primers for construction of and verification of overexpression strains.
**Table S9.** Primers used for RT‐PCR.
**Table S10.** Full list of RNA‐Seq data for wild‐type grown on GlcNAc and compared with control (WT glycerol).
**Table S11.** Full list of RNA‐Seq data for Δ*ngs1* (jgi|Trire2|79 669) grown on GlcNAc and compared with control (WT grown on GlcNAc).
**Table S12.** Full list of RNA‐Seq data for Δ*ron1* (jgi|Trire2|79 673) grown on GlcNAc and compared with control (WT grown on GlcNAc).

## Data Availability

The data that support the findings of this study are available in the supplementary material of this article.
