# Supplementary material for: N‐acetylglucosamine sensing in the filamentous soil fungus Trichoderma reesei
Source: FEBS J. 2025 Feb 15;292(12):3072–90. doi: 10.1111/febs.70015 (PMC12176254; doi:10.1111/febs.70015)
Supplement: Supplementary file 1 — Fig. S1. Mutant phenotyping of ngs1 and ngt1. Fig. S2. Overlapping structure of TrNGS1 homodimer. Fig. S3. Predicted surface structure of TrNGS1. Fig. S4. Sequence alignment of T. reesei jgi|Trire2|79 669 (TrNGS1) with C. albicans CaNgs1 and R. miehei RmNag. Fig. S5. RNA‐seq analysis of gene expression of ngt1 and putative homologs. Fig. S6. Gene expression analysis (semi‐quantitative RT‐PCR). Table S1. RNA‐Seq data for wild‐type grown on GlcNAc and compared with control (WT glycerol). Table S2. Fisher's exact test values for enriched KOG categories of DEGs in the WT on GlcNAc. Table S3. Species and accession numbers for TrNGS1 orthologs. Table S4. Strains used in this study. Table S5. RNA‐Seq data for Δngs1 (jgi|Trire2|79 669) grown on GlcNAc and compared with control (WT grown on GlcNAc). Table S6. RNA‐Seq data for Δron1 (jgi|Trire2|79 673) grown on GlcNAc and compared with control (WT grown on GlcNAc). Table S7. Protein sequence similarity (%) of TrNGT1 and orthologues with other fungal NGTs. Table S8. Primers for construction of and verification of overexpression strains. Table S9. Primers used for RT‐PCR. Table S10. Full list of RNA‐Seq data for wild‐type grown on GlcNAc and compared with control (WT glycerol). Table S11. Full list of RNA‐Seq data for Δngs1 (jgi|Trire2|79 669) grown on GlcNAc and compared with control (WT grown on GlcNAc). Table S12. Full list of RNA‐Seq data for Δron1 (jgi|Trire2|79 673) grown on GlcNAc and compared with control (WT grown on GlcNAc). [file FEBS-292-3072-s001.pdf]

N-acetylglucosamine sensing in the filamentous soil fungus *Trichoderma reesei*

Sadia Fida Ullah[1], Mislav Oreb[2], Eckhard Boles[2], Vaibhav Srivastava[1], Verena Seidl-Seiboth[3,4], Bernhard Seiboth[3], and Lisa Kappel[3,5]

Affiliations:

[1] Division of Glycoscience, Department of Chemistry, KTH Royal Institute of Technology, AlbaNova University Centre, 10691 Stockholm, Sweden

[2] Institute of Molecular Biosciences, Faculty of Biological Sciences, Goethe University, Max-von-Laue Straße 9, 60438 Frankfurt, Germany

[3] Research Division Biochemical Technology, Institute of Chemical, Environmental and Bioscience Engineering, TU Wien, 1060 Vienna, Austria

[4] current address: Institute of Science and Technology, IST – Austria, 3400 Klosterneuburg, Austria

[5] current address: Division of Glycoscience, Department of Chemistry, KTH Royal Institute of Technology, AlbaNova University Centre, 10691 Stockholm, Sweden

Corresponding author: Lisa Kappel, email: lkappel@kth.se

Supplementary figures

Figure S1: Mutant phenotyping of *ngs1* and *ngt1* ……………………….……….......... page 2

Figure S2: Overlapping structure of TrNGS1 homodimer……………………………….. page 3

Figure S3: Predicted surface structure of TrNGS1……………………………………….. page 4

Figure S4: Sequence alignment of *T. reesei* jgi|Trire2|79669 (TrNGS1) with *C. albicans* CaNgs1 and *R. miehei* RmNag……………………………………………………….............. page 5

Figure S5: RNAseq analysis of gene expression of *ngt1* and putative homologs…. page 7

Figure S6: Gene expression analysis (semi-quantitative RT-PCR)……………………. page 8

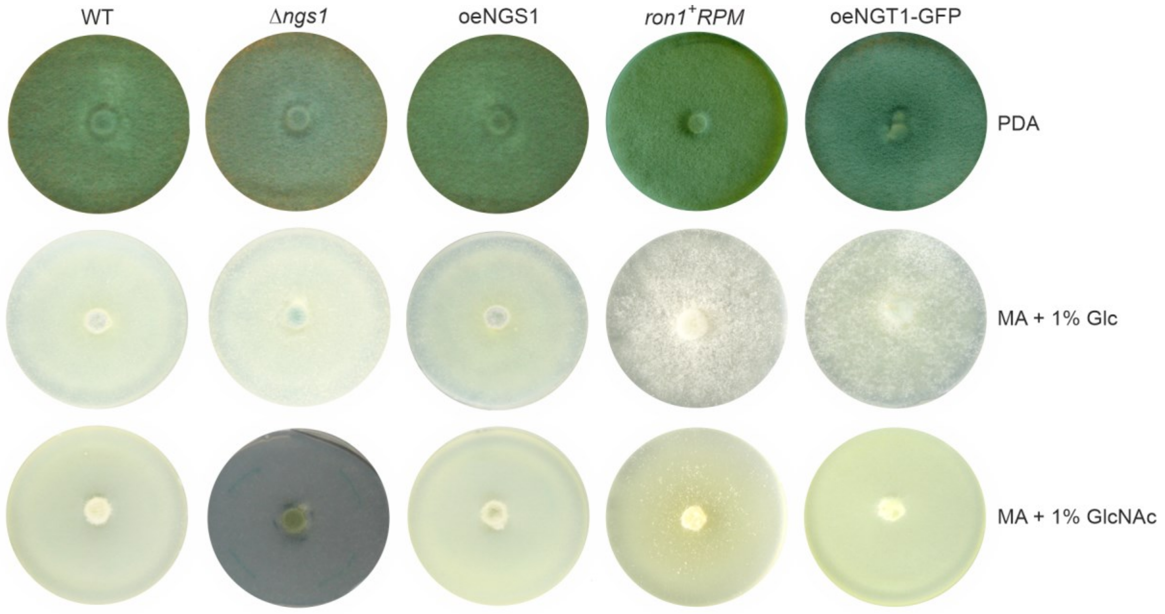

Figure S1: Mutant phenotyping of *ngs1* and *ngt1*. *T. reesei* WT strain, and the mutant strains Δ*ngs1*, oe-*ngs1*, *ron1**RPM and the oe-*ngt1*-GFP were grown for six days on PDA or on MA agar plates (1.5% w/v agar) containing either 1% GlcNAc or 1% glucose (glc) as carbon source.

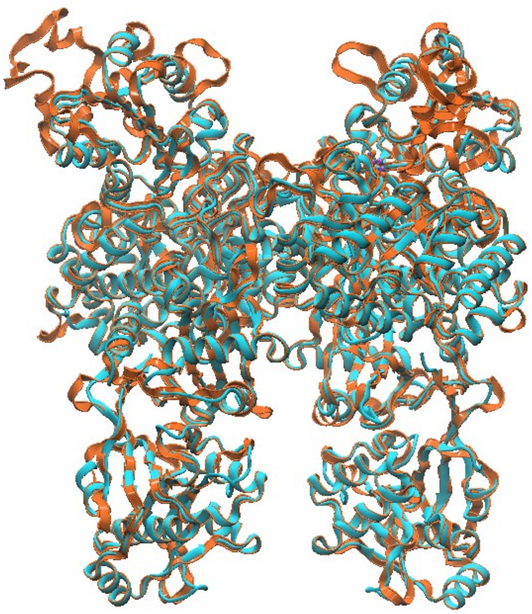

Figure S2: Overlapping structure of TrNGS1 (in red) and RmNag (in blue). A homodimer as proposed for RmNag is shown. Graphics were created using VMD [74].

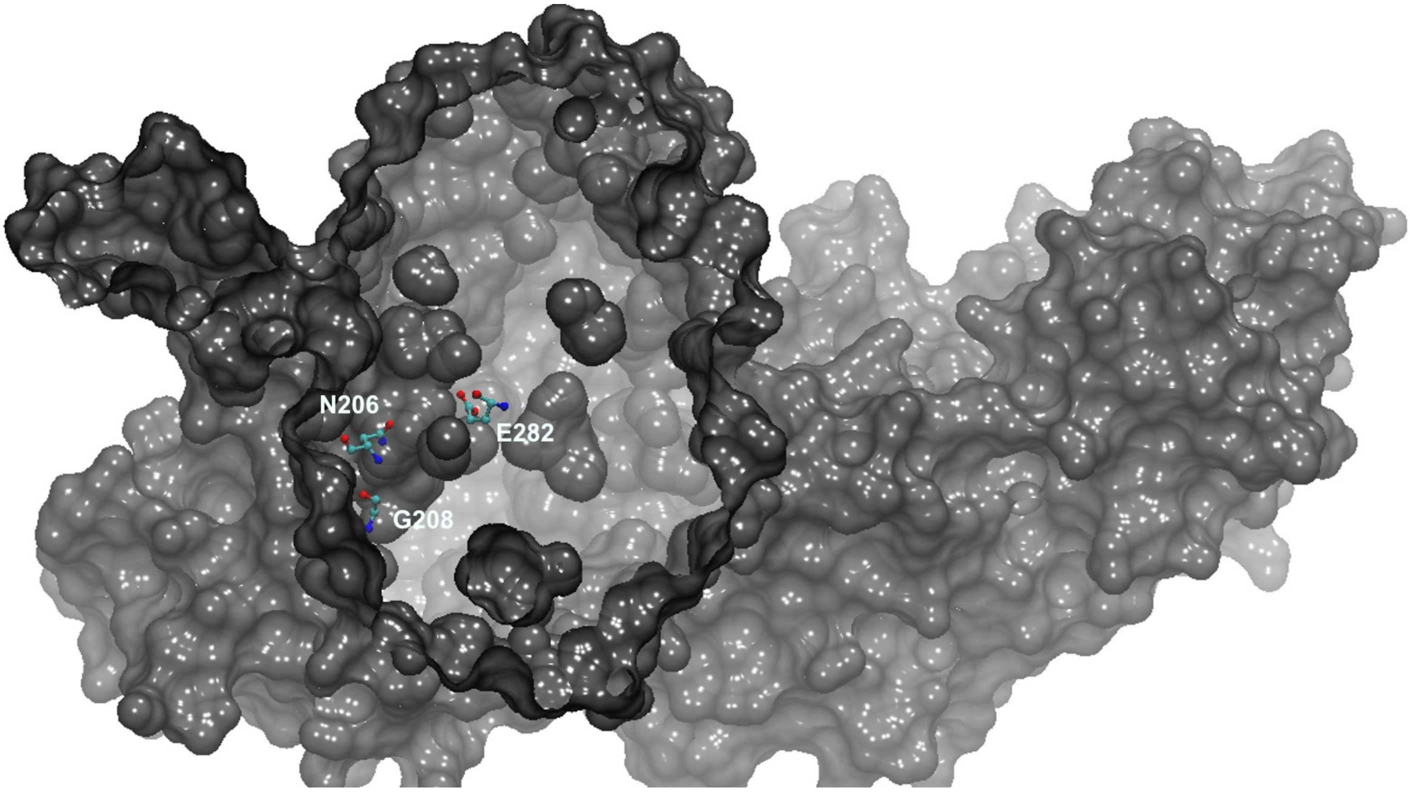

Figure S3: Predicted surface structure of TrNGS1, showing the active site groove and the variant catalytic residues depicted as colour sticks. Graphics were created using VMD [74].

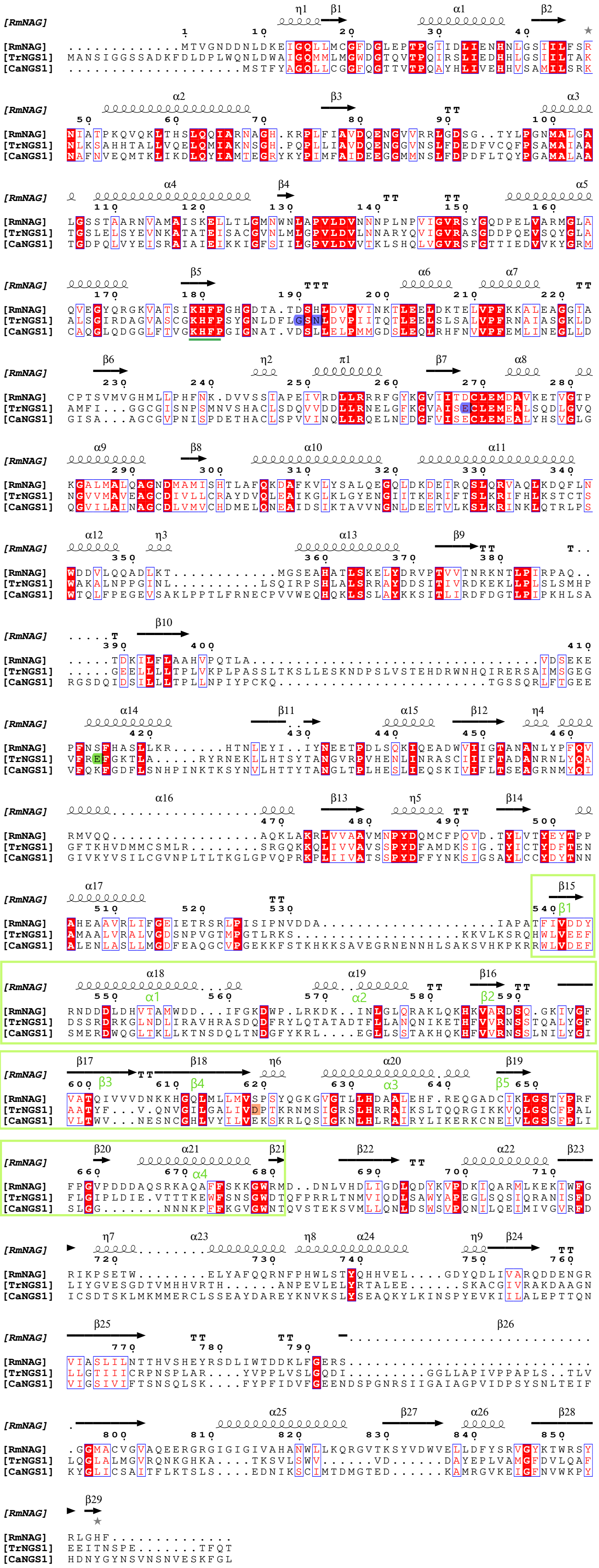
[RmNAG]
[TrNGS1]
[CaNGS1]

Figure S4. Sequence alignment of *T. reesei* jgi|Trire2|79669 (TrNGS1) with *C. albicans* CaNgs1 (A0A1D8PRM0) and *R. miehei* RmNag (V9M3A9). Residues colored in red font are conserved in one or the other species; while residues in a red box are identical in all three species used for this alignment. Residues known to interact with GlcNAc are underlined in green. The variant or non-catalytic residues in TrNGS1, which correspond to the catalytic activity of β-*N*-acetylglucosaminidase in RmNag, are highlighted in blue. The catalytic residue for β-glucosidase is highlighted in green and the proposed residue acting as base in the histone acetyltransferase is highlighted in orange. Conserved residues of Motif A, important for the GCN5 activity are highlighted with a green box. Sequence alignment was constructed using ESPript online tool using RmNag as a template crystalline structure (PBD: 4zm6).

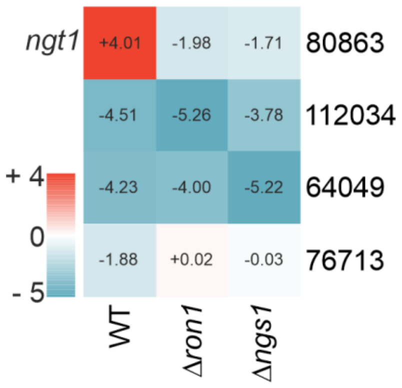

Figure S5: RNAseq analysis of gene expression of *ngt1* and putative homologs. log2-fold change from RNAseq expression analysis of closest homologs of TrNGS1 in *T. reesei* WT Δ*ron1, or* Δ*ngs1* in the presence of 1% GlcNAc compared to WT in the presence of 1% glycerol.

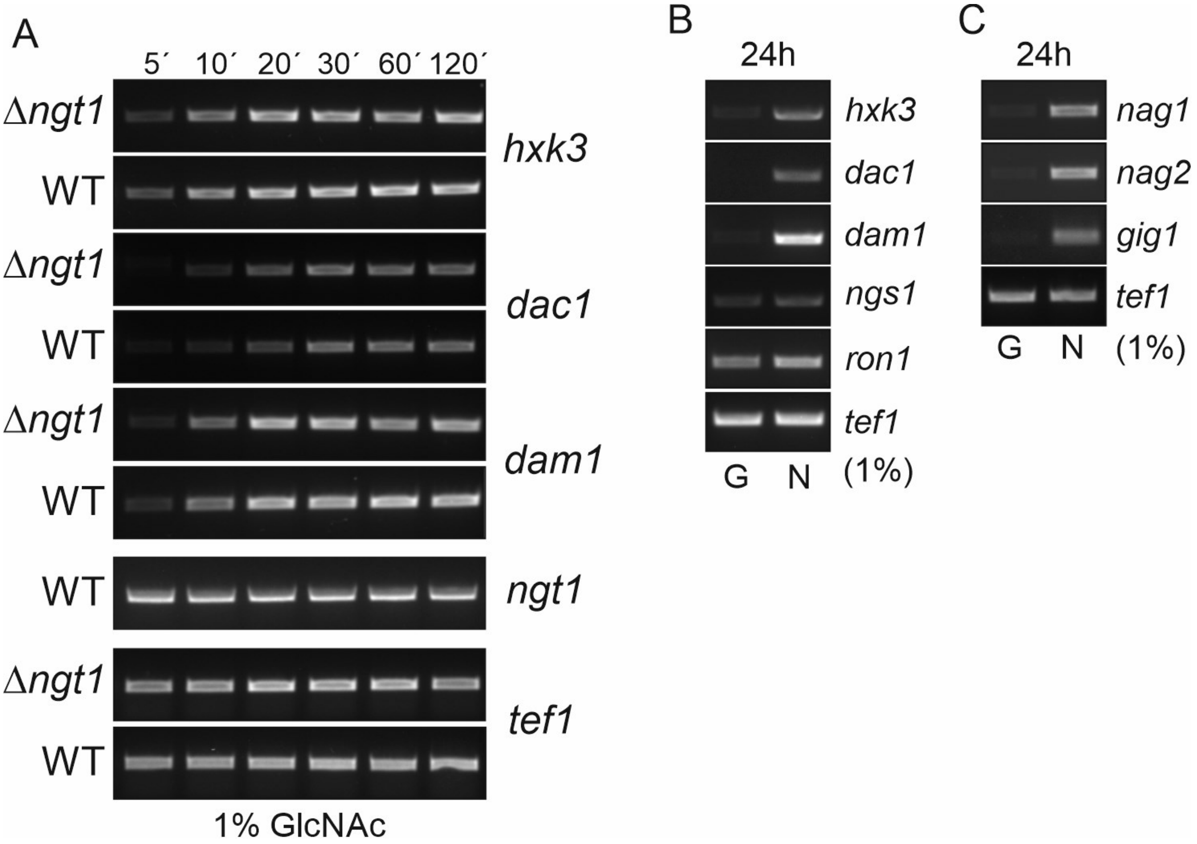

Figure S6: Gene expression analysis (semi-quantitative RT-PCR). (A) Gene expression analysis in the WT compared to the *ngt1* gene knock out strain. Expression of GlcNAc cluster genes after growth replacement from glycerol containing minimal medium to 1% GlcNAc was assessed after 5-120 min. *tef1* was used as reference gene. (B and C) Gene expression analysis (semi-quantitative RT-PCR) in the presence of 1% glucose (G) or 1% GlcNAc (N) after 24 hours. (B) Expression of GlcNAc cluster genes, and (C) expression of *nag1* and *nag2,* encoding N-acetylglucosaminidases, as well as *gig1,* encoding the ortholog for CaGig1 were assessed. *tef1* was used as reference gene. Representative gels from two independent experiments are shown.

Supplementary tables

Supplementary Table S1: RNA-Seq data for wild-type grown on GlcNAc and compared to control (WT glycerol). 9

Supplementary Table S2: Fisher´s exact test for enriched KOG-categories of DEGs in the WT on GlcNAc 11

Supplementary Table S3: Species and accession numbers for TrNGS1 orthologs 12

Supplementary Table S4: Strains used in this study 13

Supplementary Table S5: RNA-Seq data for Δ*ngs1* (jgi|Trire2|79669) grown on GlcNAc and compared to control (WT grown on GlcNAc). 14

Supplementary Table S6: RNA-Seq data for Δ*ron1* (jgi|Trire2|79673) grown on GlcNAc and compared to control (WT grown on GlcNAc). 16

Supplementary Table S7: Protein sequence similarity (%) of *T. reesei* TrNGT1 and orthologues with other fungal NGTs 18

Supplementary Table S8: Primers for construction and verification of overexpression strains ... 19

Supplementary Table S9: Primers used for RT-PCR 20

Supplementary Table S10: Full list of RNA-Seq data for wild-type grown on GlcNAc and compared to control (WT glycerol) 21

Supplementary Table S11: Full list of RNA-Seq data for Δ*ngs1* (jgi|Trire2|79669) grown on GlcNAc and compared to control (WT grown on GlcNAc) 25

Supplementary Table S12: Full list of RNA-Seq data for Δ*ron1* (jgi|Trire2|79673) grown on GlcNAc and compared to control (WT grown on GlcNAc) 30

Supplementary Table S1: RNA-Seq data for wild-type grown on GlcNAc and compared to control (WT glycerol).

Differentially expressed genes were defined by fold change ≥|1.0| and a adjusted p-value (padj) of < 0.05 found by DESeq2 (RStudio 2022.07.2+576)

The hightest 50 upregulated and lowest 50 downregulated genes are shown.

A full list of DEGs data is available in Suppl. Table S10.

	Identifier	log2FoldChange	padj	DEG	Gene name
1	TRIREDRAFT_49898	8.33	0.00E+00	UP	*dam1*
2	TRIREDRAFT_79671	7.91	0.00E+00	UP	*dac1*
3	TRIREDRAFT_102820	7.89	2.13E-90	UP	
4	TRIREDRAFT_44278	7.58	0.00E+00	UP	
5	TRIREDRAFT_79677	7.57	0.00E+00	UP	*hxk3*
6	TRIREDRAFT_103136	7.50	2.81E-08	UP	
7	TRIREDRAFT_111932	7.09	1.06E-16	UP	
8	TRIREDRAFT_108349	7.07	2.21E-07	UP	
9	TRIREDRAFT_112028	7.00	7.49E-07	UP	
10	TRIREDRAFT_81087	6.91	4.62E-31	UP	
11	TRIREDRAFT_76065	6.23	2.65E-286	UP	
12	TRIREDRAFT_61593	6.22	1.09E-03	UP	
13	TRIREDRAFT_54962	6.20	1.11E-124	UP	
14	TRIREDRAFT_110151	5.90	1.18E-04	UP	
15	TRIREDRAFT_105931	5.82	1.71E-105	UP	
16	TRIREDRAFT_59791	5.60	8.15E-05	UP	
17	TRIREDRAFT_120837	5.50	0.00E+00	UP	
18	TRIREDRAFT_77299	5.44	1.60E-129	UP	
19	TRIREDRAFT_46794	5.38	0.00E+00	UP	
20	TRIREDRAFT_61995	5.27	2.89E-03	UP	
21	TRIREDRAFT_54819	5.23	5.28E-284	UP	*gig1*
22	TRIREDRAFT_69883	5.22	6.63E-14	UP	
23	TRIREDRAFT_121164	5.06	8.86E-239	UP	
24	TRIREDRAFT_123649	5.06	8.51E-10	UP	
25	TRIREDRAFT_121486	4.97	2.13E-18	UP	
26	TRIREDRAFT_72137	4.96	7.49E-52	UP	
27	TRIREDRAFT_120176	4.89	1.55E-211	UP	
28	TRIREDRAFT_54761	4.87	9.72E-03	UP	
29	TRIREDRAFT_21725	4.84	1.63E-272	UP	*nag1*
30	TRIREDRAFT_111915	4.75	4.82E-14	UP	
31	TRIREDRAFT_48266	4.67	9.51E-04	UP	
32	TRIREDRAFT_111357	4.63	3.21E-46	UP	
33	TRIREDRAFT_53029	4.56	1.65E-02	UP	
34	TRIREDRAFT_5345	4.53	1.75E-83	UP	
35	TRIREDRAFT_51365	4.45	7.17E-298	UP	
36	TRIREDRAFT_123234	4.37	1.09E-07	UP	
37	TRIREDRAFT_112568	4.31	3.39E-02	UP	
38	TRIREDRAFT_108348	4.31	3.39E-02	UP	
39	TRIREDRAFT_69500	4.30	1.38E-04	UP	
40	TRIREDRAFT_42152	4.27	2.96E-07	UP	
41	TRIREDRAFT_122511	4.19	2.12E-217	UP	
42	TRIREDRAFT_103423	4.19	4.55E-02	UP	
43	TRIREDRAFT_105752	4.15	1.41E-02	UP	
44	TRIREDRAFT_69926	4.15	1.85E-22	UP	
45	TRIREDRAFT_5647	4.10	3.35E-30	UP	
46	TRIREDRAFT_105150	4.10	1.72E-02	UP	
47	TRIREDRAFT_121883	4.06	7.85E-27	UP	
48	TRIREDRAFT_81536	4.05	3.62E-03	UP	
49	TRIREDRAFT_108985	4.04	1.93E-02	UP	
50	TRIREDRAFT_80863	4.01	3.00E-216	UP	

Supplementary Table S1 (continued): RNA-Seq data for wild-type grown on GlcNAc and compared to control (WT glycerol).
Differentially expressed genes were defined by fold change ≥|1.0| and a adjusted p-value (padj) of < 0.05 found by DESeq2 (RStudio 2022.07.2+576)

	Identifier	log2FoldChange	padj	DEG	Gene name
2053	TRIREDRAFT_103179	-5.55	3.27E-103	DOWN	
2054	TRIREDRAFT_58366	-5.56	1.95E-04	DOWN	
2055	TRIREDRAFT_110711	-5.57	2.14E-09	DOWN	
2056	TRIREDRAFT_121350	-5.60	0.00E+00	DOWN	
2057	TRIREDRAFT_5330	-5.62	3.27E-05	DOWN	
2058	TRIREDRAFT_61605	-5.65	9.57E-05	DOWN	
2059	TRIREDRAFT_112491	-5.68	2.63E-05	DOWN	
2060	TRIREDRAFT_43312	-5.68	4.48E-113	DOWN	
2061	TRIREDRAFT_111750	-5.69	0.00E+00	DOWN	
2062	TRIREDRAFT_80086	-5.71	9.70E-76	DOWN	
2063	TRIREDRAFT_111251	-5.76	1.22E-86	DOWN	
2064	TRIREDRAFT_111838	-5.77	5.30E-112	DOWN	
2065	TRIREDRAFT_60419	-5.77	1.56E-05	DOWN	
2066	TRIREDRAFT_122079	-5.77	1.22E-275	DOWN	
2067	TRIREDRAFT_69901	-5.78	6.29E-05	DOWN	
2068	TRIREDRAFT_68812	-5.78	0.00E+00	DOWN	
2069	TRIREDRAFT_111082	-5.79	0.00E+00	DOWN	
2070	TRIREDRAFT_3488	-5.80	2.89E-42	DOWN	
2071	TRIREDRAFT_110127	-5.80	0.00E+00	DOWN	
2072	TRIREDRAFT_73818	-5.96	0.00E+00	DOWN	
2073	TRIREDRAFT_69650	-5.99	1.64E-213	DOWN	
2074	TRIREDRAFT_3094	-6.03	1.10E-54	DOWN	
2075	TRIREDRAFT_64869	-6.04	4.37E-06	DOWN	
2076	TRIREDRAFT_123086	-6.09	0.00E+00	DOWN	
2077	TRIREDRAFT_121136	-6.10	0.00E+00	DOWN	
2078	TRIREDRAFT_3267	-6.15	8.68E-38	DOWN	
2079	TRIREDRAFT_22331	-6.20	2.82E-220	DOWN	
2080	TRIREDRAFT_59876	-6.24	2.45E-41	DOWN	
2081	TRIREDRAFT_110457	-6.26	1.11E-103	DOWN	
2082	TRIREDRAFT_69692	-6.28	3.99E-12	DOWN	
2083	TRIREDRAFT_65039	-6.30	1.59E-107	DOWN	
2084	TRIREDRAFT_69696	-6.37	9.78E-07	DOWN	
2085	TRIREDRAFT_106660	-6.46	5.96E-07	DOWN	
2086	TRIREDRAFT_73101	-6.55	3.93E-07	DOWN	
2087	TRIREDRAFT_105269	-6.62	3.02E-07	DOWN	
2088	TRIREDRAFT_110740	-6.79	1.09E-07	DOWN	
2089	TRIREDRAFT_70365	-6.81	2.51E-63	DOWN	
2090	TRIREDRAFT_69834	-6.83	0.00E+00	DOWN	
2091	TRIREDRAFT_70934	-6.98	2.21E-07	DOWN	
2092	TRIREDRAFT_120923	-7.03	1.91E-60	DOWN	
2093	TRIREDRAFT_77552	-7.31	8.22E-99	DOWN	
2094	TRIREDRAFT_107881	-7.58	0.00E+00	DOWN	
2095	TRIREDRAFT_111122	-7.93	7.28E-100	DOWN	
2096	TRIREDRAFT_103061	-8.19	5.78E-11	DOWN	
2097	TRIREDRAFT_106928	-8.71	1.52E-117	DOWN	
2098	TRIREDRAFT_103062	-8.72	1.58E-45	DOWN	
2099	TRIREDRAFT_111121	-8.85	2.12E-95	DOWN	
2100	TRIREDRAFT_60489	-9.14	9.83E-14	DOWN	
2101	TRIREDRAFT_30465	-9.50	6.22E-37	DOWN	
2102	TRIREDRAFT_105882	-9.70	3.11E-15	DOWN	

Supplementary Table S2: Fisher´s exact test for enriched KOG-categories of DEGs in the WT on GlcNAc

#	KOG class	DEG			fisher´s exact test
		all	UP	DOWN	
**1**	**Carbohydrate transport and metabolism**	132	32	100	2,3E-12
**2**	**Amino acid transport and metabolism**	116	46	70	2,4E-12
**3**	**Secondary metabolites biosynthesis transport and catabolism**	103	31	72	1,3E-10
**4**	**Energy production and conversion**	99	23	76	6,5E-05
**5**	**Lipid transport and metabolism**	92	27	65	1,6E-04
**6**	**Inorganic ion transport and metabolism**	58	15	43	5,3E-04
**7**	**General function prediction only**	264	63	201	5,2E-03
8	Cell wall/membrane/envelope biogenesis	24	7	17	1,2E-01
9	Defense mechanisms	28	6	22	2,0E-01
10	Extracellular structures	14	3	11	5,7E-01
11	Coenzyme transport and metabolism	19	4	15	7,1E-01
12	Nucleotide transport and metabolism	18	7	11	7,8E-01
13	Signal transduction mechanisms	92	22	70	9,9E-01
14	Function unknown	44	16	28	1,0E+00
15	Transcription	40	18	22	1,0E+00
16	RNA processing and modification	40	19	21	1,0E+00
17	Translation ribosomal structure and biogenesis	19	8	11	1,0E+00
18	Intracellular trafficking secretion and vesicular transport	16	6	10	1,0E+00
19	Cell cycle control cell division chromosome partitioning	14	3	11	1,0E+00
20	Replication recombination and repair	12	3	9	1,0E+00
21	Cytoskeleton	11	2	9	1,0E+00
22	Chromatin structure and dynamics	11	3	8	1,0E+00
23	Nuclear structure	10	4	6	1,0E+00
24	Posttranslational modification protein turnover chaperones	7	2	5	1,0E+00

Bold letters and a dashed line separate the significantly enriched from the rest of the KOG-categories

Supplementary Table S3: Species and accession numbers for TrNGS1 orthologs

Species	strain	NCBI accession nr	jgi-identifiers*
Aspergillus niger	CBS 513.88	XP_001398206.2	
Beauveria bassiana		KGQ04902.1	
Botrytis.cinerea	BcDW1	EMR82271.1	
Coccidioides immitis A		XP_004446042.1	
Coccidioides immitis B		XP_001240395.2	
Dactylellina haptotyla	CBS 200.50	EPS41865.1	
Exophiala dermatitidis	NIH/UT8656	XP_009161604.1	
Fusarium solani		KAJ4234316.1	
Pyricularia oryzae		KAH8840788.1	
Metarhizium anisopliae	BRIP_53293	KJK81977.1	
Neurospora crassa	OR74A	XP_960361.3	
Candida albicans	SC5314	XP_718673.1	
Saitoella complicata	NRRL Y-17804	XP_019025139.1	
Trichoderma atroviride	IMI206040	EHK44614.1	jgi|Triat2|138324
Trichoderma reesei	**QM6a**	**XP_006966911.1**	**jgi|Trire2|79669**
Trichoderma virens	Gv29-8	XP_013958055.1	jgi|TriviGv29_8_2|76685
Yarrowia lipolytica	CLIB122	XP_504177.1	

* JGI identifiers are provided for *Trichoderma* spp. for consistency

Supplementary Table S4: Strains used in this study

Strain	Genotype	Reference
***T. reesei* QM9414**	*mat1-2*	Mandels and Andreotti (1978)
***T. reesei* QM9414 Δtku70**	*Δtku70::pyr4 mat 1-2*	C. Ivanova et al., unpuplished data
T. reesei Δron1	*mat1-2 Δtku70 Δron1::hph*	Kappel et al., 2016
T. reesei Δngs1*	*mat1-2 Δtku70 Δnag3::hph*	Kappel et al., 2016
T. reesei Δngt1	*mat1-2 Δtku70 Δngt1::hph*	Kappel et al., 2016
T. reesei Δhxk3	*mat1-2 Δtku70 Δhxk3::hph*	Kappel et al., 2016
T. reesei Δdac1	*mat1-2 Δtku70 Δdac1::hph*	Kappel et al., 2016
T. reesei Δdam1	*mat1-2 Δtku70 Δdam1::hph*	Kappel et al., 2016
T. reesei Δcre1	*mat1-2 Δtku70 Δcre1::hph*	Portnoy et al., 2011
***T. reesei* oeNGT1-GFP**	*mat1-2 ptef1::ngt1-GFP::hph*	this study
***T. reesei* oeNGS1**	*mat1-2 ptef1::ngs1-HIS::hph*	this study

* note that *nag3* and mutants derived from *nag3* were renamed to *ngs1* in this manuscript due to new evidence for this gene to be homologous to Ca*NGS1*

Supplementary Table S5: RNA-Seq data for Δ*ngs1* (jgi|Trire2|79669) grown on GlcNAc and compared to control (WT grown on GlcNAc).
Differentially expressed genes were defined by fold change ≥|1.0| in the Δngs1 mutant strain and an adjusted p-value (padj) of < 0.05 found by DESeq2 (RStudio 2022.07.2+576)
The lowest 50 downregulated genes are shown.
A full list of DEGs data is available in Suppl. Table S11.

	Identifier	log2FoldChange	padj	DEG	Gene name
1	TRIREDRAFT_79669	-10.50	3.27E-88	DOWN	*ngs1*
2	TRIREDRAFT_79816	-8.89	2.45E-51	DOWN	
3	TRIREDRAFT_55374	-8.46	2.01E-22	DOWN	
4	TRIREDRAFT_111121	-8.42	6.19E-155	DOWN	
5	TRIREDRAFT_106660	-8.09	2.46E-10	DOWN	
6	TRIREDRAFT_77552	-7.46	2.22E-134	DOWN	
7	TRIREDRAFT_106928	-7.30	6.51E-224	DOWN	
8	TRIREDRAFT_73623	-7.29	5.81E-08	DOWN	
9	TRIREDRAFT_5330	-7.24	4.98E-08	DOWN	
10	TRIREDRAFT_105882	-6.93	9.10E-28	DOWN	
11	TRIREDRAFT_110740	-6.56	3.41E-09	DOWN	
12	TRIREDRAFT_107881	-6.52	0.00E+00	DOWN	
13	TRIREDRAFT_121350	-6.33	0.00E+00	DOWN	
14	TRIREDRAFT_111122	-6.30	0.00E+00	DOWN	
15	TRIREDRAFT_109239	-6.29	8.46E-55	DOWN	
16	TRIREDRAFT_58366	-6.22	2.14E-05	DOWN	
17	TRIREDRAFT_102499	-6.17	3.18E-18	DOWN	
18	TRIREDRAFT_103061	-6.16	1.07E-22	DOWN	
19	TRIREDRAFT_30465	-6.12	2.95E-143	DOWN	
20	TRIREDRAFT_111082	-6.10	0.00E+00	DOWN	
21	TRIREDRAFT_69650	-6.00	2.49E-251	DOWN	
22	TRIREDRAFT_123086	-5.94	2.60E-246	DOWN	
23	TRIREDRAFT_108018	-5.94	6.98E-61	DOWN	
24	TRIREDRAFT_111750	-5.83	1.86E-299	DOWN	
25	TRIREDRAFT_70365	-5.77	5.02E-108	DOWN	
26	TRIREDRAFT_121136	-5.71	8.19E-222	DOWN	
27	TRIREDRAFT_108784	-5.65	6.44E-28	DOWN	
28	TRIREDRAFT_73250	-5.57	2.13E-47	DOWN	
29	TRIREDRAFT_22331	-5.55	1.43E-235	DOWN	
30	TRIREDRAFT_124043	-5.54	7.23E-05	DOWN	
31	TRIREDRAFT_69904	-5.50	1.73E-96	DOWN	
32	TRIREDRAFT_121416	-5.48	4.88E-175	DOWN	
33	TRIREDRAFT_110127	-5.48	0.00E+00	DOWN	
34	TRIREDRAFT_122824	-5.44	3.23E-56	DOWN	
35	TRIREDRAFT_103062	-5.42	3.56E-113	DOWN	
36	TRIREDRAFT_107641	-5.41	7.21E-04	DOWN	
37	TRIREDRAFT_49274	-5.39	8.38E-163	DOWN	
38	TRIREDRAFT_52315	-5.37	1.02E-185	DOWN	
39	TRIREDRAFT_111239	-5.35	3.71E-08	DOWN	
40	TRIREDRAFT_50793	-5.33	6.66E-43	DOWN	
41	TRIREDRAFT_64049	-5.22	1.72E-45	DOWN	
42	TRIREDRAFT_82041	-5.21	2.92E-99	DOWN	
43	TRIREDRAFT_80086	-5.18	3.82E-114	DOWN	
44	TRIREDRAFT_81576	-5.18	0.00E+00	DOWN	
45	TRIREDRAFT_105156	-5.17	4.52E-12	DOWN	
46	TRIREDRAFT_69555	-5.14	6.92E-04	DOWN	
47	TRIREDRAFT_68831	-5.09	2.04E-42	DOWN	
48	TRIREDRAFT_3094	-5.06	3.76E-88	DOWN	
49	TRIREDRAFT_54461	-5.04	1.75E-87	DOWN	
50	TRIREDRAFT_65410	-5.04	0.00E+00	DOWN	

Supplementary Table S5 (continued): RNA-Seq data for Δ*ngs1* (jgi|Trire2|79669) grown on GlcNAc and compared to control (WT grown on GlcNAc).

Differentially expressed genes were defined by fold change ≥|1.0| in the Δngs1 mutant strain and an adjusted p-value (padj) of < 0.05 found by DESeq2 (RStudio 2022.07.2+576).

The highest 50 upregulated genes are shown.

	Identifier	log2FoldChange	padj	DEG	Gene name
1	TRIREDRAFT_112568	10.42	2.5E-17	UP	
2	TRIREDRAFT_44278	10.28	0.0E+00	UP	
3	TRIREDRAFT_103136	9.80	2.8E-15	UP	
4	TRIREDRAFT_81087	9.25	3.5E-133	UP	
5	TRIREDRAFT_121441	9.06	1.1E-53	UP	
6	TRIREDRAFT_109361	8.97	3.1E-12	UP	
7	TRIREDRAFT_59689	8.83	2.8E-13	UP	
8	TRIREDRAFT_105279	8.23	8.0E-11	UP	
9	TRIREDRAFT_56289	8.08	2.1E-10	UP	
10	TRIREDRAFT_123234	7.99	1.8E-29	UP	
11	TRIREDRAFT_81275	7.92	8.3E-77	UP	
12	TRIREDRAFT_111915	7.85	2.6E-129	UP	
13	TRIREDRAFT_107867	7.36	2.0E-08	UP	
14	TRIREDRAFT_70800	7.28	4.8E-08	UP	
15	TRIREDRAFT_81536	7.16	4.5E-11	UP	
16	TRIREDRAFT_69736	7.13	6.4E-11	UP	
17	TRIREDRAFT_123550	7.10	1.5E-44	UP	
18	TRIREDRAFT_111932	6.88	1.1E-22	UP	
19	TRIREDRAFT_46794	6.87	0.0E+00	UP	
20	TRIREDRAFT_122495	6.82	7.6E-07	UP	
21	TRIREDRAFT_112128	6.72	4.7E-09	UP	
22	TRIREDRAFT_111138	6.71	1.1E-179	UP	
23	TRIREDRAFT_107869	6.65	1.9E-22	UP	
24	TRIREDRAFT_120837	6.63	0.0E+00	UP	
25	TRIREDRAFT_108349	6.62	1.4E-06	UP	
26	TRIREDRAFT_66819	6.62	5.1E-207	UP	
27	TRIREDRAFT_72632	6.60	3.7E-116	UP	
28	TRIREDRAFT_124051	6.53	7.5E-72	UP	
29	TRIREDRAFT_103451	6.48	0.0E+00	UP	
30	TRIREDRAFT_109235	6.42	7.7E-142	UP	
31	TRIREDRAFT_80659	6.37	8.2E-74	UP	
32	TRIREDRAFT_53029	6.35	9.1E-06	UP	
33	TRIREDRAFT_119790	6.30	8.8E-196	UP	
34	TRIREDRAFT_107868	6.27	1.1E-05	UP	
35	TRIREDRAFT_120176	6.27	0.0E+00	UP	
36	TRIREDRAFT_23415	6.27	0.0E+00	UP	
37	TRIREDRAFT_73897	6.25	2.4E-16	UP	
38	TRIREDRAFT_33387	6.24	1.4E-119	UP	
39	TRIREDRAFT_123978	6.22	0.0E+00	UP	
40	TRIREDRAFT_51365	6.16	0.0E+00	UP	
41	TRIREDRAFT_68427	6.13	4.2E-184	UP	
42	TRIREDRAFT_121164	6.04	0.0E+00	UP	
43	TRIREDRAFT_56646	6.01	2.0E-07	UP	
44	TRIREDRAFT_74563	5.96	4.0E-240	UP	
45	TRIREDRAFT_55881	5.92	8.1E-48	UP	
46	TRIREDRAFT_111890	5.83	2.9E-05	UP	
47	TRIREDRAFT_59843	5.80	1.8E-76	UP	
48	TRIREDRAFT_121495	5.76	2.5E-20	UP	
49	TRIREDRAFT_106537	5.74	4.8E-05	UP	
50	TRIREDRAFT_112568	10.42	2.5E-17	UP	

Supplementary Table S6: RNA-Seq data for Δron1 (jgi|Trire2|79673) grown on GlcNAc and compared to control (WT grown on GlcNAc).

Differentially expressed genes were defined by fold change ≥|1.0| in the Δngs1 mutant strain and an adjusted p-value (padj) of < 0.05 found by DESeq2 (RStudio 2022.07.2+576)

The lowest 50 downregulated genes are shown.

A full list of DEGs data is available in Suppl. Table S12.

	Identifier	log2FoldChange	padj	DEG	Gene name
1	TRIREDRAFT_79673	-12.03	2.88E-23	DOWN	*ron1*
2	TRIREDRAFT_55374	-9.00	2.07E-17	DOWN	
3	TRIREDRAFT_79816	-8.32	2.00E-62	DOWN	
4	TRIREDRAFT_106660	-8.03	3.44E-10	DOWN	
5	TRIREDRAFT_111121	-7.88	6.09E-177	DOWN	
6	TRIREDRAFT_106928	-7.46	6.28E-226	DOWN	
7	TRIREDRAFT_112491	-7.24	5.08E-08	DOWN	
8	TRIREDRAFT_77552	-7.21	2.93E-141	DOWN	
9	TRIREDRAFT_30465	-7.21	2.25E-123	DOWN	
10	TRIREDRAFT_5330	-7.18	6.69E-08	DOWN	
11	TRIREDRAFT_105882	-6.88	1.52E-27	DOWN	
12	TRIREDRAFT_103061	-6.60	2.29E-20	DOWN	
13	TRIREDRAFT_121416	-6.47	1.14E-27	DOWN	
14	TRIREDRAFT_102499	-6.45	2.02E-16	DOWN	
15	TRIREDRAFT_124043	-6.44	3.99E-06	DOWN	
16	TRIREDRAFT_107881	-6.43	0.00E+00	DOWN	
17	TRIREDRAFT_69901	-6.38	6.77E-06	DOWN	
18	TRIREDRAFT_69650	-6.25	5.65E-229	DOWN	
19	TRIREDRAFT_6103	-6.17	1.68E-05	DOWN	
20	TRIREDRAFT_58366	-6.16	2.50E-05	DOWN	
21	TRIREDRAFT_103062	-6.12	2.13E-137	DOWN	
22	TRIREDRAFT_58563	-6.11	2.30E-05	DOWN	
23	TRIREDRAFT_111750	-6.08	0.00E+00	DOWN	
24	TRIREDRAFT_69555	-6.05	6.29E-05	DOWN	
25	TRIREDRAFT_104260	-6.03	3.65E-05	DOWN	
26	TRIREDRAFT_108018	-6.02	1.09E-58	DOWN	
27	TRIREDRAFT_111122	-6.01	1.93E-242	DOWN	
28	TRIREDRAFT_111082	-6.01	2.20E-294	DOWN	
29	TRIREDRAFT_82041	-5.94	1.19E-90	DOWN	
30	TRIREDRAFT_110740	-5.94	1.61E-10	DOWN	
31	TRIREDRAFT_123086	-5.94	0.00E+00	DOWN	
32	TRIREDRAFT_122820	-5.85	7.93E-05	DOWN	
33	TRIREDRAFT_109239	-5.80	2.91E-59	DOWN	
34	TRIREDRAFT_73250	-5.77	8.21E-45	DOWN	
35	TRIREDRAFT_121136	-5.77	1.41E-57	DOWN	
36	TRIREDRAFT_52315	-5.74	1.45E-68	DOWN	
37	TRIREDRAFT_70365	-5.73	2.96E-109	DOWN	
38	TRIREDRAFT_121350	-5.70	0.00E+00	DOWN	
39	TRIREDRAFT_122824	-5.51	1.21E-54	DOWN	
40	TRIREDRAFT_49274	-5.43	1.24E-158	DOWN	
41	TRIREDRAFT_69904	-5.36	5.04E-96	DOWN	
42	TRIREDRAFT_68371	-5.36	7.91E-53	DOWN	
43	TRIREDRAFT_22331	-5.34	7.16E-244	DOWN	
44	TRIREDRAFT_112034	-5.26	7.04E-19	DOWN	
45	TRIREDRAFT_50793	-5.23	1.22E-41	DOWN	
46	TRIREDRAFT_78072	-5.19	1.51E-05	DOWN	
47	TRIREDRAFT_122795	-5.18	1.47E-162	DOWN	
48	TRIREDRAFT_69696	-5.09	1.54E-09	DOWN	
49	TRIREDRAFT_81576	-5.04	0.00E+00	DOWN	
50	TRIREDRAFT_3094	-5.02	1.39E-87	DOWN	

Supplementary Table S6 (continued): RNA-Seq data for Δron1 (jgi|Trire2|79673) grown on GlcNAc and compared to control (WT grown on GlcNAc).
Differentially expressed genes were defined by fold change ≥|1.0| in the Δngs1 mutant strain and an adjusted p-value (padj) of < 0.05 found by DESeq2 (RStudio 2022.07.2+576).
The highest 50 upregulated genes are shown.

	Identifier	log2FoldChange	padj	DEG	Gene name
1	TRIREDRAFT_112568	10.64	2.88E-18	UP	
2	TRIREDRAFT_44278	10.14	0.00E+00	UP	
3	TRIREDRAFT_103136	9.92	2.20E-15	UP	
4	TRIREDRAFT_121441	9.55	2.27E-58	UP	
5	TRIREDRAFT_81087	8.66	1.04E-107	UP	
6	TRIREDRAFT_59689	8.62	8.56E-22	UP	
7	TRIREDRAFT_111915	7.93	5.94E-157	UP	
8	TRIREDRAFT_81275	7.74	2.79E-54	UP	
9	TRIREDRAFT_56289	7.71	2.51E-09	UP	
10	TRIREDRAFT_123234	7.56	2.37E-27	UP	
11	TRIREDRAFT_109361	7.31	3.48E-08	UP	
12	TRIREDRAFT_105279	7.27	7.75E-08	UP	
13	TRIREDRAFT_108349	7.22	5.86E-08	UP	
14	TRIREDRAFT_69736	7.07	9.55E-11	UP	
15	TRIREDRAFT_81536	7.07	1.56E-10	UP	
16	TRIREDRAFT_111932	6.97	3.18E-22	UP	
17	TRIREDRAFT_122495	6.97	2.97E-07	UP	
18	TRIREDRAFT_123550	6.80	3.68E-39	UP	
19	TRIREDRAFT_119790	6.80	5.43E-250	UP	
20	TRIREDRAFT_46794	6.79	0.00E+00	UP	
21	TRIREDRAFT_111138	6.75	2.83E-152	UP	
22	TRIREDRAFT_120837	6.64	0.00E+00	UP	
23	TRIREDRAFT_33387	6.52	2.43E-55	UP	
24	TRIREDRAFT_80659	6.41	7.69E-17	UP	
25	TRIREDRAFT_109235	6.41	1.02E-129	UP	
26	TRIREDRAFT_107867	6.39	1.22E-05	UP	
27	TRIREDRAFT_124051	6.35	4.21E-32	UP	
28	TRIREDRAFT_120176	6.33	2.05E-262	UP	
29	TRIREDRAFT_70800	6.33	8.33E-06	UP	
30	TRIREDRAFT_72632	6.23	6.91E-105	UP	
31	TRIREDRAFT_51365	6.12	1.89E-298	UP	
32	TRIREDRAFT_66819	6.12	3.65E-173	UP	
33	TRIREDRAFT_123978	6.10	1.22E-267	UP	
34	TRIREDRAFT_103451	6.08	1.12E-299	UP	
35	TRIREDRAFT_121164	6.04	0.00E+00	UP	
36	TRIREDRAFT_107869	6.04	6.20E-19	UP	
37	TRIREDRAFT_73897	6.00	2.71E-40	UP	
38	TRIREDRAFT_66776	5.99	4.02E-05	UP	
39	TRIREDRAFT_23415	5.99	0.00E+00	UP	
40	TRIREDRAFT_56646	5.91	4.35E-07	UP	
41	TRIREDRAFT_74563	5.90	2.66E-283	UP	
42	TRIREDRAFT_106537	5.86	2.10E-05	UP	
43	TRIREDRAFT_59843	5.80	1.14E-85	UP	
44	TRIREDRAFT_62872	5.70	8.12E-190	UP	
45	TRIREDRAFT_111890	5.70	4.89E-05	UP	
46	TRIREDRAFT_68427	5.65	1.64E-152	UP	
47	TRIREDRAFT_53029	5.65	2.13E-04	UP	
48	TRIREDRAFT_121495	5.62	1.33E-17	UP	
49	TRIREDRAFT_53079	5.56	5.59E-60	UP	
50	TRIREDRAFT_107868	5.56	3.15E-04	UP	

Supplementary Table S7: Protein sequence similarity (%) of T. reesei TrNGT1 and orthologues with other fungal NGTs

	HcNgt1	HcNgt2	CaNgt1	TrNGT1	jgi|Trire2 |64049|	jgi|Trire2 |76713|	jgi|Trire2 |112034|
**HcNgt1**	100.0	32.30	39.5	57.8	35.0	39.7	42.2
**HcNgt2**	32.3	100.0	57.4	40.3	30.0	36.0	35.0
**CaNgt1**	39.5	57.4	100.0	65.8	37.6	47.0	43.5
**TrNGT1**	57.8	40.3	65.8	100.0	38.8	45.1	44.6
**jgi|Trire2|64049**	35.0	30.0	37.6	38.8	100.0	41.5	49.0
**jgi|Trire2|76713**	39.7	36.0	47.0	45.1	41.5	100.0	43.9
**jgi|Trire2|112034**	42.2	35.0	43.5	44.6	49.0	43.9	100.0

Protein sequences of HcNGT1 and its homolog HcNGT2 from *H. capsulatum* [8], CaNgt1 from *C. albicans* [23], and NGT1 from *T. reesei* (jgi|Trire2|80869) were compared to jgi|Trire2|64049, jgi|Trire2|76713, and gi|Trire2|112034 using pairwise comparison with EMBOSS.

Supplementary Table S8: Primers for construction and verification of overexpression strains

Primer name	Oligo sequence 5́-3́
**NGT1_TR_pRBVIIs**	ATCACACAAACCGTCGGATCCATGGCTGACGACGAAAAGCA
**NGT1_TR_pRBVIIa**	TTGCTCACCATGTTAATTAACACATCGGTGGTCTTGTTGG
**NGS1_TR_pPcdna1s**	CCAACAACTTCTCTCATCGATATGGCCAACTCCATCGGC
**NGS1_TR_pPcdna1a**	CTGCAGGTCGACATCGATACACGACAAGCCTCACAACC
**pRBVIIs**	TTAATTAACATGGTGAGCAAGGGC
**BampRBVIIa**	GGATCCGACGGTTTGTGTGATGTAGCG
**GFP_ctrl_a**	GCCGTCGTCCTTGAAGAAGA
**CTRL_cDNA1s**	ACCACCTCCCAAAACAAGCA

Supplementary Table S9: Primers used for RT-PCR

JGI - pID	Primer name	Gene	sequence 5´-3´	source
**21725**	Tr_NAG1_RTs	*nag1*	TCACCACATGGAACCTGACG	this study
	Tr_NAG1_RTa		GCATCCTCTCCCTGAACTCG	this study
**23346**	Tr_NAG2_RTs	*nag2*	TGTTTGACGATTTGCTGCCG	this study
	Tr_NAG2_RTa		TACACCGTCTGTAGGGTCGT	this study
**79669**	Tr_GH3_RTs	*ngs1*	ATCTGAGTGCCTGGTATGCG	Kappel et al., 2016
	Tr_GH3_RTa	*(nag3)*	CCACGAGCGGCTCATACG	Kappel et al., 2016
**79677**	Tr_HEX_RTs	*hxk3*	GGGCAAGATCTGGGAGACAT	Kappel et al., 2016
	Tr_HEX_RTa		TGGGATGAAGGGTACTTTTCCG	Kappel et al., 2016
**79671**	Tr_DAC_RTs	*dac1*	CATCACCTACTCGGTCGGC	Kappel et al., 2016
	Tr_DAC_RTa		CCTGAGGGCGTGAGGAATG	Kappel et al., 2016
**79674**	Tr_DAM_RTs	*dam1*	GCCTACGAGGACGCCATTAA	Kappel et al., 2016
	Tr_DAM_RTa		CCTTGGACGGCAGGATCTG	Kappel et al., 2016
**79673**	Tr_TF_RTs	*ron1*	GCGGGACAGTCTACTTGCTT	Kappel et al., 2016
	Tr_TF_RTa		TGAGCCTTGAATTCTGGGGG	Kappel et al., 2016
**80863**	Tr_NGTL_RTs	*ngt1*	ACAACCTGCTGTACTGGCTC	Kappel et al., 2016
	Tr_NGTL_RTa		CGGCGAACATGGTGTCGTA	Kappel et al., 2016
**54819**	Tr_GIG_RTs	*gig1*	CGCTACAAGGCCTTCACCTA	this study
	Tr_GIG_RTa		CATCGCCATTGGTGACCTCT	this study
**46958**	Tr_tef1_Fw	*tef1*	GTACTGGTGAGTTCGAGGCTG	Kappel et al., 2016
	Tr_tef1_Rev		GGGCTCGATGGAGTCGATG	Kappel et al., 2016

Supplementary Table S10: FULL list of DEGs from RNA-Seq data for wild-type grown on GlcNAc and compared to control (WT glycerol).

Differentially expressed genes (DEGs) were defined by fold change ≥|1.0| and a adjusted p-value (padj) of < 0.05 found by DESeq2 (RStudio 2022.07.2+576)

	Identifier (TRIREDRAFT)	log2fc	padj		Identifier (TRIREDRAFT)	log2fc	padj		Identifier (TRIREDRAFT)	log2fc	padj		Identifier (TRIREDRAFT)	log2fc	padj		Identifier (TRIREDRAFT)	log2fc	padj		Identifier (TRIREDRAFT)	log2fc	padj
1	49898	8,33	0,00E+00	101	112538	2,68	4,85E-08	201	69066	1,75	3,09E-06	301	123723	1,38	1,04E-44	401	22252	1,18	6,83E-28	501	106029	1,05	1,10E-08
2	79671	7,91	0,00E+00	102	107340	2,65	2,81E-149	202	48080	1,74	1,47E-40	302	109746	1,38	2,43E-24	402	79921	1,18	3,43E-05	502	29333	1,05	5,20E-15
3	102820	7,89	2,13E-90	103	112669	2,64	5,78E-29	203	64778	1,74	1,73E-36	303	56434	1,37	3,05E-52	403	105765	1,18	2,21E-04	503	77215	1,05	8,81E-10
4	44278	7,58	0,00E+00	104	76151	2,61	2,05E-02	204	23171	1,74	1,84E-02	304	121166	1,36	1,73E-34	404	106720	1,18	4,33E-07	504	21571	1,05	5,08E-14
5	79677	7,57	0,00E+00	105	122972	2,60	1,41E-42	205	124169	1,72	3,68E-27	305	106879	1,36	1,95E-08	405	61219	1,18	3,60E-20	505	77699	1,05	1,12E-10
6	103136	7,50	2,81E-08	106	79669	2,60	3,57E-128	206	29346	1,71	1,40E-04	306	80973	1,36	6,02E-16	406	66804	1,18	1,06E-16	506	81125	1,05	1,71E-28
7	111932	7,09	1,06E-16	107	106270	2,59	8,84E-25	207	64010	1,71	9,62E-50	307	109929	1,35	6,10E-25	407	64920	1,18	1,78E-14	507	111237	1,05	5,55E-07
8	108349	7,07	2,21E-07	108	69611	2,59	9,32E-28	208	55055	1,71	5,34E-34	308	59028	1,35	1,38E-04	408	111135	1,18	5,31E-05	508	80748	1,05	3,13E-18
9	112028	7,00	7,49E-07	109	54675	2,56	2,26E-149	209	108870	1,70	1,16E-05	309	32293	1,35	6,03E-04	409	111957	1,17	1,57E-02	509	30018	1,05	5,62E-16
10	81087	6,91	4,62E-31	110	122874	2,55	3,57E-32	210	108802	1,70	1,03E-09	310	22512	1,35	1,80E-18	410	104451	1,17	3,77E-11	510	57277	1,05	4,16E-15
11	76065	6,23	2,65E-286	111	76155	2,52	5,98E-141	211	78496	1,70	5,85E-56	311	121431	1,34	2,19E-13	411	42418	1,17	5,71E-08	511	52334	1,05	7,56E-18
12	61593	6,22	1,09E-03	112	50104	2,52	3,10E-04	212	119819	1,70	3,85E-53	312	57868	1,34	1,55E-11	412	73560	1,17	7,66E-18	512	122987	1,05	2,21E-19
13	54962	6,20	1,11E-124	113	122147	2,52	1,44E-26	213	79598	1,69	8,22E-21	313	74476	1,34	7,66E-38	413	60591	1,17	4,47E-12	513	122007	1,05	6,01E-08
14	110151	5,90	1,18E-04	114	54239	2,51	6,79E-50	214	110843	1,69	1,21E-03	314	73690	1,34	3,60E-28	414	123039	1,17	3,26E-25	514	122069	1,05	5,03E-11
15	105931	5,82	1,71E-105	115	81097	2,50	4,91E-62	215	119623	1,68	8,52E-65	315	46238	1,33	6,23E-32	415	105631	1,17	9,27E-21	515	44684	1,04	2,96E-15
16	59791	5,60	8,15E-05	116	78499	2,50	1,96E-94	216	46266	1,68	2,23E-19	316	106116	1,33	6,92E-04	416	78591	1,17	2,82E-27	516	106152	1,04	2,13E-02
17	120837	5,50	0,00E+00	117	111494	2,50	4,95E-02	217	58114	1,68	5,63E-37	317	67239	1,33	1,09E-18	417	106248	1,17	2,28E-17	517	76828	1,04	3,46E-16
18	77299	5,44	1,60E-129	118	58066	2,48	2,40E-03	218	122487	1,67	3,72E-45	318	63001	1,33	4,06E-33	418	79361	1,16	2,77E-15	518	53503	1,04	1,53E-03
19	46794	5,38	0,00E+00	119	3591	2,46	9,23E-73	219	21908	1,67	1,56E-57	319	122531	1,33	6,87E-22	419	67484	1,16	3,99E-22	519	110550	1,04	2,84E-11
20	61995	5,27	2,89E-03	120	123029	2,46	5,82E-51	220	78757	1,66	1,28E-49	320	66437	1,33	1,85E-13	420	68154	1,16	2,27E-15	520	61815	1,04	6,24E-15
21	54819	5,23	5,28E-284	121	70907	2,46	4,39E-23	221	70984	1,66	7,19E-20	321	56384	1,33	2,25E-17	421	104089	1,15	2,27E-03	521	108332	1,04	3,29E-20
22	69883	5,22	6,63E-14	122	120702	2,45	3,19E-26	222	64719	1,65	1,50E-24	322	60346	1,33	6,41E-20	422	102876	1,15	4,53E-03	522	49517	1,04	3,27E-15
23	121164	5,06	8,86E-239	123	63733	2,45	1,43E-51	223	69164	1,65	5,82E-04	323	45573	1,33	3,00E-18	423	74807	1,15	3,64E-13	523	66877	1,04	1,42E-11
24	123649	5,06	8,51E-10	124	121251	2,44	7,48E-68	224	106259	1,65	6,94E-07	324	120219	1,32	2,27E-23	424	108287	1,15	2,29E-17	524	121098	1,04	3,60E-25
25	121486	4,97	2,13E-18	125	103009	2,43	1,93E-02	225	75838	1,65	3,18E-20	325	106556	1,32	7,09E-04	425	34197	1,15	1,04E-10	525	71817	1,04	3,53E-02
26	72137	4,96	7,49E-52	126	68705	2,41	1,89E-75	226	4064	1,64	9,00E-36	326	122579	1,32	6,97E-09	426	82516	1,14	1,14E-19	526	49770	1,03	4,07E-17
27	120176	4,89	1,55E-211	127	120160	2,40	1,71E-53	227	82145	1,64	1,46E-22	327	21653	1,32	1,08E-28	427	57302	1,14	7,59E-15	527	59894	1,03	4,68E-13
28	54761	4,87	9,72E-03	128	81517	2,38	4,92E-61	228	103230	1,63	1,12E-63	328	57860	1,32	2,92E-24	428	45459	1,14	1,74E-18	528	2716	1,03	2,68E-08
29	21725	4,84	1,63E-272	129	62611	2,37	2,96E-97	229	123174	1,63	8,54E-62	329	108261	1,32	3,71E-13	429	106043	1,14	1,16E-03	529	40814	1,03	2,42E-07
30	111915	4,75	4,82E-14	130	119576	2,36	9,90E-51	230	65041	1,63	1,43E-26	330	66788	1,32	3,97E-06	430	57975	1,14	1,94E-08	530	38527	1,03	1,13E-04
31	48266	4,67	9,51E-04	131	70021	2,33	3,49E-03	231	103275	1,63	6,37E-06	331	80151	1,32	1,53E-16	431	54366	1,14	2,44E-09	531	105636	1,03	6,53E-25
32	111357	4,63	3,21E-46	132	109234	2,31	1,23E-69	232	62263	1,62	2,13E-16	332	105565	1,31	6,08E-12	432	108284	1,14	1,77E-19	532	22013	1,03	1,25E-15
33	53029	4,56	1,65E-02	133	104394	2,29	1,97E-03	233	47286	1,62	6,15E-29	333	54809	1,31	2,62E-24	433	61763	1,14	1,65E-24	533	51455	1,03	1,08E-27
34	5345	4,53	1,75E-83	134	81110	2,26	5,80E-144	234	121608	1,62	1,30E-18	334	56003	1,31	2,97E-11	434	109015	1,14	3,52E-24	534	42377	1,03	2,49E-14
35	51365	4,45	7,17E-298	135	120017	2,26	2,48E-60	235	111253	1,61	4,93E-14	335	59472	1,31	1,03E-10	435	74375	1,14	7,08E-24	535	120086	1,03	1,41E-22
36	123234	4,37	1,09E-07	136	62522	2,25	7,41E-10	236	22464	1,61	2,68E-29	336	80252	1,30	6,25E-18	436	1702	1,14	1,13E-22	536	66059	1,02	1,01E-13
37	112568	4,31	3,39E-02	137	56687	2,25	3,28E-02	237	77836	1,61	2,34E-17	337	70532	1,30	4,32E-31	437	109123	1,14	3,20E-26	537	70806	1,02	2,16E-05
38	108348	4,31	3,39E-02	138	52972	2,24	2,04E-39	238	75589	1,61	6,31E-30	338	57015	1,30	1,07E-03	438	2574	1,14	2,41E-35	538	104807	1,02	6,79E-20
39	69500	4,30	1,38E-04	139	120929	2,22	2,86E-12	239	51562	1,60	1,61E-62	339	121285	1,29	6,89E-15	439	65741	1,13	7,97E-11	539	107502	1,02	1,12E-05
40	42152	4,27	2,96E-07	140	107935	2,22	1,47E-03	240	120106	1,60	9,76E-58	340	68064	1,29	2,95E-27	440	122882	1,13	9,02E-36	540	103215	1,02	9,25E-08
41	122511	4,19	2,12E-217	141	67738	2,20	5,17E-36	241	120142	1,60	2,54E-34	341	67751	1,29	2,87E-14	441	74123	1,13	1,51E-31	541	80881	1,02	1,85E-22
42	103423	4,19	4,55E-02	142	67931	2,19	1,29E-56	242	72091	1,59	2,12E-35	342	107591	1,29	4,14E-08	442	23115	1,13	2,28E-32	542	104651	1,02	4,31E-04
43	105752	4,15	1,41E-02	143	75937	2,19	1,98E-17	243	108160	1,59	1,82E-09	343	65760	1,29	4,37E-22	443	71363	1,12	2,08E-14	543	108333	1,02	4,95E-10
44	69926	4,15	1,85E-22	144	77512	2,19	4,28E-86	244	73918	1,58	1,71E-42	344	63204	1,29	4,92E-21	444	74771	1,12	6,57E-20	544	4294	1,02	1,72E-08
45	5647	4,10	3,35E-30	145	108459	2,15	8,56E-10	245	39587	1,58	7,80E-04	345	56141	1,28	1,18E-18	445	104454	1,12	3,65E-22	545	55351	1,01	1,07E-26
46	105150	4,10	1,72E-02	146	65948	2,13	4,52E-42	246	73897	1,57	3,70E-02	346	59196	1,28	6,46E-11	446	39755	1,12	1,79E-16	546	69496	1,01	4,12E-04
47	121883	4,06	7,85E-27	147	119876	2,10	1,24E-27	247	58612	1,57	7,31E-75	347	68412	1,27	3,77E-22	447	121203	1,12	4,43E-22	547	107577	1,01	1,44E-09
48	81536	4,05	3,62E-03	148	111236	2,07	5,37E-12	248	56744	1,56	5,27E-27	348	107601	1,27	4,32E-22	448	123459	1,12	1,13E-08	548	65493	1,01	2,62E-05
49	108985	4,04	1,93E-02	149	105763	2,06	4,81E-32	249	72183	1,56	1,77E-27	349	119806	1,27	1,46E-23	449	80515	1,11	3,42E-21	549	109187	1,01	1,95E-13
50	80863	4,01	3,00E-216	150	81339	2,05	1,23E-43	250	122127	1,55	2,75E-23	350	80026	1,26	1,35E-04	450	54535	1,11	8,40E-22	550	23208	1,01	4,09E-09
51	112031	3,94	2,62E-74	151	81122	2,05	1,60E-55	251	123541	1,54	1,46E-09	351	67562	1,26	8,05E-21	451	120035	1,10	4,66E-14	551	78713	1,01	4,34E-11
52	124051	3,90	1,92E-16	152	70329	2,05	3,52E-03	252	62053	1,54	1,82E-05	352	61354	1,26	1,23E-08	452	59166	1,10	9,98E-21	552	112556	1,01	3,15E-13
53	62475	3,89	1,60E-16	153	67084	2,04	3,85E-06	253	123471	1,54	1,27E-33	353	123865	1,26	1,62E-26	453	68178	1,10	2,08E-11	553	22718	1,00	4,62E-22
54	74563	3,80	5,88E-78	154	122226	2,04	5,04E-53	254	22210	1,53	2,24E-11	354	21270	1,25	5,78E-35	454	122296	1,10	2,10E-33	554	56838	1,00	2,32E-10
55	69282	3,72	1,34E-10	155	119931	2,02	9,30E-74	255	104820	1,53	2,76E-24	355	108191	1,25	8,35E-10	455	39578	1,10	3,84E-02	555	79345	1,00	2,37E-14
56	124059	3,71	6,19E-89	156	55928	2,02	1,05E-07	256	21960	1,52	8,98E-59	356	55049	1,25	3,66E-09	456	40775	1,10	2,56E-06	556	27599	1,00	3,75E-17
57	122375	3,70	1,30E-02	157	2489	2,02	4,96E-06	257	22257	1,52	2,98E-04	357	82512	1,25	4,56E-17	457	60945	1,10	4,57E-09	557	50616	1,00	5,17E-07
58	121306	3,62	2,32E-154	158	67030	2,01	1,16E-02	258	49864	1,51	1,01E-59	358	110620	1,25	1,75E-41	458	22093	1,10	6,43E-19	558	61183	1,00	9,87E-18
59	70967	3,56	2,61E-02	159	122614	2,01	6,19E-44	259	107436	1,51	1,60E-16	359	66041	1,25	7,17E-45	459	79545	1,09	2,79E-24	559	122696	1,00	2,30E-19
60	119896	3,54	8,65E-182	160	70998	2,00	7,36E-18	260	80019	1,51	4,38E-26	360	79495	1,25	3,21E-25	460	79510	1,09	2,07E-26	560	43242	1,00	3,26E-07
61	34493	3,49	5,49E-125	161	68843	1,99	7,31E-59	261	111681	1,51	4,69E-20	361	123124	1,25	4,84E-36	461	121464	1,09	8,38E-17	561	62071	1,00	3,83E-14
62	108914	3,36	2,41E-274	162	51499	1,99	8,91E-101	262	22004	1,50	1,03E-31	362	69290	1,25	1,12E-03	462	60564	1,09	3,77E-17	562			
63	60635	3,35	4,88E-06	163	21412	1,99	1,27E-51	263	52055	1,50	5,73E-16	363	67377	1,24	1,06E-10	463	74020	1,09	5,04E-15	563			
64	55802	3,34	1,67E-133	164	61536	1,99	6,48E-03	264	58170	1,50	1,29E-35	364	66563	1,24	1,60E-17	464	52248	1,09	2,69E-17	564			
65	81014	3,34	2,34E-153	165	68990	1,97	2,75E-31	265	105617	1,50	2,38E-14	365	48788	1,24	1,04E-29	465	104511	1,09	5,32E-06	565			
66	74070	3,26	3,39E-231	166	5502	1,97	9,88E-03	266	33387	1,49	6,66E-04	366	122659	1,24	9,75E-36	466	63328	1,09	8,99E-03	566			
67	59388	3,25	2,29E-18	167	121315	1,95	1,15E-02	267	45523	1,49	2,20E-17	367	109040	1,23	1,89E-27	467	22667	1,09	4,59E-16	567			
68	70973	3,24	4,52E-26	168	102582	1,94	6,56E-42	268	121706	1,49	4,12E-28	368	102738	1,23	6,81E-11	468	72643	1,09	1,46E-11	568			
69	80340	3,24	1,77E-74	169	111442	1,93	1,75E-18	269	31134	1,49	2,95E-25	369	120370	1,23	1,71E-12	469	110363	1,09	1,79E-02	569			
70	54352	3,23	2,24E-07	170	81843	1,93	9,22E-15	270	30578	1,48	9,97E-03	370	3765	1,23	9,73E-03	470	47766	1,09	1,48E-26	570			
71	54226	3,19	2,86E-51	171	62271	1,92	7,98E-46	271	59723	1,48	2,99E-16	371	102960	1,23	1,75E-11	471	68869	1,08	5,52E-18	571			
72	58899	3,17	9,60E-199	172	74228	1,92	6,90E-67	272	75000	1,48	6,19E-16	372	103959	1,23	4,00E-05	472	76073	1,08	6,12E-17	572			
73	121396	3,15	1,18E-146	173	65229	1,92	1,44E-48	273	105643	1,48	1,39E-39	373	3891	1,23	2,64E-15	473	63503	1,08	5,56E-18	573			
74	68122	3,12	1,15E-14	174	122813	1,92	9,35E-61	274	119609	1,47	3,44E-46	374	112613	1,22	6,45E-03	474	82544	1,08	2,86E-18	574			
75	105483	3,08	1,24E-07	175	63526	1,91	9,34E-41	275	22560	1,47	8,72E-35	375	68254	1,22	7,61E-03	475	56470	1,08	3,82E-15	575			
76	23346	3,08	3,93E-109	176	107386	1,89	5,97E-21	276	123820	1,46	8,27E-18	376	119710	1,22	2,51E-18	476	81630	1,08	1,88E-19	576			
77	53079	3,07	1,05E-88	177	48747	1,89	2,42E-38	277	58613	1,46	3,56E-39	377	122644	1,22	2,53E-28	477	63180	1,08	1,60E-09	577			
78	68230	3,05	2,70E-11	178	79059	1,87	1,18E-81	278	29108	1,46	8,22E-17	378	58427	1,22	7,80E-33	478	53818	1,08	3,63E-08	578			
79	76075	3,00	1,39E-190	179	34985	1,86	1,11E-23	279	48852	1,46	1,62E-36	379	78886	1,22	1,52E-11	479	55029	1,08	2,06E-15	579			
80	120698	2,99	1,81E-120	180	108216	1,86	3,26E-19	280	49193	1,46	2,15E-30	380	122166	1,22	2,85E-34	480	3394	1,07	3,81E-19	580			
81	109122	2,97	5,22E-08	181	34183	1,86	2,60E-11	281	4430	1,46	1,31E-05	381	109404	1,22	1,18E-15	481	119556	1,07	1,58E-07	581			
82	123914	2,96	2,69E-15	182	78738	1,85	1,02E-51	282	106129	1,46	2,18E-06	382	28185	1,22	1,42E-02	482	122572	1,07	3,83E-19	582			
83	65162	2,96	8,41E-42	183	76995	1,84	1,14E-05	283	78765	1,46	1,98E-34	383	63217	1,22	1,07E-21	483	119933	1,07	8,48E-08	583			
84	121441	2,91	3,44E-09	184	57914	1,83	2,28E-48	284	65965	1,45	1,30E-30	384	123795	1,22	2,67E-21	484	121308	1,07	2,01E-10	584			
85	57643	2,91	5,73E-31	185	29993	1,81	1,29E-33	285	38080	1,45	2,19E-19	385	58381	1,22	1,87E-02	485	75764	1,07	1,09E-28	585			
86	80875	2,90	1,83E-181	186	111955	1,81	1,43E-07	286	103761	1,45	2,50E-10	386	81855	1,22	3,56E-15	486	81296	1,07	1,08E-16	586			
87	62693	2,90	3,13E-82	187	82146	1,81	1,93E-26	287	120912	1,45	2,13E-22	387	74913	1,22	1,98E-07	487	67079	1,07	2,52E-14	587			
88	70400	2,90	1,99E-03	188	102908	1,79	5,74E-31	288	111923	1,44	2,45E-02	388	3412	1,21	3,13E-29	488	124332	1,07	1,70E-30	588			
89	112372	2,89	3,09E-02	189	75027	1,79	2,04E-48	289	70172	1,43	1,61E-11	389	65646	1,21	1,73E-24	489	79034	1,07	1,39E-21	589			
90	68615	2,88	4,32E-85	190	70542	1,78	1,21E-37	290	102789	1,43	2,95E-04	390	56363	1,21	9,74E-32	490	75475	1,07	3,57E-18	590			
91	109235	2,85	1,40E-17	191	112018	1,78	1,20E-21	291	33763	1,43	2,93E-10	391	53091	1,20	5,49E-14	491	77989	1,07	2,34E-14	591			
92	123550	2,85	6,55E-09	192	65055	1,77	7,34E-32	292	119616	1,42	4,47E-42	392	121903	1,20	2,89E-27	492	121230	1,06	7,51E-17	592			
93	73536	2,81	1,17E-55	193	67472	1,76	7,37E-56	293	62059	1,41	8,37E-32	393	81803	1,20	3,74E-19	493	122909	1,06	2,16E-13	593			
94	44956	2,81	1,32E-104	194	63692	1,76	7,04E-11	294	76659	1,40	7,48E-14	394	78340	1,20	2,11E-06	494	75447	1,06	1,53E-22	594			
95	111250	2,79	2,30E-05	195	104551	1,76	1,38E-03	295	122287	1,39	3,26E-30	395	44868	1,20	2,23E-33	495	58815	1,06	2,39E-32	595			
96	23415	2,78	4,31E-107	196	72524	1,76	4,48E-05	296	110079	1,39	2,06E-06	396	106538	1,20	3,37E-04	496	102676	1,06	1,40E-22	596			
97	76822	2,78	5,52E-88	197	79835	1,76	1,01E-45	297	80268	1,39	2,50E-39	397	79405	1,19	8,13E-25	497	75534	1,06	3,77E-26	597			
98	103059	2,76	8,28E-03	198	104333	1,76	1,43E-15	298	107934	1,39	1,00E-50	398	49059	1,19	6,64E-27	498	21435	1,06	1,53E-19	598			
99	70933	2,71	3,48E-08	199	109323	1,75	2,13E-03	299	57322	1,38	2,30E-13	399	123911	1,19	1,91E-06	499	107402	1,05	3,23E-20	599			
100	31248	2,71	1,65E-169	200	108553	1,75	4,67E-22	300	123616	1,38	6,57E-21	400	122684	1,19	8,62E-27	500	102383	1,05	1,87E-12	600			

Supplementary Table S10 (continued): FULL list of DEGs from RNA-Seq data for wild-type grown on GlcNAc and compared to control (WT glycerol).

Differentially expressed genes (DEGs) were defined by fold change ≥|1.0| and a adjusted p-value (padj) of < 0.05 found by DESeq2 (RStudio 2022.07.2+576)

	Identifier (TRIREDRAFT)	log2fc	padj		Identifier (TRIREDRAFT)	log2fc	padj		Identifier (TRIREDRAFT)	log2fc	padj		Identifier (TRIREDRAFT)	log2fc	padj		Identifier (TRIREDRAFT)	log2fc	padj		Identifier (TRIREDRAFT)	log2fc	padj
1	54893	-1,00	2,70E-17	101	108683	-1,09	2,73E-02	201	106657	-1,20	1,69E-08	301	64916	-1,32	1,20E-13	401	4767	-1,42	2,81E-18	501	69771	-1,56	7,34E-17
2	77481	-1,00	5,22E-16	102	121349	-1,09	6,99E-32	202	107197	-1,20	3,25E-10	302	79238	-1,32	2,21E-36	402	103064	-1,42	5,26E-04	502	60374	-1,56	7,35E-04
3	123255	-1,00	6,02E-20	103	78576	-1,09	3,43E-14	203	106575	-1,20	4,07E-03	303	5403	-1,32	3,05E-03	403	105515	-1,43	2,72E-03	503	79129	-1,56	1,59E-46
4	119568	-1,00	1,54E-23	104	122010	-1,09	2,13E-13	204	122998	-1,20	4,77E-08	304	124293	-1,32	1,19E-14	404	82616	-1,43	1,32E-41	504	51415	-1,56	3,73E-28
5	71039	-1,00	1,59E-11	105	120044	-1,10	3,94E-25	205	103694	-1,20	6,17E-11	305	44747	-1,32	2,82E-29	405	76887	-1,43	3,10E-33	505	79606	-1,56	8,97E-34
6	30068	-1,01	4,11E-02	106	73961	-1,10	6,06E-21	206	66614	-1,20	7,77E-04	306	63914	-1,32	6,97E-14	406	119690	-1,43	3,26E-43	506	80879	-1,56	1,50E-49
7	79396	-1,01	1,70E-12	107	4952	-1,10	1,26E-02	207	48281	-1,20	6,20E-25	307	81423	-1,32	1,62E-36	407	60177	-1,43	4,79E-04	507	109905	-1,56	3,19E-22
8	79202	-1,01	4,57E-02	108	82351	-1,10	4,63E-34	208	80654	-1,20	1,66E-02	308	104067	-1,32	3,08E-02	408	110623	-1,43	7,61E-05	508	107554	-1,57	1,72E-08
9	48438	-1,01	5,93E-16	109	119803	-1,10	1,62E-26	209	60758	-1,21	4,94E-06	309	21595	-1,33	3,86E-05	409	27706	-1,43	1,71E-03	509	121702	-1,57	1,85E-02
10	76235	-1,01	6,23E-18	110	5863	-1,10	9,04E-10	210	54144	-1,21	1,11E-17	310	124016	-1,33	4,82E-16	410	21716	-1,43	2,45E-03	510	82403	-1,57	7,25E-28
11	55881	-1,01	1,13E-02	111	66023	-1,10	1,15E-05	211	107268	-1,21	6,89E-20	311	121089	-1,33	8,50E-28	411	64345	-1,43	1,40E-06	511	73621	-1,57	3,41E-07
12	22153	-1,01	3,42E-12	112	124141	-1,10	8,55E-17	212	123508	-1,21	2,25E-19	312	65483	-1,33	5,25E-27	412	104182	-1,44	5,06E-08	512	5249	-1,57	4,91E-28
13	62380	-1,01	1,84E-06	113	123951	-1,10	8,08E-20	213	78223	-1,21	2,24E-11	313	53989	-1,33	3,57E-09	413	3464	-1,44	4,83E-30	513	5319	-1,57	5,48E-16
14	121682	-1,01	1,57E-14	114	103852	-1,10	4,91E-16	214	104168	-1,21	9,80E-04	314	110894	-1,33	1,27E-02	414	69946	-1,44	7,65E-23	514	123509	-1,57	1,46E-12
15	82068	-1,01	4,52E-12	115	65411	-1,10	8,05E-09	215	63464	-1,21	1,02E-08	315	107187	-1,33	3,96E-39	415	77739	-1,44	7,49E-10	515	39637	-1,57	2,51E-18
16	54086	-1,02	1,05E-06	116	120600	-1,10	6,11E-21	216	67008	-1,21	1,42E-04	316	120503	-1,33	1,22E-11	416	110762	-1,44	6,88E-09	516	73631	-1,58	7,21E-11
17	121310	-1,02	2,96E-24	117	68608	-1,10	3,87E-17	217	111088	-1,21	9,52E-03	317	68022	-1,33	4,53E-22	417	44529	-1,44	9,61E-30	517	112490	-1,58	1,46E-06
18	23367	-1,02	5,56E-22	118	106064	-1,11	9,97E-08	218	122091	-1,21	6,48E-33	318	63397	-1,33	3,84E-07	418	67418	-1,44	2,40E-14	518	111450	-1,58	4,03E-11
19	48541	-1,02	1,27E-21	119	58162	-1,11	2,61E-19	219	60664	-1,21	1,22E-19	319	21406	-1,33	3,05E-17	419	31798	-1,44	4,94E-14	519	120985	-1,58	1,95E-07
20	68566	-1,02	4,02E-03	120	120988	-1,11	2,27E-18	220	62787	-1,21	2,84E-19	320	3605	-1,34	7,99E-30	420	4561	-1,45	4,03E-10	520	55566	-1,58	3,24E-03
21	122526	-1,02	1,43E-27	121	123510	-1,11	3,34E-09	221	70912	-1,21	1,54E-02	321	4009	-1,34	1,05E-31	421	71970	-1,45	9,81E-17	521	121154	-1,59	7,57E-34
22	5088	-1,02	1,71E-09	122	76018	-1,11	7,35E-04	222	103157	-1,22	1,97E-16	322	72611	-1,34	6,27E-15	422	22453	-1,45	3,17E-06	522	112578	-1,59	3,12E-03
23	104140	-1,02	5,08E-07	123	2902	-1,11	9,17E-03	223	47077	-1,22	2,27E-08	323	73516	-1,34	1,04E-14	423	22633	-1,46	7,30E-59	523	65141	-1,59	2,55E-06
24	70139	-1,02	2,55E-02	124	74278	-1,12	8,67E-06	224	102970	-1,22	1,00E-02	324	80091	-1,34	3,42E-06	424	104180	-1,46	5,69E-03	524	102904	-1,59	2,53E-03
25	77749	-1,02	2,28E-12	125	120120	-1,12	1,74E-11	225	106356	-1,22	5,21E-13	325	68455	-1,34	1,12E-21	425	37921	-1,46	1,69E-07	525	30166	-1,59	7,22E-05
26	124288	-1,02	1,19E-21	126	64672	-1,12	9,36E-06	226	124260	-1,22	1,76E-13	326	81756	-1,34	6,11E-36	426	110862	-1,46	1,58E-09	526	109687	-1,59	9,41E-09
27	3717	-1,02	4,81E-02	127	68842	-1,12	7,28E-03	227	71399	-1,22	1,32E-20	327	119947	-1,34	2,27E-16	427	111849	-1,46	1,77E-02	527	124177	-1,59	7,55E-14
28	76763	-1,02	7,02E-03	128	107011	-1,12	3,64E-07	228	71123	-1,22	1,36E-02	328	104606	-1,34	7,76E-23	428	79237	-1,46	7,39E-11	528	81700	-1,59	5,80E-50
29	105628	-1,02	2,36E-02	129	66311	-1,12	1,29E-03	229	62556	-1,22	6,29E-07	329	50894	-1,34	1,24E-02	429	109756	-1,46	5,53E-07	529	63378	-1,60	4,07E-45
30	106330	-1,02	5,71E-12	130	73511	-1,12	8,31E-21	230	67133	-1,22	3,75E-10	330	124001	-1,34	8,62E-25	430	71071	-1,46	1,10E-02	530	103158	-1,60	1,59E-03
31	51217	-1,02	2,30E-10	131	79300	-1,13	1,93E-27	231	67013	-1,22	8,78E-03	331	61476	-1,34	3,41E-08	431	110323	-1,46	1,40E-03	531	106895	-1,60	2,89E-11
32	109184	-1,02	1,39E-18	132	75175	-1,13	2,34E-12	232	45598	-1,23	2,56E-07	332	56863	-1,34	9,19E-16	432	81460	-1,47	7,59E-11	532	107938	-1,60	1,27E-16
33	27181	-1,03	2,41E-03	133	3363	-1,13	2,14E-07	233	112536	-1,23	8,73E-12	333	54616	-1,35	1,46E-13	433	120288	-1,47	1,21E-38	533	54511	-1,60	2,69E-19
34	80636	-1,03	7,87E-14	134	111202	-1,13	5,97E-16	234	124296	-1,23	6,13E-03	334	59833	-1,35	6,63E-15	434	103822	-1,47	6,86E-14	534	80621	-1,60	2,29E-23
35	121864	-1,03	6,70E-14	135	6037	-1,13	5,36E-27	235	78773	-1,23	2,65E-33	335	4843	-1,35	3,90E-16	435	106484	-1,47	1,83E-08	535	112267	-1,60	3,04E-04
36	74364	-1,03	1,34E-15	136	58389	-1,13	1,07E-12	236	54128	-1,23	5,86E-11	336	22271	-1,35	1,32E-08	436	66657	-1,47	3,98E-14	536	70808	-1,60	8,07E-07
37	79166	-1,03	1,74E-21	137	72383	-1,13	1,92E-25	237	1818	-1,23	2,64E-14	337	123978	-1,35	1,17E-06	437	65142	-1,47	4,60E-44	537	50131	-1,60	3,31E-44
38	80073	-1,03	1,96E-17	138	23200	-1,14	5,26E-22	238	120688	-1,24	8,08E-07	338	54036	-1,36	2,00E-02	438	111591	-1,48	3,89E-20	538	121474	-1,60	1,14E-32
39	107768	-1,04	8,63E-08	139	59014	-1,14	1,41E-15	239	65603	-1,24	4,41E-17	339	55559	-1,36	1,99E-14	439	27770	-1,48	2,64E-04	539	55631	-1,61	2,17E-33
40	36335	-1,04	1,31E-08	140	119986	-1,14	5,42E-25	240	110430	-1,24	1,15E-19	340	47710	-1,36	2,99E-28	440	119790	-1,48	1,96E-02	540	56830	-1,61	1,80E-04
41	4494	-1,04	4,04E-05	141	79694	-1,14	9,73E-11	241	61576	-1,24	6,72E-25	341	104077	-1,36	4,74E-02	441	39606	-1,48	3,18E-02	541	123084	-1,61	7,43E-12
42	120784	-1,04	2,40E-13	142	110692	-1,14	5,81E-16	242	70355	-1,24	9,89E-16	342	55634	-1,36	4,40E-04	442	23368	-1,48	5,57E-20	542	49928	-1,61	2,66E-11
43	82039	-1,04	4,97E-07	143	56399	-1,14	1,97E-18	243	60182	-1,24	2,38E-03	343	65746	-1,36	1,19E-19	443	21957	-1,48	3,85E-21	543	80685	-1,61	1,41E-11
44	77744	-1,04	9,68E-13	144	104842	-1,14	1,86E-04	244	105520	-1,24	2,82E-14	344	112146	-1,36	3,63E-11	444	111736	-1,48	4,30E-09	544	55584	-1,61	1,72E-41
45	123179	-1,04	1,60E-24	145	22030	-1,14	4,21E-27	245	59887	-1,24	9,71E-03	345	75311	-1,36	2,36E-22	445	119850	-1,48	4,60E-39	545	68086	-1,62	6,45E-31
46	120004	-1,04	2,20E-13	146	66544	-1,14	1,90E-07	246	76288	-1,24	1,75E-22	346	121894	-1,36	7,08E-24	446	60337	-1,48	3,94E-02	546	72012	-1,62	1,52E-34
47	58456	-1,04	4,18E-02	147	121579	-1,14	1,25E-19	247	36373	-1,24	2,29E-17	347	70994	-1,36	8,68E-03	447	110866	-1,49	1,79E-37	547	110415	-1,62	9,47E-22
48	73248	-1,04	7,16E-03	148	103771	-1,14	1,73E-03	248	62747	-1,25	1,82E-13	348	76249	-1,36	3,18E-29	448	105242	-1,49	1,52E-24	548	124157	-1,62	1,61E-41
49	106287	-1,04	2,38E-13	149	55599	-1,14	5,18E-23	249	52375	-1,25	5,74E-29	349	57098	-1,37	1,16E-23	449	120224	-1,49	1,18E-24	549	107347	-1,62	2,59E-02
50	70829	-1,05	1,29E-03	150	107973	-1,14	3,30E-07	250	42766	-1,25	5,88E-06	350	22481	-1,37	1,99E-30	450	59598	-1,49	2,61E-28	550	64066	-1,62	1,18E-15
51	55990	-1,05	2,01E-07	151	30214	-1,14	3,82E-18	251	54166	-1,25	7,62E-17	351	70191	-1,37	4,00E-31	451	123223	-1,49	1,08E-08	551	43286	-1,62	3,15E-48
52	108732	-1,05	6,54E-10	152	77770	-1,14	3,35E-03	252	67752	-1,25	8,80E-11	352	121785	-1,37	1,51E-03	452	3283	-1,49	3,47E-02	552	63416	-1,63	1,26E-06
53	21505	-1,05	1,89E-17	153	122963	-1,14	2,90E-18	253	65735	-1,25	2,26E-08	353	105223	-1,37	4,16E-03	453	4921	-1,49	3,78E-08	553	123278	-1,63	1,56E-02
54	104217	-1,05	8,94E-03	154	82037	-1,15	1,37E-21	254	109677	-1,25	8,01E-06	354	81649	-1,37	4,73E-30	454	67026	-1,49	4,77E-20	554	109793	-1,63	2,42E-03
55	79535	-1,06	5,25E-22	155	111893	-1,15	3,36E-07	255	119860	-1,26	6,30E-12	355	65433	-1,37	2,19E-35	455	66453	-1,50	1,57E-38	555	76136	-1,63	2,47E-44
56	82356	-1,06	1,27E-16	156	109790	-1,15	2,18E-09	256	3327	-1,26	1,52E-16	356	105518	-1,37	1,16E-22	456	109211	-1,50	5,78E-26	556	120872	-1,63	7,35E-36
57	54335	-1,06	2,73E-18	157	66103	-1,15	3,66E-14	257	56077	-1,26	3,55E-11	357	68427	-1,37	7,02E-06	457	79089	-1,50	4,60E-39	557	54525	-1,63	5,45E-21
58	59434	-1,06	3,01E-15	158	69816	-1,16	1,69E-04	258	50215	-1,26	2,40E-27	358	56206	-1,38	9,06E-23	458	22830	-1,50	1,62E-18	558	121294	-1,64	3,57E-41
59	53776	-1,06	1,25E-12	159	103366	-1,16	5,52E-04	259	43430	-1,26	1,94E-14	359	120339	-1,38	1,41E-19	459	61442	-1,50	7,31E-09	559	105775	-1,64	1,32E-19
60	65496	-1,06	1,64E-24	160	66966	-1,16	2,34E-33	260	121491	-1,26	2,15E-02	360	110414	-1,38	4,86E-11	460	120635	-1,50	5,74E-30	560	3718	-1,64	5,44E-05
61	79010	-1,06	5,19E-15	161	123562	-1,16	8,31E-17	261	123992	-1,27	2,36E-07	361	39942	-1,38	3,48E-15	461	69656	-1,50	2,28E-11	561	79737	-1,64	1,50E-25
62	2629	-1,06	3,76E-14	162	123702	-1,16	4,15E-33	262	104261	-1,27	1,68E-03	362	108201	-1,38	1,18E-06	462	59673	-1,50	6,98E-13	562	30668	-1,64	0,025457
63	55723	-1,06	5,58E-03	163	47127	-1,16	4,40E-24	263	57128	-1,27	1,48E-17	363	110133	-1,38	2,50E-06	463	61097	-1,51	2,68E-02	563	58699	-1,64	0,003314
64	72788	-1,06	2,54E-11	164	59095	-1,16	2,90E-05	264	5275	-1,27	9,74E-05	364	65949	-1,38	8,71E-21	464	102892	-1,51	5,43E-09	564	121868	-1,64	8,61E-31
65	67742	-1,07	2,40E-18	165	107616	-1,16	8,21E-10	265	79960	-1,27	2,21E-07	365	80142	-1,38	1,70E-03	465	104972	-1,51	1,20E-30	565	45503	-1,64	1,09E-20
66	5084	-1,07	7,26E-03	166	108014	-1,16	7,27E-03	266	102980	-1,27	5,97E-10	366	102778	-1,38	2,33E-02	466	69131	-1,51	4,14E-15	566	59151	-1,65	2,6E-08
67	121856	-1,07	4,15E-07	167	111523	-1,16	7,26E-09	267	112104	-1,27	2,88E-02	367	65522	-1,38	2,37E-24	467	120113	-1,51	6,19E-32	567	59940	-1,65	1,95E-24
68	37525	-1,07	7,92E-11	168	119981	-1,16	5,90E-28	268	120803	-1,27	6,62E-17	368	64274	-1,38	5,86E-06	468	123131	-1,52	1,81E-09	568	111567	-1,66	0,001104
69	70201	-1,07	3,05E-08	169	22064	-1,16	4,16E-12	269	110170	-1,28	5,13E-11	369	35777	-1,38	8,54E-31	469	59482	-1,52	1,80E-34	569	5889	-1,66	1E-08
70	79955	-1,07	2,75E-28	170	82074	-1,16	9,80E-17	270	78611	-1,28	2,99E-31	370	54694	-1,38	1,39E-08	470	109206	-1,52	1,78E-06	570	50039	-1,66	3,13E-52
71	82309	-1,07	4,51E-05	171	63558	-1,17	2,62E-09	271	59368	-1,28	4,64E-13	371	123241	-1,38	3,50E-09	471	110767	-1,52	7,30E-06	571	76641	-1,66	1,07E-21
72	36727	-1,07	9,80E-26	172	56218	-1,17	6,90E-14	272	36608	-1,28	3,10E-19	372	112330	-1,38	5,60E-13	472	65360	-1,52	4,60E-16	572	58550	-1,66	3,52E-27
73	120079	-1,07	1,18E-13	173	111476	-1,17	5,39E-06	273	121178	-1,28	8,86E-10	373	58607	-1,39	2,27E-23	473	77202	-1,52	1,97E-03	573	47603	-1,66	1,07E-33
74	2223	-1,07	7,74E-12	174	27649	-1,17	2,45E-30	274	5812	-1,28	2,82E-15	374	75444	-1,39	1,95E-05	474	110881	-1,52	4,45E-15	574	104104	-1,66	5,87E-06
75	79599	-1,07	4,03E-15	175	44362	-1,17	1,29E-18	275	104469	-1,29	1,08E-18	375	69468	-1,39	8,91E-04	475	27992	-1,52	8,80E-29	575	34252	-1,67	4,94E-10
76	59188	-1,07	1,38E-12	176	4875	-1,17	4,94E-09	276	123238	-1,29	2,48E-20	376	78373	-1,39	7,23E-28	476	104599	-1,52	1,43E-17	576	76949	-1,67	2,4E-50
77	109797	-1,08	1,74E-09	177	57558	-1,17	1,59E-03	277	61912	-1,29	6,37E-32	377	71092	-1,39	3,70E-33	477	53952	-1,52	1,52E-17	577	5776	-1,67	2,72E-38
78	104797	-1,08	1,46E-15	178	63568	-1,17	1,38E-07	278	62765	-1,29	2,41E-41	378	68950	-1,39	7,89E-03	478	52587	-1,53	2,75E-36	578	3645	-1,67	4,23E-30
79	108555	-1,08	4,32E-22	179	111758	-1,17	3,10E-04	279	78797	-1,29	5,56E-26	379	111345	-1,39	9,11E-04	479	48444	-1,53	4,02E-12	579	112247	-1,68	2,96E-06
80	120428	-1,08	2,03E-18	180	53267	-1,18	1,58E-04	280	106885	-1,29	1,95E-04	380	75794	-1,39	3,72E-54	480	69426	-1,53	1,25E-04	580	109585	-1,68	2,27E-30
81	103034	-1,08	1,81E-02	181	121240	-1,18	2,14E-20	281	70996	-1,29	5,58E-08	381	50212	-1,39	2,42E-38	481	71170	-1,53	9,27E-10	581	122299	-1,68	3,11E-52
82	21982	-1,08	3,65E-25	182	82452	-1,18	1,34E-37	282	54005	-1,29	2,80E-17	382	5612	-1,40	6,16E-17	482	81553	-1,53	1,07E-23	582	122242	-1,68	3,46E-52
83	57574	-1,08	3,49E-05	183	78688	-1,18	2,92E-23	283	124283	-1,29	5,64E-13	383	81115	-1,40	1,24E-18	483	104359	-1,54	7,75E-07	583	44175	-1,69	4,23E-18
84	110496	-1,08	7,07E-07	184	109946	-1,18	3,41E-02	284	108157	-1,29	1,78E-05	384	1777	-1,40	2,99E-30	484	45675	-1,54	6,84E-55	584	78387	-1,69	2,4E-35
85	69141	-1,08	3,45E-27	185	53373	-1,18	3,33E-05	285	109548	-1,30	1,07E-02	385	105275	-1,40	3,68E-07	485	111307	-1,54	1,23E-19	585	55105	-1,69	0,008347
86	75440	-1,08	2,03E-08	186	106642	-1,18	4,57E-14	286	22771	-1,30	9,22E-06	386	40374	-1,40	1,40E-02	486	112094	-1,54	1,16E-05	586	65921	-1,69	9,94E-09
87	53053	-1,08	2,53E-06	187	22341	-1,18	1,42E-28	287	82177	-1,30	5,34E-16	387	29619	-1,40	1,30E-02	487	65819	-1,54	7,65E-06	587	122941	-1,69	9,52E-40
88	122971	-1,08	5,74E-15	188	109337	-1,18	6,35E-14	288	5894	-1,30	5,88E-15	388	106118	-1,40	4,28E-23	488	22661	-1,54	4,33E-23	588	102966	-1,69	7,39E-09
89	47806	-1,08	1,80E-19	189	69695	-1,19	2,43E-04	289	106798	-1,30	4,39E-20	389	61965	-1,40	2,38E-46	489	35186	-1,54	2,87E-02	589	39221	-1,69	1,03E-20
90	122774	-1,08	2,55E-20	190	78681	-1,19	2,54E-23	290	75923	-1,30	4,85E-28	390	120114	-1,40	1,67E-13	490	76117	-1,55	1,08E-17	590	111515	-1,70	0,039945
91	22532	-1,09	4,81E-25	191	110744	-1,19	4,90E-09	291	120557	-1,30	2,38E-29	391	4240	-1,41	9,63E-04	491	49048	-1,55	5,16E-09	591	63413	-1,70	2,21E-17
92	107073	-1,09	9,23E-08	192	81591	-1,19	1,19E-20	292	4851	-1,31	1,98E-10	392	75036	-1,41	3,74E-34	492	108398	-1,55	1,91E-24	592	112134	-1,70	0,000114
93	57647	-1,09	1,21E-03	193	104741	-1,19	4,78E-07	293	42752	-1,31	3,45E-04	393	21876	-1,41	3,93E-04	493	62576	-1,55	3,24E-15	593	27259	-1,70	0,018658
94	48516	-1,09	5,22E-27	194	32849	-1,19	1,78E-05	294	79244	-1,31	5,76E-34	394	70840	-1,41	2,92E-32	494	47136	-1,55	6,78E-55	594	121427	-1,71	3,72E-45
95	21664	-1,09	3,34E-11	195	69384	-1,19	1,98E-14	295	4109	-1,31	3,81E-09	395	105224	-1,42	3,09E-11	495	81271	-1,55	3,52E-28	595	68574	-1,71	0,044855
96	107639	-1,09	7,63E-08	196	82227	-1,19	4,23E-11	296	112180	-1,31	7,48E-15	396	66795	-1,42	4,51E-04	496	123354	-1,55	9,62E-35	596	65117	-1,71	0,017956
97	81389	-1,09	4,54E-10	197	65583	-1,19	1,85E-03	297	67504	-1,31	1,50E-04	397	35768	-1,42	3,26E-12	497	109787	-1,55	1,78E-12	597	22510	-1,71	1,34E-32
98	111446	-1,09	2,04E-11	198	106108	-1,20	2,19E-13	298	122074	-1,31	5,55E-26	398	66786	-1,42	7,01E-04	498	60490	-1,55	4,55E-09	598	56211	-1,71	7,06E-12
99	61420	-1,09	3,43E-16	199	75368	-1,20	4,94E-19	299	4999	-1,31	2,14E-11	399	80056	-1,42	9,13E-10	499	109371	-1,56	2,97E-08	599	22535	-1,71	6,49E-25
100	106014	-1,09	3,93E-13	200	104075	-1,20	4,61E-12	300	63092	-1,31	5,76E-35	400	67907	-1,42	3,30E-21	500	66647	-1,56	3,86E-37	600	59698	-1,72	0,018144

Supplementary Table S10 (continued): FULL list of DEGs from RNA-Seq data for wild-type grown on GlcNAc and compared to control (WT glycerol).

Differentially expressed genes (DEGs) were defined by fold change ≥|1.0| and a adjusted p-value (padj) of < 0.05 found by DESeq2 (RStudio 2022.07.2+576)

	Identifier (TRIREDRAFT)	log2fc	padj		Identifier (TRIREDRAFT)	log2fc	padj		Identifier (TRIREDRAFT)	log2fc	padj		Identifier (TRIREDRAFT)	log2fc	padj		Identifier (TRIREDRAFT)	log2fc	padj		Identifier (TRIREDRAFT)	log2fc	padj
601	28787	-1,72	1,45E-32	701	121471	-1,90	4,79E-51	801	58574	-2,04	5,37E-05	901	62244	-2,30	6,01E-29	1001	120975	-2,51	1,36E-45	1101	106160	-2,81	7,34E-25
602	22426	-1,72	2,59E-25	702	75271	-1,90	5,46E-07	802	61830	-2,04	3,48E-02	902	109972	-2,30	2,13E-23	1002	102920	-2,52	3,79E-23	1102	60144	-2,81	3,23E-03
603	111395	-1,72	6,00E-18	703	36391	-1,90	3,21E-62	803	43161	-2,04	1,46E-05	903	124136	-2,31	3,84E-64	1003	105167	-2,52	3,35E-04	1103	120363	-2,81	3,55E-27
604	51907	-1,72	1,67E-20	704	123720	-1,91	1,62E-40	804	70949	-2,05	3,06E-09	904	107715	-2,31	2,61E-78	1004	107037	-2,52	5,65E-13	1104	112328	-2,82	1,57E-31
605	3196	-1,72	1,76E-54	705	103108	-1,91	4,45E-40	805	69375	-2,05	7,52E-06	905	106576	-2,31	1,03E-06	1005	107495	-2,53	5,59E-03	1105	121482	-2,83	1,33E-29
606	63152	-1,73	2,55E-20	706	105866	-1,91	1,39E-38	806	80980	-2,05	3,04E-58	906	59549	-2,31	5,34E-59	1006	69963	-2,53	1,02E-34	1106	77969	-2,84	2,32E-47
607	108405	-1,73	2,91E-24	707	68997	-1,91	4,07E-06	807	71029	-2,05	3,14E-76	907	62231	-2,31	1,20E-21	1007	120362	-2,53	1,88E-66	1107	112525	-2,84	8,15E-28
608	110768	-1,73	1,33E-17	708	105455	-1,91	1,73E-13	808	76690	-2,05	1,30E-22	908	53872	-2,31	3,10E-02	1008	65215	-2,53	2,87E-60	1108	49308	-2,85	1,12E-79
609	58602	-1,73	6,73E-09	709	103143	-1,91	2,63E-03	809	107962	-2,06	2,26E-04	909	104181	-2,32	3,01E-02	1009	45971	-2,53	6,35E-51	1109	122240	-2,86	3,93E-121
610	111382	-1,74	5,69E-35	710	56176	-1,91	1,27E-07	810	111138	-2,06	9,59E-03	910	106437	-2,32	1,08E-28	1010	46002	-2,54	1,74E-04	1110	76910	-2,86	3,52E-43
611	123120	-1,74	5,14E-20	711	105848	-1,91	8,57E-11	811	59624	-2,06	7,39E-21	911	54048	-2,32	1,48E-04	1011	69885	-2,54	1,70E-15	1111	21758	-2,86	3,00E-81
612	52446	-1,74	9,40E-25	712	2392	-1,91	1,58E-47	812	109716	-2,06	2,78E-61	912	103451	-2,32	7,68E-22	1012	64827	-2,54	1,12E-79	1112	123236	-2,86	1,80E-02
613	78833	-1,74	3,31E-17	713	82105	-1,91	1,24E-22	813	120415	-2,06	5,28E-42	913	3364	-2,32	8,58E-38	1013	60773	-2,54	5,06E-51	1113	6005	-2,87	1,93E-51
614	111304	-1,74	1,87E-07	714	107858	-1,92	1,04E-11	814	48482	-2,07	6,64E-50	914	108145	-2,32	6,99E-03	1014	57524	-2,54	2,04E-75	1114	66937	-2,87	1,35E-83
615	110830	-1,74	5,22E-04	715	103127	-1,92	2,61E-57	815	79813	-2,07	3,58E-51	915	61055	-2,33	1,45E-02	1015	120228	-2,55	3,12E-20	1115	120877	-2,87	8,92E-205
616	4480	-1,75	2,78E-32	716	74215	-1,93	1,54E-07	816	60328	-2,07	2,34E-25	916	112493	-2,33	1,11E-02	1016	111865	-2,55	1,97E-03	1116	58989	-2,88	3,50E-41
617	111110	-1,75	6,15E-07	717	67494	-1,93	1,40E-04	817	2499	-2,09	1,50E-12	917	120059	-2,33	6,31E-118	1017	65171	-2,55	4,02E-13	1117	70204	-2,88	8,22E-04
618	58244	-1,75	9,94E-13	718	105363	-1,93	9,93E-03	818	5337	-2,09	7,08E-09	918	119822	-2,33	3,66E-69	1018	76218	-2,55	5,57E-88	1118	4626	-2,88	6,88E-47
619	2583	-1,75	2,06E-19	719	3405	-1,93	7,49E-09	819	53526	-2,09	3,24E-06	919	65036	-2,33	3,63E-02	1019	54129	-2,56	2,02E-03	1119	59272	-2,88	3,10E-65
620	122569	-1,75	2,70E-22	720	75854	-1,93	4,36E-80	820	123205	-2,09	5,16E-09	920	65508	-2,33	8,66E-91	1020	109673	-2,56	9,04E-04	1120	103886	-2,88	2,23E-64
621	3889	-1,76	1,87E-35	721	65882	-1,93	9,52E-31	821	105330	-2,09	4,70E-03	921	121534	-2,33	4,74E-05	1021	2433	-2,56	7,69E-41	1121	107463	-2,89	6,96E-66
622	62463	-1,76	5,34E-22	722	108144	-1,93	1,08E-04	822	59843	-2,10	4,22E-02	922	119619	-2,34	2,77E-29	1022	69316	-2,57	4,47E-05	1122	80003	-2,89	3,20E-98
623	104968	-1,76	4,90E-28	723	3333	-1,93	1,46E-07	823	63653	-2,11	1,01E-04	923	69625	-2,34	3,89E-06	1023	122523	-2,57	1,95E-72	1123	121843	-2,89	1,18E-123
624	5890	-1,76	2,99E-04	724	108783	-1,94	3,71E-14	824	65975	-2,11	1,01E-31	924	110214	-2,34	3,85E-21	1024	41895	-2,58	9,79E-05	1124	105465	-2,90	2,14E-12
625	81303	-1,76	9,95E-66	725	82327	-1,94	6,75E-06	825	22678	-2,11	9,98E-63	925	104557	-2,34	1,61E-08	1025	64469	-2,58	2,64E-74	1125	45138	-2,91	4,04E-172
626	122416	-1,77	1,71E-18	726	74041	-1,94	3,98E-52	826	66562	-2,11	2,55E-45	926	22201	-2,34	1,66E-44	1026	81442	-2,59	4,98E-70	1126	65883	-2,91	7,40E-07
627	22284	-1,77	1,02E-52	727	5970	-1,94	1,87E-16	827	105869	-2,12	6,39E-03	927	69081	-2,35	2,45E-54	1027	104322	-2,59	2,42E-24	1127	5366	-2,91	4,34E-55
628	120231	-1,77	4,65E-20	728	81598	-1,94	1,19E-67	828	77000	-2,12	4,12E-48	928	70377	-2,35	1,97E-05	1028	111807	-2,59	6,46E-16	1128	50618	-2,92	2,81E-127
629	107835	-1,77	6,16E-04	729	42264	-1,94	4,11E-08	829	62165	-2,12	5,10E-16	929	62359	-2,35	3,79E-02	1029	105874	-2,60	1,24E-02	1129	22590	-2,92	1,25E-19
630	122582	-1,78	1,89E-11	730	105785	-1,94	2,75E-02	830	102766	-2,12	6,39E-37	930	121163	-2,35	6,31E-11	1030	69700	-2,60	7,30E-03	1130	70383	-2,92	1,03E-76
631	70373	-1,78	1,96E-13	731	61403	-1,94	2,69E-15	831	51378	-2,13	6,01E-67	931	81525	-2,35	1,51E-07	1031	61127	-2,61	3,96E-12	1131	72859	-2,92	1,42E-17
632	123648	-1,79	2,09E-14	732	68338	-1,94	1,28E-86	832	106781	-2,13	2,15E-80	932	41501	-2,35	1,61E-11	1032	112567	-2,61	2,71E-43	1132	3434	-2,92	2,39E-44
633	120943	-1,79	9,33E-32	733	67024	-1,94	3,02E-05	833	58772	-2,13	2,11E-43	933	56896	-2,36	4,63E-31	1033	69228	-2,61	5,96E-69	1133	123475	-2,92	1,15E-166
634	62100	-1,79	1,36E-03	734	111119	-1,94	5,54E-03	834	121336	-2,13	1,23E-26	934	74580	-2,36	2,24E-51	1034	60329	-2,61	1,72E-02	1134	68755	-2,93	2,38E-19
635	55179	-1,80	9,52E-19	735	106452	-1,95	1,20E-28	835	111875	-2,13	2,21E-54	935	121439	-2,36	9,72E-05	1035	119779	-2,61	3,37E-74	1135	123456	-2,93	3,49E-147
636	111179	-1,80	1,82E-04	736	65915	-1,95	2,59E-21	836	122448	-2,13	4,43E-22	936	44214	-2,36	1,91E-82	1036	120911	-2,61	3,24E-71	1136	81586	-2,93	8,34E-157
637	102497	-1,80	9,81E-54	737	106646	-1,95	1,65E-38	837	70910	-2,14	8,31E-12	937	3787	-2,36	3,84E-31	1037	81865	-2,62	4,44E-87	1137	53859	-2,93	4,68E-20
638	43101	-1,80	1,14E-05	738	112643	-1,95	2,49E-10	838	70830	-2,14	1,42E-21	938	105260	-2,36	8,03E-04	1038	72321	-2,62	1,10E-33	1138	105171	-2,94	5,84E-03
639	102985	-1,81	7,61E-48	739	120851	-1,95	2,23E-11	839	122160	-2,14	5,53E-19	939	52438	-2,37	1,04E-66	1039	5656	-2,62	1,08E-34	1139	107297	-2,94	3,57E-18
640	76734	-1,81	8,86E-13	740	64370	-1,96	4,39E-16	840	46819	-2,14	3,83E-04	940	111966	-2,37	1,11E-03	1040	80578	-2,63	4,70E-13	1140	67579	-2,95	2,90E-13
641	120030	-1,81	7,94E-59	741	123888	-1,96	7,15E-27	841	123368	-2,15	1,35E-71	941	53501	-2,37	2,06E-45	1041	122724	-2,63	6,62E-31	1141	82626	-2,95	2,86E-17
642	61374	-1,81	7,82E-03	742	80871	-1,96	1,26E-35	842	49946	-2,15	2,81E-22	942	104173	-2,37	8,19E-05	1042	103063	-2,63	4,69E-33	1142	121986	-2,95	6,70E-40
643	81148	-1,81	1,60E-52	743	108833	-1,96	2,20E-25	843	112665	-2,15	2,63E-07	943	4726	-2,37	1,14E-88	1043	54230	-2,63	7,31E-04	1143	65067	-2,96	3,37E-17
644	80645	-1,81	4,37E-06	744	70316	-1,96	4,97E-10	844	122168	-2,16	1,67E-38	944	42267	-2,37	2,44E-04	1044	62285	-2,63	2,66E-17	1144	106615	-2,96	1,15E-02
645	76677	-1,81	1,61E-41	745	72612	-1,96	2,05E-58	845	103048	-2,17	1,56E-37	945	23332	-2,37	3,74E-54	1045	106821	-2,63	3,62E-03	1145	70639	-2,96	1,26E-06
646	102851	-1,82	2,01E-08	746	122076	-1,96	4,21E-56	846	58475	-2,18	2,54E-13	946	63202	-2,38	4,22E-22	1046	112081	-2,64	2,05E-102	1146	79816	-2,97	4,58E-49
647	1941	-1,82	4,81E-02	747	61517	-1,96	2,94E-65	847	66517	-2,18	3,84E-40	947	112001	-2,38	9,59E-30	1047	26642	-2,64	3,76E-76	1147	123650	-2,97	8,82E-109
648	111137	-1,82	5,40E-05	748	70351	-1,96	5,60E-17	848	21873	-2,18	1,41E-28	948	66935	-2,39	1,17E-36	1048	122124	-2,65	8,18E-93	1148	122376	-2,98	2,38E-106
649	75989	-1,82	4,29E-61	749	104073	-1,97	8,11E-05	849	123659	-2,18	3,33E-09	949	75704	-2,39	1,24E-67	1049	49979	-2,65	1,76E-153	1149	123733	-3,00	7,73E-138
650	63868	-1,82	1,53E-14	750	68606	-1,97	6,70E-42	850	59900	-2,18	7,03E-06	950	110440	-2,39	3,95E-07	1050	112105	-2,65	1,83E-55	1150	107641	-3,00	4,98E-02
651	44389	-1,82	1,66E-48	751	103825	-1,97	3,29E-02	851	62447	-2,18	1,03E-73	951	65743	-2,39	3,83E-12	1051	44640	-2,66	1,81E-100	1151	70956	-3,00	2,97E-03
652	78462	-1,83	3,22E-09	752	122527	-1,97	5,78E-26	852	120696	-2,19	3,65E-09	952	104219	-2,41	5,72E-33	1052	105844	-2,66	3,93E-07	1152	104222	-3,00	2,86E-44
653	103920	-1,83	7,25E-28	753	120311	-1,97	3,63E-44	853	55375	-2,19	2,67E-79	953	56291	-2,41	1,45E-18	1053	104097	-2,66	4,65E-13	1153	73623	-3,01	1,51E-04
654	119826	-1,83	1,31E-48	754	60085	-1,97	3,98E-02	854	66016	-2,19	2,64E-40	954	58774	-2,41	4,03E-05	1054	47432	-2,66	4,33E-98	1154	5836	-3,02	4,14E-37
655	54954	-1,83	1,32E-15	755	124277	-1,97	2,67E-03	855	123149	-2,19	1,05E-48	955	123262	-2,42	6,26E-35	1055	110790	-2,66	1,01E-28	1155	2015	-3,02	3,11E-76
656	53428	-1,83	8,95E-15	756	60518	-1,97	1,11E-13	856	110382	-2,19	1,16E-02	956	75105	-2,42	1,26E-32	1056	55240	-2,67	8,73E-08	1156	54444	-3,02	4,87E-16
657	104377	-1,83	9,01E-07	757	103060	-1,97	2,11E-02	857	110651	-2,19	1,41E-02	957	81964	-2,42	1,94E-06	1057	49649	-2,67	3,44E-94	1157	103147	-3,03	8,67E-160
658	65992	-1,84	2,94E-37	758	121232	-1,97	2,38E-45	858	122065	-2,20	1,07E-96	958	66092	-2,42	1,15E-18	1058	120371	-2,67	3,59E-02	1158	41590	-3,03	1,07E-63
659	81058	-1,84	2,00E-04	759	59746	-1,98	2,26E-04	859	107856	-2,20	1,23E-03	959	59642	-2,43	5,93E-29	1059	3055	-2,67	3,10E-10	1159	34274	-3,03	9,14E-103
660	110455	-1,84	2,48E-02	760	67866	-1,98	4,30E-02	860	64271	-2,20	2,28E-53	960	66598	-2,43	5,85E-14	1060	110262	-2,68	5,66E-17	1160	104081	-3,04	9,89E-14
661	104320	-1,84	2,08E-14	761	123797	-1,98	1,03E-46	861	69651	-2,20	1,41E-04	961	105313	-2,44	3,25E-43	1061	105823	-2,69	7,09E-04	1161	104419	-3,04	1,17E-42
662	107776	-1,84	1,95E-26	762	121126	-1,98	5,98E-49	862	71823	-2,20	3,34E-17	962	121735	-2,44	2,15E-70	1062	70491	-2,69	4,35E-04	1162	35109	-3,05	1,29E-06
663	65718	-1,84	1,34E-29	763	65190	-1,98	1,98E-02	863	72488	-2,21	1,03E-22	963	52476	-2,44	1,49E-43	1063	59197	-2,69	8,40E-17	1163	106623	-3,05	2,46E-11
664	61383	-1,84	1,01E-36	764	120424	-1,98	1,28E-58	864	62971	-2,21	4,17E-09	964	121746	-2,44	1,03E-106	1064	21509	-2,69	2,33E-22	1164	63125	-3,06	6,45E-18
665	56700	-1,84	6,96E-04	765	4104	-1,98	1,26E-19	865	60981	-2,21	9,48E-63	965	107947	-2,45	2,34E-57	1065	64710	-2,70	2,46E-84	1165	106223	-3,06	2,4E-166
666	121130	-1,85	4,32E-32	766	102561	-1,98	1,01E-03	866	51686	-2,21	6,41E-69	966	82235	-2,45	6,62E-92	1066	5651	-2,70	5,00E-13	1166	79111	-3,07	2,8E-127
667	120983	-1,85	3,66E-62	767	110765	-1,98	6,39E-03	867	122095	-2,21	1,45E-31	967	46443	-2,45	6,30E-47	1067	66117	-2,70	2,13E-32	1167	110316	-3,07	6,1E-115
668	106294	-1,85	9,52E-11	768	107871	-1,98	1,19E-50	868	62166	-2,22	5,98E-40	968	2211	-2,45	2,15E-04	1068	123806	-2,71	1,54E-14	1168	103006	-3,08	1,45E-08
669	105220	-1,85	1,97E-02	769	78970	-1,99	9,07E-61	869	54667	-2,22	2,36E-36	969	6103	-2,45	1,40E-02	1069	58698	-2,71	6,00E-33	1169	124056	-3,08	0,000156
670	65869	-1,85	3,26E-13	770	112288	-1,99	9,12E-04	870	50996	-2,22	2,52E-37	970	34277	-2,45	1,20E-35	1070	3481	-2,72	3,14E-52	1170	5359	-3,08	1,68E-53
671	123476	-1,85	1,75E-59	771	1885	-1,99	9,70E-61	871	71080	-2,23	3,34E-10	971	62651	-2,46	2,46E-56	1071	59508	-2,72	9,40E-03	1171	66551	-3,08	2,42E-05
672	122422	-1,86	2,44E-28	772	107475	-1,99	6,34E-31	872	2837	-2,23	1,42E-03	972	76215	-2,46	3,02E-141	1072	106480	-2,72	8,48E-11	1172	58396	-3,08	4,99E-09
673	65957	-1,86	1,74E-06	773	46816	-1,99	1,75E-09	873	41152	-2,23	5,98E-09	973	102487	-2,46	4,39E-13	1073	23083	-2,72	1,69E-57	1173	112596	-3,09	1,8E-102
674	74122	-1,86	2,99E-32	774	110653	-1,99	3,77E-74	874	56418	-2,23	5,01E-03	974	41149	-2,46	4,99E-34	1074	32364	-2,72	7,74E-12	1174	40830	-3,09	4,81E-56
675	65209	-1,86	2,92E-21	775	57045	-1,99	4,32E-06	875	59082	-2,23	6,45E-04	975	121074	-2,46	7,26E-69	1075	107229	-2,73	3,86E-02	1175	70894	-3,09	0,000306
676	59362	-1,86	1,57E-05	776	120489	-1,99	3,97E-54	876	110311	-2,24	1,84E-03	976	106314	-2,47	8,18E-46	1076	111682	-2,73	1,12E-09	1176	78626	-3,10	1,2E-09
677	121214	-1,86	4,75E-50	777	107202	-2,00	1,34E-07	877	1999	-2,25	2,29E-18	977	74156	-2,47	6,62E-32	1077	69537	-2,73	1,29E-03	1177	109101	-3,11	7,63E-11
678	46446	-1,86	9,39E-52	778	109338	-2,00	1,49E-09	878	111362	-2,25	1,48E-17	978	123779	-2,48	1,64E-07	1078	43701	-2,73	2,62E-184	1178	103039	-3,11	2,86E-17
679	112126	-1,87	8,95E-19	779	71532	-2,00	6,20E-07	879	123987	-2,25	1,12E-33	979	81430	-2,48	7,63E-09	1079	64971	-2,74	1,31E-19	1179	70842	-3,11	1,54E-05
680	52521	-1,87	1,36E-14	780	62091	-2,01	6,08E-07	880	112125	-2,25	3,29E-18	980	74987	-2,48	1,94E-92	1080	67971	-2,74	1,86E-34	1180	65315	-3,12	5,12E-42
681	51407	-1,87	3,08E-52	781	124043	-2,01	1,60E-02	881	22785	-2,25	8,35E-67	981	109850	-2,48	1,72E-70	1081	112014	-2,74	1,71E-02	1181	111429	-3,13	0,002906
682	5270	-1,87	4,13E-06	782	122208	-2,01	5,59E-40	882	103747	-2,26	3,22E-11	982	121475	-2,48	1,46E-83	1082	59338	-2,74	6,06E-07	1182	121990	-3,13	6,31E-86
683	108463	-1,87	4,43E-40	783	52875	-2,01	1,63E-54	883	103180	-2,26	6,76E-39	983	119956	-2,48	4,65E-93	1083	107674	-2,75	1,52E-12	1183	103671	-3,13	1,22E-79
684	68590	-1,87	1,82E-04	784	68279	-2,01	9,33E-18	884	77795	-2,26	8,23E-125	984	22386	-2,48	2,80E-58	1084	105238	-2,75	4,47E-13	1184	69026	-3,13	3,04E-37
685	76696	-1,87	1,36E-29	785	109201	-2,01	1,27E-17	885	78357	-2,26	5,71E-30	985	69652	-2,49	5,01E-24	1085	77785	-2,75	3,78E-51	1185	108025	-3,14	0,028993
686	76713	-1,88	7,51E-46	786	109641	-2,01	3,85E-02	886	121139	-2,26	3,86E-70	986	47153	-2,49	3,68E-48	1086	108686	-2,75	5,48E-25	1186	111451	-3,14	2,09E-16
687	106706	-1,88	2,07E-22	787	107802	-2,01	7,30E-09	887	59354	-2,27	8,68E-35	987	121399	-2,49	5,60E-78	1087	111742	-2,76	1,96E-31	1187	62213	-3,14	4,9E-169
688	22739	-1,88	7,62E-62	788	72922	-2,02	5,78E-29	888	44117	-2,27	7,60E-69	988	66689	-2,49	2,64E-04	1088	67616	-2,76	1,40E-109	1188	111495	-3,14	0,006791
689	57280	-1,88	3,30E-05	789	62172	-2,02	4,03E-04	889	35552	-2,27	8,91E-33	989	110850	-2,49	3,36E-10	1089	121415	-2,76	5,56E-10	1189	62153	-3,14	1,4E-89
690	124338	-1,88	1,63E-04	790	63756	-2,02	6,55E-05	890	70630	-2,27	2,16E-52	990	123681	-2,49	1,87E-85	1090	124282	-2,76	7,34E-130	1190	61526	-3,15	1,25E-40
691	2492	-1,88	5,11E-22	791	69115	-2,02	6,20E-28	891	120597	-2,27	1,09E-42	991	105239	-2,49	6,38E-43	1091	53673	-2,76	8,58E-95	1191	109373	-3,16	2,56E-32
692	111724	-1,89	3,42E-06	792	77747	-2,02	3,29E-49	892	105216	-2,28	4,87E-11	992	32716	-2,50	1,85E-03	1092	69605	-2,77	7,95E-03	1192	61078	-3,16	2,01E-71
693	120357	-1,89	7,41E-78	793	71094	-2,02	2,59E-02	893	67538	-2,28	1,47E-05	993	3716	-2,50	1,40E-55	1093	61293	-2,78	3,16E-06	1193	3823	-3,16	5,3E-129
694	120110	-1,89	1,01E-58	794	109317	-2,02	8,60E-26	894	103112	-2,28	7,99E-17	994	123787	-2,50	1,28E-14	1094	121111	-2,78	7,24E-17	1194	82623	-3,17	1,75E-80
695	74745	-1,89	5,71E-19	795	71556	-2,02	2,61E-02	895	74103	-2,28	9,78E-56	995	56350	-2,50	6,79E-05	1095	61121	-2,78	2,24E-43	1195	81096	-3,17	4,1E-147
696	58117	-1,89	1,54E-68	796	110893	-2,03	2,46E-12	896	121661	-2,28	3,08E-35	996	119839	-2,50	9,32E-27	1096	109146	-2,78	4,30E-04	1196	111887	-3,17	0,000286
697	63395	-1,89	3,29E-07	797	72627	-2,03	1,11E-04	897	105838	-2,29	1,06E-11	997	60835	-2,50	1,67E-97	1097	56370	-2,79	3,90E-23	1197	28036	-3,18	1,24E-09
698	21836	-1,89	2,03E-50	798	57112	-2,03	3,16E-15	898	4876	-2,29	4,24E-15	998	82049	-2,51	3,40E-79	1098	82374	-2,80	1,59E-121	1198	123777	-3,18	1,08E-98
699	108852	-1,90	7,33E-03	799	64937	-2,03	2,43E-02	899	74282	-2,29	2,59E-06	999	107076	-2,51	4,55E-02	1099	67109	-2,80	5,57E-18	1199	54242	-3,18	1,03E-81
700	79271	-1,90	1,21E-46	800	61504	-2,04	1,04E-03	900	59073	-2,30	2,76E-21	1000	110853	-2,51	7,14E-65	1100	78683	-2,81	6,50E-116	1200	105155	-3,18	0,02216

Supplementary Table S10 (continued): FULL list of DEGs from RNA-Seq data for wild-type grown on GlcNAc and compared to control (WT glycerol).

Differentially expressed genes (DEGs) were defined by fold change ≥|1.0| and a adjusted p-value (padj) of < 0.05 found by DESeq2 (RStudio 2022.07.2+576)

	Identifier (TRIREDRAFT)	log2fc	padj		Identifier (TRIREDRAFT)	log2fc	padj		Identifier (TRIREDRAFT)	log2fc	padj		Identifier (TRIREDRAFT)	log2fc	padj
1201	4744	-3,20	2,01E-73	1301	66534	-3,58	3,26E-02	1401	108585	-4,33	3,95E-03	1501	80086	-5,71	9,70E-76
1202	81979	-3,20	5,02E-91	1302	82041	-3,59	8,66E-16	1402	105977	-4,33	1,44E-20	1502	111251	-5,76	1,22E-86
1203	66276	-3,20	1,04E-15	1303	58584	-3,59	1,03E-208	1403	124198	-4,34	2,14E-08	1503	111838	-5,77	5,30E-112
1204	67717	-3,22	1,10E-89	1304	58563	-3,59	6,57E-03	1404	65410	-4,34	0,00E+00	1504	60419	-5,77	1,56E-05
1205	74187	-3,23	2,49E-14	1305	47424	-3,59	1,68E-52	1405	105445	-4,35	8,96E-17	1505	122079	-5,77	1,22E-275
1206	112521	-3,24	9,26E-04	1306	58910	-3,59	4,58E-18	1406	81659	-4,35	2,84E-64	1506	69901	-5,78	6,29E-05
1207	121107	-3,24	1,11E-211	1307	5787	-3,60	1,19E-16	1407	69245	-4,35	2,20E-11	1507	68812	-5,78	0,00E+00
1208	109117	-3,24	1,74E-32	1308	120747	-3,60	1,03E-33	1408	122324	-4,37	3,69E-03	1508	111082	-5,79	0,00E+00
1209	56314	-3,25	2,08E-02	1309	109278	-3,60	3,61E-02	1409	106626	-4,37	3,27E-87	1509	3488	-5,80	2,89E-42
1210	105251	-3,25	2,68E-44	1310	121800	-3,61	5,98E-127	1410	105820	-4,37	1,62E-05	1510	110127	-5,80	0,00E+00
1211	5016	-3,25	3,76E-14	1311	123946	-3,62	0,00E+00	1411	120359	-4,38	0,00E+00	1511	73818	-5,96	0,00E+00
1212	106161	-3,26	1,22E-33	1312	103012	-3,62	4,04E-40	1412	112019	-4,38	5,73E-14	1512	69650	-5,99	1,64E-213
1213	77557	-3,26	2,82E-112	1313	105154	-3,62	3,36E-02	1413	56835	-4,41	2,67E-08	1513	3094	-6,03	1,10E-54
1214	107524	-3,26	9,20E-104	1314	105287	-3,63	1,00E-03	1414	65097	-4,42	2,91E-17	1514	64869	-6,04	4,37E-06
1215	110778	-3,26	1,34E-28	1315	70197	-3,65	8,73E-10	1415	50793	-4,42	1,89E-32	1515	123086	-6,09	0,00E+00
1216	81410	-3,27	6,97E-121	1316	111832	-3,66	1,12E-155	1416	54461	-4,42	1,11E-74	1516	121136	-6,10	0,00E+00
1217	122505	-3,27	9,04E-100	1317	58639	-3,66	8,53E-04	1417	56860	-4,44	2,03E-97	1517	3267	-6,15	8,68E-38
1218	5127	-3,27	7,05E-31	1318	111053	-3,67	6,87E-69	1418	109846	-4,45	3,37E-07	1518	22331	-6,20	2,82E-220
1219	63687	-3,28	3,50E-45	1319	107844	-3,69	6,58E-03	1419	58814	-4,46	2,84E-04	1519	59876	-6,24	2,45E-41
1220	80639	-3,28	1,15E-118	1320	109372	-3,69	4,03E-22	1420	72704	-4,47	2,47E-47	1520	110457	-6,26	1,11E-103
1221	23382	-3,28	7,42E-04	1321	108749	-3,69	2,40E-195	1421	104260	-4,47	2,74E-03	1521	69692	-6,28	3,99E-12
1222	102492	-3,29	2,31E-02	1322	57088	-3,70	3,34E-103	1422	78401	-4,47	0,00E+00	1522	65039	-6,30	1,59E-107
1223	106315	-3,29	1,43E-40	1323	59078	-3,70	1,38E-14	1423	105449	-4,48	9,81E-03	1523	69696	-6,37	9,78E-07
1224	121653	-3,29	2,24E-11	1324	22654	-3,71	7,57E-151	1424	80922	-4,49	0,00E+00	1524	106660	-6,46	5,96E-07
1225	65854	-3,30	1,45E-18	1325	70520	-3,71	3,43E-143	1425	109944	-4,49	8,08E-06	1525	73101	-6,55	3,93E-07
1226	60676	-3,30	1,02E-167	1326	68588	-3,72	1,85E-149	1426	65333	-4,50	9,18E-234	1526	105269	-6,62	3,02E-07
1227	69863	-3,32	5,45E-104	1327	111874	-3,74	1,18E-17	1427	82633	-4,50	4,24E-281	1527	110740	-6,79	1,09E-07
1228	112439	-3,32	1,68E-74	1328	102500	-3,74	8,39E-14	1428	112034	-4,51	2,95E-15	1528	70365	-6,81	2,51E-63
1229	123976	-3,32	2,26E-53	1329	47066	-3,74	1,17E-243	1429	108155	-4,53	2,10E-04	1529	69834	-6,83	0,00E+00
1230	124084	-3,33	7,28E-39	1330	121133	-3,76	5,88E-274	1430	108577	-4,54	7,29E-03	1530	70934	-6,98	2,21E-07
1231	104304	-3,33	1,52E-02	1331	111672	-3,79	4,31E-03	1431	106591	-4,54	7,31E-03	1531	120923	-7,03	1,91E-60
1232	78072	-3,33	1,81E-04	1332	49274	-3,79	1,38E-103	1432	74194	-4,55	3,55E-11	1532	77552	-7,31	8,22E-99
1233	107857	-3,34	3,69E-75	1333	62502	-3,82	4,52E-182	1433	103205	-4,55	3,05E-10	1533	107881	-7,58	0,00E+00
1234	53903	-3,34	2,84E-47	1334	111403	-3,82	4,75E-03	1434	64167	-4,55	3,62E-145	1534	111122	-7,93	7,28E-100
1235	61223	-3,35	2,96E-82	1335	4774	-3,83	2,65E-09	1435	58333	-4,56	2,20E-76	1535	103061	-8,19	5,78E-11
1236	110452	-3,35	3,87E-128	1336	104277	-3,83	1,64E-14	1436	107949	-4,57	9,66E-03	1536	106928	-8,71	1,52E-117
1237	106686	-3,35	5,51E-72	1337	122350	-3,83	1,11E-270	1437	56840	-4,57	1,76E-18	1537	103062	-8,72	1,58E-45
1238	55319	-3,36	1,77E-30	1338	4517	-3,85	1,93E-08	1438	122780	-4,59	6,86E-162	1538	111121	-8,85	2,12E-95
1239	76360	-3,36	1,02E-04	1339	81022	-3,85	8,65E-124	1439	67509	-4,60	4,40E-11	1539	60489	-9,14	9,83E-14
1240	70991	-3,36	2,00E-12	1340	2316	-3,86	6,56E-176	1440	76620	-4,60	0,00E+00	1540	30465	-9,50	6,22E-37
1241	61350	-3,36	2,32E-189	1341	5119	-3,87	1,13E-189	1441	41660	-4,62	1,94E-04	1541	105882	-9,70	3,11E-15
1242	53331	-3,37	1,55E-38	1342	23292	-3,87	2,57E-50	1442	121498	-4,67	2,19E-222	1542			
1243	110712	-3,37	2,08E-02	1343	107853	-3,87	2,14E-10	1443	79741	-4,70	0,00E+00	1543			
1244	59333	-3,39	2,35E-03	1344	52222	-3,88	1,91E-66	1444	73102	-4,70	1,27E-04	1544			
1245	62716	-3,39	1,75E-203	1345	69181	-3,88	4,35E-02	1445	102499	-4,70	7,31E-17	1545			
1246	107667	-3,39	3,82E-04	1346	69904	-3,88	5,58E-67	1446	111239	-4,71	1,41E-06	1546			
1247	22915	-3,40	5,04E-15	1347	22110	-3,89	1,43E-34	1447	77806	-4,71	0,00E+00	1547			
1248	58853	-3,40	5,34E-151	1348	47930	-3,91	4,87E-235	1448	68585	-4,72	1,76E-08	1548			
1249	109828	-3,41	7,49E-66	1349	66827	-3,91	7,72E-21	1449	76800	-4,75	0,00E+00	1549			
1250	123468	-3,41	1,96E-13	1350	106695	-3,91	3,33E-65	1450	58472	-4,76	1,05E-06	1550			
1251	68662	-3,41	1,03E-29	1351	66888	-3,92	3,88E-48	1451	76601	-4,77	8,99E-16	1551			
1252	111306	-3,41	4,72E-02	1352	104272	-3,93	8,35E-18	1452	70375	-4,79	8,55E-75	1552			
1253	30776	-3,41	1,33E-02	1353	122795	-3,93	1,50E-98	1453	72581	-4,79	2,97E-12	1553			
1254	109307	-3,42	2,75E-11	1354	106627	-3,94	2,04E-80	1454	55374	-4,81	1,97E-43	1554			
1255	106584	-3,42	4,57E-02	1355	4860	-3,94	9,79E-86	1455	66766	-4,82	2,17E-08	1555			
1256	59391	-3,43	1,41E-16	1356	105291	-3,95	7,63E-63	1456	108143	-4,83	2,67E-31	1556			
1257	56911	-3,43	5,12E-15	1357	122108	-3,96	4,48E-163	1457	109239	-4,85	3,32E-49	1557			
1258	121405	-3,44	5,35E-90	1358	105840	-3,96	1,42E-219	1458	52315	-4,86	3,72E-222	1558			
1259	69751	-3,45	2,56E-36	1359	121717	-3,97	7,62E-11	1459	73250	-4,87	1,30E-37	1559			
1260	123827	-3,45	8,38E-104	1360	70962	-3,97	1,21E-02	1460	23353	-4,87	0,00E+00	1560			
1261	2852	-3,46	1,77E-24	1361	81082	-3,98	6,87E-18	1461	59655	-4,92	4,33E-05	1561			
1262	79738	-3,47	2,62E-97	1362	122825	-3,98	3,35E-79	1462	61553	-4,95	2,34E-23	1562			
1263	122820	-3,48	1,24E-02	1363	105805	-3,99	3,68E-02	1463	103033	-4,97	4,56E-04	1563			
1264	76238	-3,48	1,78E-24	1364	123260	-3,99	3,43E-02	1464	122089	-4,98	1,33E-15	1564			
1265	46128	-3,48	2,11E-22	1365	65986	-3,99	2,73E-09	1465	108781	-4,98	3,63E-126	1565			
1266	69956	-3,48	1,72E-58	1366	122301	-4,01	1,62E-248	1466	123718	-4,99	1,52E-227	1566			
1267	123697	-3,49	1,12E-17	1367	122792	-4,01	4,77E-74	1467	103049	-4,99	8,02E-24	1567			
1268	108684	-3,49	4,43E-35	1368	106661	-4,01	3,49E-02	1468	111837	-5,01	5,07E-220	1568			
1269	76359	-3,49	2,42E-94	1369	112532	-4,02	7,55E-06	1469	103149	-5,02	3,21E-189	1569			
1270	71059	-3,50	1,29E-06	1370	103189	-4,03	7,97E-14	1470	62872	-5,05	3,44E-13	1570			
1271	123079	-3,50	3,42E-20	1371	106606	-4,04	6,16E-22	1471	69529	-5,10	1,05E-03	1571			
1272	28409	-3,50	2,31E-04	1372	80607	-4,06	4,32E-12	1472	66345	-5,10	0,00E+00	1572			
1273	56771	-3,50	4,78E-31	1373	105533	-4,07	7,32E-102	1473	123979	-5,12	0,00E+00	1573			
1274	3292	-3,50	1,49E-03	1374	108583	-4,09	1,10E-34	1474	109249	-5,14	2,66E-116	1574			
1275	31658	-3,51	3,70E-02	1375	64922	-4,12	9,51E-21	1475	70527	-5,17	7,71E-06	1575			
1276	58675	-3,51	6,47E-45	1376	110456	-4,12	5,85E-47	1476	108784	-5,17	1,23E-21	1576			
1277	45343	-3,51	4,05E-26	1377	59351	-4,14	1,16E-08	1477	74214	-5,18	1,11E-50	1577			
1278	70098	-3,51	1,44E-15	1378	111881	-4,18	7,03E-03	1478	109370	-5,18	1,44E-03	1578			
1279	76971	-3,52	1,08E-102	1379	58356	-4,18	0,00E+00	1479	5789	-5,22	4,35E-15	1579			
1280	70961	-3,52	2,53E-90	1380	23240	-4,18	7,75E-60	1480	119864	-5,23	3,53E-29	1580			
1281	52489	-3,52	1,04E-264	1381	107843	-4,18	2,70E-02	1481	105156	-5,26	1,86E-08	1581			
1282	50583	-3,53	1,82E-40	1382	33723	-4,19	2,29E-02	1482	65191	-5,27	8,43E-86	1582			
1283	110761	-3,53	1,18E-165	1383	60067	-4,20	3,13E-118	1483	121127	-5,29	4,44E-04	1583			
1284	120198	-3,54	4,14E-145	1384	103065	-4,20	4,84E-05	1484	105810	-5,33	1,04E-04	1584			
1285	123539	-3,54	1,93E-17	1385	71394	-4,21	7,52E-03	1485	121739	-5,34	1,56E-47	1585			
1286	72632	-3,54	8,42E-03	1386	109331	-4,21	2,01E-02	1486	121298	-5,39	2,95E-261	1586			
1287	111912	-3,54	1,02E-56	1387	108018	-4,23	1,82E-47	1487	69555	-5,45	4,15E-04	1587			
1288	70334	-3,55	4,61E-33	1388	76366	-4,23	1,36E-24	1488	59065	-5,48	3,64E-09	1588			
1289	75380	-3,55	9,61E-03	1389	64049	-4,23	1,09E-35	1489	122506	-5,50	2,87E-138	1589			
1290	66616	-3,55	2,38E-85	1390	59402	-4,23	6,67E-24	1490	123673	-5,51	2,11E-63	1590			
1291	2036	-3,55	9,76E-31	1391	112633	-4,24	6,02E-03	1491	5182	-5,55	1,93E-14	1591			
1292	108676	-3,55	5,53E-17	1392	77547	-4,24	4,29E-97	1492	103179	-5,55	3,27E-103	1592			
1293	66865	-3,56	2,09E-75	1393	81576	-4,26	0,00E+00	1493	58366	-5,56	1,95E-04	1593			
1294	80920	-3,56	5,21E-13	1394	105282	-4,26	5,63E-257	1494	110711	-5,57	2,14E-09	1594			
1295	111739	-3,56	3,34E-119	1395	68831	-4,27	5,08E-33	1495	121350	-5,60	0,00E+00	1595			
1296	43129	-3,56	3,03E-70	1396	122824	-4,28	7,39E-46	1496	5330	-5,62	3,27E-05	1596			
1297	4171	-3,57	9,77E-03	1397	105860	-4,30	5,04E-301	1497	61605	-5,65	9,57E-05	1597			
1298	32798	-3,57	8,39E-190	1398	109945	-4,31	4,13E-104	1498	112491	-5,68	2,63E-05	1598			
1299	121839	-3,57	4,87E-174	1399	111888	-4,32	1,44E-19	1499	43312	-5,68	4,48E-113	1599			
1300	76247	-3,57	3,32E-135	1400	68466	-4,32	2,49E-45	1500	111750	-5,69	0,00E+00	1600			

Supplementary Table S11: FULL list of DEGs from RNA-Seq data for Δ*ngs1* (jgi|Trire2|79669) grown on GlcNAc and compared to control (WT grown on GlcNAc).

Differentially expressed genes (DEGs) were defined by fold change ≥|1.0| and a adjusted p-value (padj) of < 0.05 found by DESeq2 (RStudio 2022.07.2+576)

	Identifier (TRIREDRAFT)	log2fc	padj		Identifier (TRIREDRAFT)	log2fc	padj		Identifier (TRIREDRAFT)	log2fc	padj		Identifier (TRIREDRAFT)	log2fc	padj		Identifier (TRIREDRAFT)	log2fc	padj		Identifier (TRIREDRAFT)	log2fc	padj
1	79669	-10,50	3,27E-88	101	67806	-4,04	5,62E-92	201	124295	-2,95	3,89E-39	301	109371	-2,40	3,61E-17	401	120064	-2,03	1,51E-57	501	22914	-1,83	6,12E-88
2	79816	-8,89	2,45E-51	102	103049	-4,04	3,94E-35	202	111865	-2,95	1,21E-04	302	46128	-2,40	8,15E-19	402	106680	-2,03	5,84E-38	502	3641	-1,82	1,88E-37
3	55374	-8,46	2,01E-22	103	22110	-4,03	5,33E-51	203	76374	-2,94	3,70E-34	303	59272	-2,40	2,34E-36	403	67008	-2,02	6,24E-06	503	63657	-1,81	1,74E-34
4	111121	-8,42	6,19E-155	104	123979	-4,01	0,00E+00	204	103179	-2,92	4,06E-36	304	105171	-2,39	1,22E-02	404	122572	-2,02	5,05E-62	504	121785	-1,81	6,71E-06
5	106660	-8,09	2,46E-10	105	65817	-4,00	5,87E-03	205	69529	-2,92	1,06E-02	305	111881	-2,39	2,04E-02	405	54064	-2,01	1,20E-44	505	63107	-1,81	3,57E-06
6	77552	-7,46	2,22E-134	106	65039	-4,00	5,29E-66	206	54242	-2,92	1,88E-72	306	81442	-2,38	5,61E-57	406	105848	-2,01	6,62E-16	506	123946	-1,81	1,88E-72
7	106928	-7,30	6,51E-224	107	63125	-4,00	4,57E-26	207	65615	-2,91	1,21E-27	307	53673	-2,37	4,76E-71	407	111345	-2,01	6,49E-07	507	108299	-1,81	1,49E-25
8	73623	-7,29	5,81E-08	108	64167	-3,94	1,51E-168	208	108684	-2,91	2,15E-38	308	120923	-2,36	8,00E-05	408	23382	-2,01	2,08E-03	508	80792	-1,81	2,05E-43
9	5330	-7,24	4,98E-08	109	55566	-3,94	3,09E-08	209	56384	-2,90	1,14E-76	309	82049	-2,36	1,90E-62	409	59472	-2,01	8,75E-11	509	74442	-1,80	3,36E-34
10	105882	-6,93	9,10E-28	110	111837	-3,92	3,51E-101	210	23292	-2,88	2,27E-34	310	105287	-2,36	1,44E-03	410	22264	-2,01	8,04E-64	510	121074	-1,80	1,26E-49
11	110740	-6,56	3,41E-09	111	61526	-3,91	7,10E-62	211	81819	-2,88	3,91E-03	311	53053	-2,36	2,22E-25	411	123733	-2,00	3,06E-59	511	60337	-1,80	7,10E-03
12	107881	-6,52	0,00E+00	112	109331	-3,91	2,25E-02	212	81096	-2,88	1,10E-51	312	109101	-2,36	8,38E-10	412	123648	-2,00	1,72E-19	512	107835	-1,80	9,77E-05
13	121350	-6,33	0,00E+00	113	68585	-3,89	5,10E-12	213	79623	-2,87	4,14E-57	313	70996	-2,35	1,42E-25	413	76722	-2,00	2,09E-54	513	78836	-1,80	8,33E-54
14	111122	-6,30	0,00E+00	114	60182	-3,88	1,02E-11	214	68466	-2,86	3,35E-39	314	82296	-2,35	5,15E-80	414	105242	-2,00	1,75E-49	514	105977	-1,80	1,30E-11
15	109239	-6,29	8,46E-55	115	69956	-3,85	9,87E-54	215	105313	-2,85	3,61E-47	315	22251	-2,35	1,38E-42	415	108683	-2,00	5,10E-05	515	122296	-1,80	3,98E-43
16	58366	-6,22	2,14E-05	116	69692	-3,85	3,84E-21	216	3434	-2,85	1,63E-34	316	65883	-2,34	3,09E-07	416	75165	-1,99	4,17E-22	516	59770	-1,79	1,48E-04
17	102499	-6,17	3,18E-18	117	61223	-3,85	2,78E-127	217	74156	-2,84	2,16E-38	317	70949	-2,32	2,94E-12	417	3671	-1,99	1,04E-41	517	106315	-1,79	2,73E-24
18	103061	-6,16	1,07E-22	118	82032	-3,84	3,58E-130	218	112665	-2,84	4,60E-12	318	124282	-2,32	6,89E-94	418	111739	-1,99	2,63E-40	518	109443	-1,79	7,19E-67
19	30465	-6,12	2,95E-143	119	103193	-3,82	3,03E-02	219	67013	-2,83	8,76E-08	319	123475	-2,32	1,21E-100	419	107037	-1,99	3,15E-12	519	65179	-1,78	2,00E-76
20	111082	-6,10	0,00E+00	120	104067	-3,80	9,99E-06	220	122523	-2,83	1,88E-70	320	124333	-2,32	3,28E-24	420	112532	-1,99	1,35E-04	520	105968	-1,78	1,68E-09
21	69650	-6,00	2,49E-251	121	112034	-3,78	1,38E-20	221	43671	-2,83	1,36E-175	321	56911	-2,32	1,46E-12	421	81599	-1,99	1,74E-79	521	109793	-1,77	3,99E-04
22	123086	-5,94	2,60E-246	122	112643	-3,77	1,89E-25	222	66517	-2,83	2,07E-69	322	122792	-2,31	7,88E-53	422	110173	-1,99	1,94E-57	522	122531	-1,77	8,61E-43
23	108018	-5,94	6,98E-61	123	76800	-3,75	0,00E+00	223	68662	-2,82	3,41E-33	323	108585	-2,31	1,24E-02	423	74214	-1,99	7,04E-20	523	104419	-1,77	8,44E-21
24	111750	-5,83	1,86E-299	124	76359	-3,74	6,99E-77	224	69115	-2,82	4,22E-44	324	58698	-2,29	1,40E-29	424	74158	-1,98	1,77E-61	524	76949	-1,77	3,40E-47
25	70365	-5,77	5,02E-108	125	5119	-3,70	1,18E-190	225	66935	-2,82	4,76E-56	325	31798	-2,29	1,13E-27	425	121498	-1,98	5,72E-06	525	80746	-1,77	4,08E-06
26	121136	-5,71	8,19E-222	126	77547	-3,70	8,69E-91	226	123084	-2,81	6,68E-35	326	123702	-2,28	7,21E-100	426	74818	-1,98	2,15E-40	526	23184	-1,77	3,19E-39
27	108784	-5,65	6,44E-28	127	5787	-3,66	3,69E-108	227	70090	-2,81	5,55E-04	327	109944	-2,28	3,99E-05	427	109850	-1,98	4,25E-41	527	44389	-1,76	2,30E-41
28	73250	-5,57	2,13E-47	128	61830	-3,66	1,34E-03	228	58472	-2,81	2,75E-07	328	108686	-2,28	7,10E-26	428	108160	-1,98	1,97E-06	528	123251	-1,75	1,90E-15
29	22331	-5,55	1,43E-235	129	110653	-3,66	3,39E-220	229	124198	-2,79	2,33E-09	329	54089	-2,27	1,56E-03	429	121990	-1,97	3,30E-31	529	104353	-1,75	6,79E-17
30	124043	-5,54	7,23E-05	130	52489	-3,65	3,64E-201	230	4517	-2,79	7,63E-10	330	79813	-2,27	1,44E-74	430	103039	-1,97	6,71E-13	530	64312	-1,75	8,50E-13
31	69904	-5,50	1,73E-96	131	80639	-3,63	5,34E-188	231	107524	-2,76	7,00E-92	331	69537	-2,27	9,09E-04	431	110862	-1,97	1,31E-15	531	123202	-1,75	9,40E-59
32	121416	-5,48	4,88E-175	132	32798	-3,63	5,22E-22	232	39587	-2,76	8,50E-04	332	122278	-2,27	1,85E-09	432	108676	-1,96	2,58E-09	532	122079	-1,75	1,41E-16
33	110127	-5,48	0,00E+00	133	43701	-3,61	1,59E-158	233	58356	-2,75	4,21E-187	333	106627	-2,27	5,13E-54	433	121868	-1,96	5,36E-36	533	124056	-1,75	3,16E-03
34	122824	-5,44	3,23E-56	134	56771	-3,61	3,32E-40	234	45138	-2,75	5,54E-104	334	121107	-2,26	1,76E-101	434	103671	-1,96	4,39E-19	534	36391	-1,74	6,08E-29
35	103062	-5,42	3,56E-113	135	69834	-3,61	5,05E-167	235	103012	-2,74	2,10E-41	335	60773	-2,26	5,79E-38	435	107667	-1,95	3,46E-03	535	34112	-1,74	2,65E-75
36	107641	-5,41	7,21E-04	136	76601	-3,59	6,30E-22	236	65522	-2,74	2,54E-63	336	26871	-2,25	7,54E-50	436	5710	-1,95	1,61E-59	536	23332	-1,74	3,40E-34
37	49274	-5,39	8,38E-163	137	80056	-3,58	4,32E-61	237	56236	-2,74	4,05E-02	337	52976	-2,25	1,73E-79	437	50215	-1,95	7,96E-55	537	107335	-1,74	8,52E-32
38	52315	-5,37	1,02E-185	138	58774	-3,58	1,17E-09	238	112493	-2,73	1,31E-03	338	72780	-2,25	5,88E-40	438	121801	-1,95	8,84E-82	538	104595	-1,74	2,09E-28
39	111239	-5,35	3,71E-08	139	105869	-3,58	7,42E-06	239	67026	-2,73	1,16E-66	339	56350	-2,24	1,23E-05	439	76833	-1,95	1,68E-54	539	22129	-1,74	1,51E-52
40	50793	-5,33	6,66E-43	140	109372	-3,55	1,94E-28	240	60493	-2,72	1,70E-02	340	78264	-2,24	1,37E-55	440	124296	-1,95	2,39E-06	540	73101	-1,74	7,34E-06
41	64049	-5,22	1,72E-45	141	67778	-3,55	1,60E-02	241	75506	-2,72	3,73E-68	341	120570	-2,24	9,74E-41	441	122076	-1,95	8,32E-45	541	66154	-1,73	4,77E-61
42	82041	-5,21	2,92E-99	142	47424	-3,52	1,78E-64	242	46285	-2,71	1,25E-23	342	111251	-2,23	6,14E-45	442	81964	-1,95	4,12E-06	542	49979	-1,73	5,67E-58
43	80086	-5,18	3,82E-114	143	121139	-3,50	8,73E-175	243	109945	-2,71	3,49E-72	343	68812	-2,22	6,99E-31	443	111451	-1,94	7,84E-10	543	62377	-1,72	2,65E-40
44	81576	-5,18	0,00E+00	144	120359	-3,49	3,87E-97	244	82095	-2,70	4,73E-61	344	105393	-2,22	1,31E-02	444	107202	-1,94	6,88E-09	544	120147	-1,72	5,52E-43
45	105156	-5,17	4,52E-12	145	106161	-3,48	1,41E-50	245	122084	-2,70	1,37E-32	345	76082	-2,20	1,05E-60	445	52248	-1,94	4,89E-33	545	120879	-1,72	1,54E-10
46	69555	-5,14	6,92E-04	146	110879	-3,48	8,22E-12	246	107071	-2,70	3,78E-02	346	59002	-2,20	1,16E-03	446	109081	-1,94	5,02E-09	546	111192	-1,72	1,71E-25
47	68831	-5,09	2,04E-42	147	2852	-3,45	7,89E-32	247	30776	-2,70	8,06E-03	347	41152	-2,20	5,71E-10	447	69652	-1,94	6,05E-19	547	123220	-1,71	7,99E-45
48	3094	-5,06	3,76E-88	148	104081	-3,43	2,83E-19	248	106686	-2,69	7,86E-67	348	59402	-2,20	8,68E-18	448	77969	-1,93	9,54E-29	548	123976	-1,71	1,22E-16
49	54461	-5,04	1,75E-87	149	60144	-3,42	2,17E-04	249	58584	-2,69	2,75E-128	349	80872	-2,20	1,08E-51	449	72581	-1,93	4,55E-09	549	80863	-1,71	1,20E-36
50	65410	-5,04	0,00E+00	150	58853	-3,42	3,16E-115	250	105931	-2,68	6,89E-04	350	78679	-2,18	2,29E-68	450	124149	-1,93	1,80E-52	550	55766	-1,71	2,59E-31
51	59876	-5,00	4,61E-64	151	82633	-3,41	1,54E-13	251	78387	-2,68	1,09E-113	351	59624	-2,18	4,35E-28	451	58299	-1,92	1,65E-24	551	110409	-1,71	2,88E-50
52	3488	-4,95	4,58E-65	152	105154	-3,38	2,47E-02	252	102607	-2,68	2,33E-85	352	48301	-2,18	4,62E-61	452	63774	-1,92	5,79E-22	552	107577	-1,71	5,22E-27
53	59655	-4,94	1,25E-06	153	108749	-3,38	1,11E-13	253	6005	-2,68	1,12E-50	353	77806	-2,18	2,97E-75	453	79817	-1,92	1,45E-41	553	105820	-1,71	8,01E-04
54	122820	-4,94	7,89E-04	154	57015	-3,35	1,47E-06	254	109370	-2,67	2,05E-02	354	112556	-2,18	6,87E-29	454	44449	-1,91	5,21E-59	554	109549	-1,70	3,04E-03
55	122795	-4,92	2,10E-153	155	72922	-3,35	5,01E-114	255	105445	-2,65	9,50E-14	355	105454	-2,17	1,10E-15	455	122124	-1,91	2,61E-58	555	122350	-1,70	2,39E-49
56	105444	-4,90	1,26E-268	156	123149	-3,34	1,26E-113	256	60419	-2,65	1,70E-05	356	80922	-2,17	5,25E-47	456	108783	-1,91	8,86E-16	556	68606	-1,70	1,52E-38
57	66766	-4,86	4,52E-11	157	58563	-3,33	1,48E-03	257	5135	-2,64	1,79E-76	357	106895	-2,17	3,97E-22	457	123633	-1,91	1,21E-51	557	108332	-1,70	2,02E-26
58	112491	-4,85	1,30E-06	158	27706	-3,33	9,89E-10	258	110316	-2,64	6,06E-64	358	65975	-2,17	2,86E-25	458	56434	-1,91	4,51E-30	558	50159	-1,69	2,53E-14
59	68371	-4,81	1,36E-54	159	59333	-3,31	2,84E-04	259	122993	-2,63	2,94E-20	359	79244	-2,17	3,06E-91	459	75335	-1,91	1,41E-34	559	60752	-1,69	4,62E-03
60	107229	-4,76	2,26E-03	160	58574	-3,26	2,94E-09	260	39637	-2,63	7,60E-53	360	2716	-2,16	2,10E-18	460	5233	-1,91	3,07E-42	560	23059	-1,69	3,96E-69
61	109344	-4,73	2,09E-02	161	61350	-3,26	6,09E-112	261	57632	-2,63	2,65E-82	361	42181	-2,16	9,91E-20	461	68997	-1,91	7,17E-07	561	52222	-1,69	2,92E-24
62	81082	-4,73	1,80E-157	162	105155	-3,25	7,06E-03	262	58117	-2,62	1,79E-125	362	109045	-2,16	3,32E-02	462	109206	-1,90	3,31E-09	562	66041	-1,69	1,013E-19
63	76238	-4,71	0,00E+00	163	123468	-3,23	5,19E-12	263	74187	-2,60	4,88E-14	363	77770	-2,14	1,30E-08	463	109187	-1,90	1,92E-33	563	121372	-1,69	1,555E-34
64	124084	-4,71	1,49E-50	164	78072	-3,23	1,38E-05	264	106606	-2,60	3,58E-24	364	43115	-2,14	1,29E-21	464	104097	-1,90	3,95E-09	564	107674	-1,69	2,147E-06
65	66345	-4,68	2,91E-280	165	77481	-3,22	2,58E-142	265	65315	-2,60	1,60E-42	365	69605	-2,14	5,84E-03	465	62225	-1,90	1,07E-37	565	58607	-1,68	4,818E-47
66	80879	-4,66	3,01E-171	166	110761	-3,21	6,26E-104	266	123539	-2,60	2,80E-18	366	122031	-2,13	5,75E-18	466	103222	-1,90	2,55E-69	566	104491	-1,68	1,27E-56
67	54886	-4,65	1,15E-02	167	58333	-3,20	4,08E-68	267	80867	-2,59	1,35E-94	367	120260	-2,13	7,18E-45	467	3823	-1,90	7,83E-50	567	111023	-1,68	1,397E-32
68	105805	-4,65	1,13E-02	168	105379	-3,20	3,08E-03	268	41501	-2,59	9,02E-16	368	22689	-2,13	1,12E-70	468	70527	-1,89	9,49E-05	568	53395	-1,68	2,182E-21
69	122825	-4,62	7,28E-97	169	80607	-3,16	4,80E-15	269	4561	-2,59	4,29E-07	369	121654	-2,13	5,54E-61	469	77557	-1,89	4,92E-25	569	121294	-1,68	1,035E-31
70	108781	-4,58	8,55E-162	170	122324	-3,16	3,16E-03	270	120362	-2,58	7,63E-93	370	81586	-2,13	5,45E-50	470	73523	-1,89	1,28E-71	570	102416	-1,68	9,943E-21
71	69901	-4,58	3,06E-04	171	110830	-3,15	1,84E-08	271	122941	-2,58	5,39E-97	371	66963	-2,12	3,94E-36	471	120568	-1,88	3,36E-83	571	4294	-1,68	2,034E-20
72	110457	-4,56	5,78E-124	172	110456	-3,13	4,65E-43	272	69742	-2,57	3,84E-08	372	105455	-2,12	1,82E-26	472	66865	-1,88	1,59E-12	572	49373	-1,68	4,147E-28
73	121534	-4,55	2,45E-127	173	57940	-3,13	4,33E-83	273	105533	-2,57	9,00E-39	373	111966	-2,11	5,07E-04	473	104050	-1,88	1,32E-02	573	1751	-1,67	2,201E-68
74	69696	-4,55	2,10E-10	174	79738	-3,11	3,31E-86	274	70491	-2,57	6,67E-05	374	122629	-2,11	2,65E-25	474	69141	-1,87	1,21E-65	574	80820	-1,67	2,252E-39
75	105894	-4,54	1,63E-02	175	47066	-3,11	4,24E-109	275	105465	-2,56	2,97E-15	375	51130	-2,11	1,37E-26	475	65743	-1,87	2,49E-10	575	58428	-1,67	7,86E-08
76	76763	-4,53	8,17E-15	176	70962	-3,10	1,58E-02	276	31075	-2,56	3,82E-53	376	72408	-2,11	7,93E-26	476	58456	-1,87	6,50E-04	576	51212	-1,67	5,622E-52
77	122506	-4,49	9,16E-183	177	75704	-3,09	1,32E-75	277	59746	-2,55	5,75E-08	377	121337	-2,10	2,04E-02	477	5742	-1,87	9,19E-29	577	77647	-1,67	4,94E-12
78	105291	-4,47	3,54E-81	178	76215	-3,09	1,04E-207	278	50212	-2,55	4,00E-121	378	123009	-2,10	1,63E-29	478	120877	-1,86	2,70E-68	578	112014	-1,67	0,0403123
79	23353	-4,43	4,93E-168	179	76682	-3,08	4,57E-02	279	105860	-2,55	1,33E-50	379	78956	-2,09	1,57E-50	479	69171	-1,86	9,92E-06	579	80748	-1,66	2,544E-34
80	62716	-4,43	1,76E-123	180	73536	-3,08	2,61E-110	280	5000	-2,55	4,83E-04	380	121706	-2,09	8,76E-56	480	75304	-1,86	2,22E-56	580	120864	-1,66	1,856E-43
81	6103	-4,39	7,37E-04	181	104106	-3,07	2,11E-02	281	102904	-2,54	1,55E-06	381	110382	-2,09	4,78E-03	481	21176	-1,86	5,64E-42	581	61605	-1,66	0,027016
82	59362	-4,38	6,37E-13	182	67509	-3,06	1,65E-11	282	121664	-2,54	2,66E-129	382	62359	-2,08	3,05E-02	482	124283	-1,86	6,58E-27	582	81690	-1,66	1,152E-37
83	102500	-4,36	2,17E-18	183	109373	-3,06	1,92E-28	283	122301	-2,54	2,50E-90	383	69879	-2,07	4,85E-92	483	109394	-1,85	2,28E-08	583	104222	-1,65	1,107E-22
84	3327	-4,30	3,07E-107	184	108583	-3,04	1,59E-139	284	60676	-2,51	5,98E-88	384	103822	-2,07	2,37E-31	484	75116	-1,85	4,59E-52	584	35109	-1,65	6,242E-05
85	69863	-4,30	1,58E-181	185	104219	-3,04	3,23E-60	285	66436	-2,51	2,51E-71	385	3579	-2,07	1,48E-29	485	45912	-1,85	1,73E-58	585	49038	-1,65	2,552E-18
86	110317	-4,28	2,78E-02	186	67109	-3,04	5,78E-23	286	106223	-2,50	3,21E-132	386	70520	-2,06	4,80E-34	486	58731	-1,85	2,30E-02	586	51103	-1,65	1,723E-55
87	105840	-4,26	3,69E-190	187	43101	-3,04	3,18E-10	287	121686	-2,50	1,88E-119	387	107073	-2,06	5,94E-23	487	107639	-1,84	6,22E-24	587	123999	-1,65	2,362E-22
88	68588	-4,24	2,20E-159	188	122074	-3,03	1,00E-109	288	5612	-2,50	1,20E-47	388	75105	-2,06	1,62E-21	488	64820	-1,84	1,71E-49	588	122879	-1,65	4,453E-30
89	104260	-4,21	1,44E-03	189	110711	-3,02	6,45E-13	289	63687	-2,48	2,81E-49	389	2036	-2,05	2,29E-18	489	106116	-1,84	1,25E-03	589	41149	-1,65	5,01E-23
90	23240	-4,19	1,56E-78	190	60847	-3,00	9,57E-89	290	108409	-2,46	1,07E-47	390	77795	-2,05	1,60E-56	490	104890	-1,84	5,39E-31	590	106626	-1,64	5,842E-34
91	105449	-4,18	1,17E-02	191	65583	-3,00	1,63E-13	291	123718	-2,46	4,45E-44	391	22013	-2,05	7,79E-45	491	102920	-1,84	7,77E-19	591	79545	-1,64	1,144E-51
92	79741	-4,17	1,10E-202	192	110762	-3,00	4,99E-28	292	69625	-2,46	1,57E-07	392	109846	-2,04	4,14E-06	492	110444	-1,84	6,87E-21	592	103189	-1,64	6,863E-08
93	122992	-4,15	3,91E-62	193	68813	-3,00	1,51E-85	293	38812	-2,45	2,81E-122	393	4654	-2,04	1,16E-50	493	21388	-1,83	1,12E-40	593	112001	-1,64	1,42E-12
94	105806	-4,13	4,80E-02	194	111912	-2,99	3,59E-66	294	124277	-2,45	4,86E-05	394	122233	-2,04	4,36E-48	494	67325	-1,83	2,95E-32	594	76169	-1,64	1,028E-50
95	111838	-4,12	2,08E-123	195	55172	-2,99	6,15E-90	295	76971	-2,44	5,86E-72	395	59991	-2,04	5,26E-28	495	53238	-1,83	3,56E-28	595	54865	-1,64	4,502E-12
96	105448	-4,11	4,30E-02	196	78401	-2,97	2,07E-166	296	121399	-2,43	4,76E-42	396	74943	-2,03	3,70E-56	496	76766	-1,83	1,01E-50	596	68279	-1,64	8,032E-13
97	120953	-4,08	4,32E-107	197	71029	-2,96	3,69E-68	297	59065	-2,43	5,34E-09	397	6108	-2,03	1,25E-19	497	105775	-1,83	4,72E-28	597	121214	-1,63	1,178E-34
98	105224	-4,07	2,50E-125	198	57088	-2,96	4,50E-75	298	64617	-2,42	6,01E-36	398	64869	-2,03	7,36E-05	498	81742	-1,83	1,63E-49	598	107297	-1,63	1,803E-08
99	76620	-4,06	0,00E+00	199	45343	-2,96	2,13E-29	299	44362	-2,41	2,76E-47	399	70830	-2,03	2,09E-24	499	122584	-1,83	2,43E-51	599	69035	-1,63	3,22E-23
100	4860	-4,06	2,04E-94	200	62463	-2,95	2,34E-58	300	60067	-2,41	9,47E-75	400	75247	-2,03	8,53E-03	500	124119	-1,83	1,47E-34	600	1702	-1,63	2,459E-49

Supplementary Table S11 (continued): FULL list of DEGs from RNA-Seq data for Δ*ngs1* (jgi|Trire2|79669) grown on GlcNAc and compared to control (WT grown on GlcNAc).

Differentially expressed genes (DEGs) were defined by fold change ≥|1.0| and a adjusted p-value (padj) of < 0.05 found by DESeq2 (RStudio 2022.07.2+576)

	Identifier (TRIREDRAFT)	log2fc	padj
601	39221	-1,63	1,32E-20
602	60300	-1,63	2,42E-29
603	5164	-1,63	4,63E-32
604	75609	-1,63	1,19E-77
605	110765	-1,63	6,23E-03
606	4514	-1,62	1,56E-14
607	73631	-1,62	2,18E-13
608	121766	-1,62	1,91E-30
609	67907	-1,62	3,07E-22
610	3856	-1,62	3,86E-21
611	121133	-1,62	4,83E-71
612	105011	-1,62	2,14E-32
613	123437	-1,62	1,76E-35
614	107187	-1,61	9,46E-25
615	123631	-1,61	1,33E-41
616	3001	-1,61	3,81E-34
617	76532	-1,61	6,15E-78
618	48170	-1,61	6,56E-67
619	68973	-1,61	8,34E-03
620	70311	-1,61	5,39E-37
621	104200	-1,61	2,61E-21
622	58753	-1,61	8,46E-21
623	78062	-1,60	4,56E-28
624	6015	-1,60	1,03E-22
625	122778	-1,60	9,11E-04
626	1927	-1,60	1,60E-17
627	62769	-1,60	4,43E-26
628	76247	-1,60	2,53E-38
629	59351	-1,60	3,67E-05
630	61279	-1,60	1,38E-27
631	123850	-1,59	1,61E-61
632	46545	-1,59	1,97E-40
633	79037	-1,59	1,25E-17
634	65225	-1,59	1,25E-18
635	5812	-1,58	7,19E-25
636	74913	-1,58	4,77E-18
637	4952	-1,58	1,23E-04
638	106039	-1,58	5,43E-61
639	106081	-1,57	2,91E-32
640	55183	-1,57	1,61E-17
641	23208	-1,57	2,49E-13
642	1959	-1,57	9,85E-07
643	66551	-1,57	2,89E-03
644	32261	-1,57	2,34E-21
645	74356	-1,57	3,15E-32
646	58227	-1,56	9,15E-04
647	120781	-1,56	1,30E-56
648	120646	-1,56	1,50E-41
649	59354	-1,56	1,21E-24
650	108953	-1,56	5,86E-14
651	104535	-1,56	3,90E-31
652	121904	-1,56	2,12E-75
653	21890	-1,56	1,13E-45
654	121818	-1,56	1,72E-45
655	121878	-1,56	2,46E-61
656	21170	-1,55	3,67E-52
657	60780	-1,55	1,88E-22
658	109833	-1,55	1,52E-20
659	59988	-1,54	1,84E-40
660	56291	-1,54	5,77E-12
661	22739	-1,54	8,06E-44
662	62198	-1,54	9,79E-20
663	120415	-1,54	5,77E-38
664	105167	-1,54	3,80E-03
665	51722	-1,54	1,66E-51
666	62100	-1,53	3,49E-18
667	53701	-1,53	9,17E-28
668	55029	-1,53	5,60E-19
669	59723	-1,53	4,11E-08
670	109243	-1,53	4,71E-03
671	57494	-1,53	3,27E-21
672	TREG000000002	-1,53	1,19E-09
673	53972	-1,53	9,38E-21
674	124110	-1,53	9,20E-69
675	68184	-1,53	4,95E-24
676	123779	-1,53	1,75E-05
677	47795	-1,53	6,09E-58
678	36373	-1,53	1,34E-26
679	3591	-1,52	6,51E-22
680	119530	-1,52	2,18E-11
681	46607	-1,52	1,93E-39
682	77656	-1,52	3,85E-51
683	57666	-1,52	1,12E-28
684	74374	-1,52	9,72E-40
685	78732	-1,52	2,53E-38
686	105866	-1,52	7,01E-26
687	121757	-1,52	1,20E-22
688	69838	-1,52	7,64E-32
689	122909	-1,51	6,50E-31
690	76633	-1,51	7,91E-20
691	122448	-1,51	1,96E-23
692	120779	-1,51	3,62E-35
693	119788	-1,51	1,15E-41
694	60086	-1,51	6,31E-13
695	102676	-1,51	7,66E-36
696	119568	-1,51	3,60E-47
697	4152	-1,51	1,28E-26
698	72259	-1,51	6,17E-10
699	62820	-1,50	2,83E-19
700	60758	-1,50	9,36E-10

	Identifier (TRIREDRAFT)	log2fc	padj
701	68924	-1,50	2,69E-14
702	29333	-1,50	2,56E-15
703	75646	-1,50	1,08E-26
704	120012	-1,50	6,94E-48
705	80211	-1,50	3,34E-42
706	21396	-1,50	9,24E-45
707	122001	-1,50	1,28E-64
708	76740	-1,50	6,71E-23
709	121232	-1,49	1,60E-33
710	3283	-1,49	1,22E-02
711	109828	-1,49	2,17E-16
712	103149	-1,49	2,02E-25
713	21453	-1,48	2,23E-28
714	81803	-1,48	1,26E-22
715	74480	-1,48	8,59E-45
716	107601	-1,48	5,75E-24
717	26029	-1,48	4,70E-25
718	111764	-1,48	7,91E-05
719	81362	-1,48	3,30E-16
720	68086	-1,48	8,02E-20
721	65191	-1,48	5,39E-30
722	73937	-1,48	9,28E-35
723	121278	-1,48	3,85E-48
724	70960	-1,47	1,24E-10
725	46244	-1,47	4,80E-23
726	108005	-1,47	5,26E-33
727	4040	-1,47	1,60E-09
728	66432	-1,47	7,59E-14
729	104359	-1,46	2,62E-07
730	72012	-1,46	3,53E-42
731	43662	-1,46	9,65E-36
732	66687	-1,46	4,17E-11
733	105191	-1,46	1,03E-16
734	120195	-1,46	2,70E-33
735	56196	-1,46	8,67E-08
736	120821	-1,46	1,00E-39
737	63378	-1,45	5,50E-29
738	112131	-1,45	1,97E-12
739	121003	-1,45	6,00E-36
740	42766	-1,45	1,27E-07
741	80268	-1,45	1,96E-14
742	62462	-1,45	4,61E-40
743	58815	-1,45	1,94E-46
744	60591	-1,45	2,83E-15
745	65685	-1,45	5,86E-18
746	23090	-1,44	5,38E-13
747	121111	-1,44	1,50E-05
748	46490	-1,44	6,61E-57
749	52924	-1,44	5,23E-21
750	42053	-1,44	4,73E-13
751	49898	-1,44	2,39E-12
752	58404	-1,44	1,26E-34
753	55660	-1,44	4,62E-21
754	21658	-1,44	1,09E-64
755	106480	-1,44	5,56E-05
756	78377	-1,44	1,11E-37
757	61595	-1,43	1,36E-51
758	60784	-1,43	6,32E-07
759	4244	-1,43	2,71E-14
760	122422	-1,43	4,05E-25
761	73840	-1,43	2,44E-42
762	76939	-1,43	6,81E-29
763	56206	-1,43	3,64E-22
764	123176	-1,43	6,90E-55
765	49770	-1,43	1,79E-20
766	53446	-1,43	7,57E-16
767	54667	-1,43	2,19E-23
768	62470	-1,42	1,75E-34
769	66803	-1,42	9,38E-11
770	54616	-1,42	1,77E-14
771	111663	-1,42	4,14E-24
772	107058	-1,42	1,26E-25
773	112288	-1,42	2,40E-03
774	56176	-1,42	6,98E-19
775	119626	-1,42	6,11E-42
776	106437	-1,42	6,27E-13
777	51426	-1,41	2,75E-32
778	108132	-1,41	5,53E-15
779	49112	-1,41	3,53E-19
780	72941	-1,41	1,36E-45
781	78233	-1,41	1,40E-37
782	103033	-1,41	2,81E-02
783	5536	-1,41	2,28E-20
784	79106	-1,40	1,03E-29
785	122811	-1,40	5,07E-28
786	63180	-1,40	2,34E-11
787	121741	-1,40	1,52E-60
788	75564	-1,40	3,26E-52
789	123204	-1,40	1,18E-29
790	71970	-1,40	3,49E-18
791	21398	-1,40	2,45E-18
792	112019	-1,40	4,15E-06
793	73594	-1,40	2,93E-30
794	46209	-1,39	1,19E-12
795	68909	-1,39	8,70E-52
796	82246	-1,39	5,96E-18
797	122036	-1,39	1,07E-55
798	74177	-1,39	1,77E-34
799	78970	-1,38	9,74E-12
800	74252	-1,38	9,60E-27

	Identifier (TRIREDRAFT)	log2fc	padj
801	75687	-1,38	4,39E-41
802	120267	-1,38	4,63E-19
803	43814	-1,38	2,79E-29
804	120928	-1,38	1,94E-04
805	54427	-1,38	8,58E-08
806	53153	-1,38	2,11E-08
807	4213	-1,38	3,06E-11
808	77906	-1,38	4,76E-42
809	42919	-1,38	5,26E-22
810	57185	-1,37	1,52E-03
811	52763	-1,37	3,91E-40
812	120571	-1,37	4,75E-22
813	73765	-1,37	2,62E-44
814	2185	-1,37	3,20E-13
815	58627	-1,37	1,57E-22
816	3501	-1,37	2,02E-21
817	78576	-1,36	7,73E-23
818	68728	-1,36	2,99E-14
819	43906	-1,36	9,63E-49
820	56835	-1,36	3,88E-03
821	79568	-1,36	1,76E-36
822	123753	-1,36	2,08E-28
823	122127	-1,36	5,88E-09
824	21663	-1,36	1,61E-18
825	41248	-1,36	4,77E-08
826	3394	-1,35	4,39E-22
827	2591	-1,35	6,45E-10
828	3007	-1,35	7,15E-16
829	58519	-1,35	9,69E-14
830	106798	-1,35	7,89E-21
831	62153	-1,35	8,81E-14
832	103143	-1,35	1,30E-02
833	65948	-1,35	2,38E-06
834	104968	-1,35	3,40E-21
835	110850	-1,35	1,78E-04
836	120892	-1,35	2,97E-27
837	79111	-1,34	9,66E-34
838	64065	-1,34	4,30E-17
839	107784	-1,34	3,68E-40
840	121837	-1,34	1,12E-50
841	108778	-1,34	3,15E-26
842	45736	-1,34	3,64E-51
843	78870	-1,34	6,05E-32
844	71363	-1,33	7,28E-19
845	79644	-1,33	1,09E-11
846	111807	-1,33	1,26E-07
847	109341	-1,33	5,75E-09
848	64778	-1,33	5,75E-14
849	120747	-1,33	8,08E-10
850	5064	-1,33	9,49E-25
851	71039	-1,33	4,91E-21
852	119864	-1,33	1,65E-26
853	61750	-1,33	4,99E-29
854	54535	-1,33	2,71E-22
855	65997	-1,33	2,80E-02
856	123145	-1,33	5,68E-46
857	59515	-1,32	2,99E-20
858	78695	-1,32	1,51E-38
859	70197	-1,32	1,01E-04
860	66210	-1,32	4,82E-09
861	42267	-1,32	1,26E-02
862	81713	-1,32	1,00E-42
863	23323	-1,32	7,99E-24
864	43242	-1,32	1,02E-06
865	122363	-1,32	5,01E-08
866	106452	-1,32	1,12E-11
867	109548	-1,32	2,05E-03
868	109015	-1,32	8,06E-26
869	60879	-1,32	2,90E-12
870	22845	-1,31	2,76E-14
871	112081	-1,31	1,05E-29
872	122505	-1,31	5,49E-11
873	72287	-1,31	6,68E-08
874	109296	-1,31	6,62E-05
875	63869	-1,31	2,29E-05
876	54198	-1,31	8,68E-24
877	74568	-1,31	1,76E-35
878	4981	-1,31	1,88E-21
879	27948	-1,30	5,75E-21
880	81460	-1,30	4,05E-10
881	104104	-1,30	4,53E-05
882	123673	-1,30	1,06E-10
883	73985	-1,30	3,68E-28
884	78162	-1,30	6,78E-23
885	110171	-1,30	3,84E-26
886	105647	-1,30	1,97E-11
887	102497	-1,30	1,45E-29
888	45503	-1,29	2,18E-11
889	62643	-1,29	4,51E-07
890	75414	-1,29	1,44E-25
891	71791	-1,29	4,85E-21
892	105515	-1,29	3,41E-03
893	1947	-1,29	1,21E-19
894	122587	-1,29	5,54E-36
895	81541	-1,29	2,50E-16
896	119845	-1,29	2,52E-11
897	120607	-1,28	3,11E-24
898	80578	-1,28	2,60E-05
899	74390	-1,28	1,40E-23
900	82516	-1,28	3,08E-22

	Identifier (TRIREDRAFT)	log2fc	padj
901	65406	-1,28	1,41E-26
902	63269	-1,28	4,99E-12
903	2583	-1,28	1,93E-13
904	123588	-1,28	4,16E-14
905	59833	-1,28	8,51E-14
906	59248	-1,28	2,39E-19
907	28050	-1,28	2,09E-22
908	106781	-1,27	5,04E-18
909	2648	-1,27	2,81E-30
910	21435	-1,27	4,40E-22
911	73621	-1,27	5,53E-07
912	120868	-1,27	1,36E-47
913	102593	-1,27	1,34E-26
914	124314	-1,27	7,42E-22
915	22386	-1,27	3,09E-02
916	109790	-1,27	9,81E-11
917	121471	-1,26	6,76E-31
918	121135	-1,26	2,92E-07
919	77227	-1,26	8,72E-11
920	31611	-1,26	5,24E-10
921	122040	-1,26	5,25E-28
922	21407	-1,26	2,74E-28
923	56467	-1,26	8,32E-03
924	53133	-1,26	1,68E-18
925	77288	-1,26	5,69E-06
926	3596	-1,26	2,00E-02
927	81756	-1,26	1,79E-15
928	122141	-1,26	2,61E-47
929	55252	-1,26	1,63E-21
930	121906	-1,26	7,29E-39
931	30274	-1,26	3,85E-10
932	51499	-1,25	4,55E-32
933	123476	-1,25	1,70E-18
934	121336	-1,25	1,52E-11
935	61965	-1,25	3,75E-40
936	81001	-1,25	4,97E-19
937	4117	-1,25	3,76E-14
938	122416	-1,25	3,11E-10
939	63558	-1,25	4,80E-11
940	122176	-1,25	4,48E-28
941	107123	-1,24	7,51E-17
942	58466	-1,24	2,57E-03
943	120020	-1,24	1,39E-28
944	55508	-1,24	5,21E-11
945	74987	-1,24	7,14E-20
946	77119	-1,24	9,44E-24
947	65672	-1,24	5,98E-31
948	70204	-1,24	2,66E-02
949	53529	-1,24	1,24E-22
950	122724	-1,24	4,97E-12
951	122457	-1,24	4,03E-36
952	124202	-1,23	7,78E-22
953	119609	-1,23	4,50E-30
954	80025	-1,23	8,74E-43
955	120978	-1,23	3,03E-15
956	122870	-1,23	5,46E-07
957	45512	-1,22	5,02E-15
958	120540	-1,22	2,51E-22
959	102778	-1,22	1,99E-02
960	27085	-1,22	4,86E-23
961	102837	-1,22	7,01E-16
962	64420	-1,22	1,68E-23
963	60346	-1,22	4,26E-12
964	121126	-1,22	4,21E-19
965	112567	-1,22	1,36E-09
966	62213	-1,22	9,95E-31
967	80890	-1,22	1,59E-13
968	33692	-1,21	2,62E-33
969	75271	-1,21	2,55E-03
970	124113	-1,21	9,08E-18
971	78688	-1,21	8,77E-27
972	73560	-1,21	3,25E-16
973	58790	-1,21	5,23E-31
974	67882	-1,21	6,24E-17
975	74379	-1,21	8,13E-12
976	122794	-1,21	2,97E-28
977	111818	-1,21	2,42E-02
978	78409	-1,21	2,78E-29
979	111787	-1,21	1,56E-20
980	1673	-1,21	8,55E-10
981	112146	-1,20	5,48E-09
982	22667	-1,20	1,25E-18
983	108183	-1,20	9,21E-09
984	103506	-1,20	9,79E-24
985	53669	-1,20	3,97E-14
986	67840	-1,20	3,64E-12
987	111564	-1,20	9,18E-14
988	107031	-1,20	2,33E-03
989	39942	-1,20	5,08E-25
990	71783	-1,20	8,32E-21
991	58767	-1,20	4,19E-02
992	3568	-1,20	2,31E-03
993	74774	-1,20	2,77E-38
994	111682	-1,20	5,38E-05
995	124256	-1,20	2,29E-31
996	120752	-1,20	2,49E-19
997	102855	-1,19	2,52E-07
998	34297	-1,19	7,25E-06
999	2100	-1,19	8,33E-19
1000	74346	-1,19	6,78E-26

	Identifier (TRIREDRAFT)	log2fc	padj
1001	109946	-1,19	1,34E-02
1002	120872	-1,19	1,60E-21
1003	60855	-1,19	1,08E-15
1004	66562	-1,19	3,10E-20
1005	106314	-1,19	1,29E-11
1006	105518	-1,19	4,54E-13
1007	106160	-1,18	2,17E-08
1008	80515	-1,18	1,08E-15
1009	112286	-1,18	7,75E-05
1010	58396	-1,18	1,96E-03
1011	79059	-1,18	4,54E-35
1012	65496	-1,18	3,88E-25
1013	120621	-1,18	9,04E-30
1014	63503	-1,18	1,88E-14
1015	119855	-1,18	2,09E-22
1016	74486	-1,18	1,19E-25
1017	107857	-1,17	2,47E-14
1018	4064	-1,17	4,07E-22
1019	122659	-1,17	7,88E-25
1020	68022	-1,17	6,77E-15
1021	2087	-1,17	2,02E-26
1022	64375	-1,17	1,56E-16
1023	61576	-1,17	4,02E-15
1024	109249	-1,17	1,44E-02
1025	81896	-1,17	2,63E-22
1026	68444	-1,17	1,07E-09
1027	81906	-1,17	2,31E-26
1028	67504	-1,17	1,62E-04
1029	110655	-1,16	4,28E-13
1030	66092	-1,16	1,91E-06
1031	44434	-1,16	1,22E-29
1032	23327	-1,16	9,08E-30
1033	55123	-1,16	1,83E-18
1034	103112	-1,16	3,39E-07
1035	112384	-1,16	5,43E-13
1036	65433	-1,16	8,87E-13
1037	78340	-1,16	1,75E-07
1038	104807	-1,16	2,33E-19
1039	111053	-1,16	1,55E-07
1040	4442	-1,16	7,69E-04
1041	104744	-1,16	1,30E-14
1042	59597	-1,16	3,71E-02
1043	122287	-1,15	2,00E-13
1044	110363	-1,15	3,22E-02
1045	22705	-1,15	7,65E-11
1046	47136	-1,15	7,80E-22
1047	3333	-1,15	6,64E-05
1048	60255	-1,15	2,91E-31
1049	123396	-1,15	3,63E-02
1050	109648	-1,15	4,74E-02
1051	111179	-1,15	2,25E-03
1052	121702	-1,15	3,39E-02
1053	82667	-1,15	1,25E-19
1054	123131	-1,14	3,19E-06
1055	55790	-1,14	1,20E-22
1056	1993	-1,14	1,78E-15
1057	62244	-1,14	1,07E-07
1058	111893	-1,14	8,67E-09
1059	112596	-1,14	4,71E-21
1060	112525	-1,14	8,48E-09
1061	70991	-1,14	1,18E-04
1062	21363	-1,14	3,20E-17
1063	49205	-1,14	4,91E-14
1064	78415	-1,14	1,41E-14
1065	22611	-1,14	2,32E-22
1066	78044	-1,14	1,11E-15
1067	122376	-1,14	5,65E-19
1068	120354	-1,13	3,61E-24
1069	62059	-1,13	4,21E-16
1070	123382	-1,13	1,13E-24
1071	76677	-1,13	5,48E-18
1072	49649	-1,12	1,93E-25
1073	107850	-1,12	7,47E-06
1074	73571	-1,12	6,05E-28
1075	38833	-1,12	1,03E-08
1076	121579	-1,12	1,02E-16
1077	79495	-1,12	1,11E-23
1078	80484	-1,12	3,08E-36
1079	61550	-1,12	5,68E-15
1080	21406	-1,12	4,10E-32
1081	52267	-1,12	2,66E-07
1082	63419	-1,11	3,43E-17
1083	79287	-1,11	3,98E-27
1084	54596	-1,11	1,63E-10
1085	74162	-1,11	6,45E-11
1086	81389	-1,11	3,87E-12
1087	106287	-1,11	1,06E-16
1088	75514	-1,10	8,98E-20
1089	65588	-1,10	4,42E-02
1090	21595	-1,10	3,92E-06
1091	73842	-1,10	5,34E-24
1092	120610	-1,10	3,90E-14
1093	1925	-1,10	2,28E-22
1094	5970	-1,10	5,36E-07
1095	76744	-1,10	6,86E-20
1096	48883	-1,10	1,55E-16
1097	105984	-1,09	2,04E-11
1098	43418	-1,09	5,08E-16
1099	42249	-1,09	1,23E-22
1100	66895	-1,09	3,94E-15

	Identifier (TRIREDRAFT)	log2fc	padj
1101	119554	-1,09	2,20E-15
1102	102744	-1,09	2,16E-17
1103	64959	-1,09	2,28E-02
1104	121773	-1,09	1,17E-09
1105	66161	-1,09	3,98E-18
1106	79089	-1,09	1,10E-18
1107	63157	-1,09	9,93E-17
1108	112281	-1,09	1,41E-02
1109	110295	-1,09	1,84E-12
1110	81570	-1,09	2,76E-15
1111	61153	-1,09	2,81E-13
1112	2912	-1,09	6,45E-21
1113	106014	-1,09	8,12E-10
1114	1777	-1,08	2,74E-10
1115	103162	-1,08	6,12E-07
1116	104251	-1,08	1,28E-03
1117	55552	-1,08	1,66E-19
1118	65819	-1,08	8,67E-05
1119	108827	-1,08	2,24E-21
1120	111567	-1,08	6,56E-03
1121	5776	-1,08	1,28E-20
1122	67493	-1,08	8,77E-11
1123	82613	-1,08	2,54E-16
1124	55105	-1,08	3,77E-02
1125	79565	-1,08	2,17E-10
1126	67616	-1,07	2,04E-21
1127	47221	-1,07	3,20E-21
1128	81139	-1,07	2,10E-11
1129	46702	-1,07	2,63E-22
1130	73873	-1,07	1,01E-25
1131	105613	-1,07	7,38E-13
1132	123636	-1,07	4,68E-19
1133	71304	-1,07	6,90E-14
1134	108775	-1,07	7,69E-09
1135	110948	-1,06	3,99E-11
1136	104261	-1,06	1,48E-03
1137	111307	-1,06	6,48E-12
1138	55240	-1,06	2,36E-03
1139	60338	-1,06	7,52E-20
1140	110152	-1,06	9,84E-10
1141	62335	-1,06	6,98E-20
1142	69131	-1,06	5,00E-08
1143	58867	-1,06	6,14E-04
1144	109536	-1,06	4,79E-18
1145	77138	-1,06	7,34E-20
1146	77495	-1,06	3,21E-26
1147	80115	-1,06	4,93E-11
1148	54036	-1,06	3,67E-02
1149	22143	-1,06	3,61E-17
1150	75169	-1,06	1,27E-20
1151	123777	-1,06	1,33E-09
1152	120833	-1,06	4,91E-27
1153	4526	-1,06	8,91E-08
1154	3891	-1,05	1,69E-08
1155	48281	-1,05	1,88E-20
1156	56344	-1,05	4,59E-12
1157	75921	-1,05	5,42E-14
1158	5377	-1,05	3,73E-11
1159	121178	-1,05	1,30E-10
1160	111170	-1,05	1,37E-02
1161	120363	-1,05	5,18E-07
1162	61815	-1,04	6,86E-19
1163	124010	-1,04	2,87E-26
1164	55589	-1,04	6,68E-16
1165	2186	-1,04	1,19E-17
1166	121605	-1,04	1,18E-14
1167	51430	-1,04	1,77E-30
1168	103138	-1,04	4,3E-15
1169	73450	-1,04	4,13E-25
1170	105589	-1,04	1,54E-19
1171	60392	-1,04	9,38E-08
1172	119555	-1,04	5,32E-21
1173	57291	-1,04	5,65E-11
1174	58539	-1,04	2,59E-15
1175	43615	-1,04	1,96E-28
1176	107197	-1,04	2,78E-06
1177	120081	-1,03	4,43E-23
1178	59023	-1,03	1,09E-05
1179	119603	-1,03	6,21E-22
1180	22799	-1,03	5,43E-13
1181	79946	-1,03	7,95E-13
1182	123191	-1,03	8,52E-16
1183	78320	-1,03	1,54E-22
1184	111074	-1,03	1,7E-18
1185	75923	-1,02	8,91E-18
1186	121405	-1,02	3,09E-11
1187	104132	-1,02	0,00062
1188	121834	-1,02	1,11E-15
1189	77284	-1,02	3,85E-13
1190	81089	-1,02	0,020505
1191	54722	-1,02	6,46E-08
1192	75992	-1,02	1,31E-09
1193	69663	-1,02	9,32E-24
1194	121146	-1,02	9,61E-12
1195	38603	-1,02	2,02E-10
1196	2348	-1,02	4,48E-11
1197	109905	-1,01	7,71E-13
1198	108398	-1,01	7,4E-10
1199	66437	-1,01	1,68E-05
1200	62389	-1,01	1,87E-11

Supplementary Table S11 (continued): FULL list of DEGs from RNA-Seq data for Δ*ngs1* (jgi|Trire2|79669) grown on GlcNAc and compared to control (WT grown on GlcNAc).

Differentially expressed genes (DEGs) were defined by fold change ≥|1.0| and a adjusted p-value (padj) of < 0.05 found by DESeq2 (RStudio 2022.07.2+576)

	Identifier (TRIREDRAFT)	log2fc	padj
1201	107793	-1,01	3,01E-07
1202	103132	-1,01	5,82E-08
1203	120272	-1,01	5,64E-07
1204	79947	-1,01	1,94E-16
1205	112328	-1,01	5,33E-10
1206	112018	-1,01	5,69E-06
1207	46958	-1,01	5,12E-26
1208	72615	-1,01	3,00E-24
1209	56396	-1,00	1,26E-12
1210	102970	-1,00	2,27E-02

Supplementary Table S11 (continued): FULL list of DEGs from RNA-Seq data for Δ*ngs1* (jgi|Trire2|79669) grown on GlcNAc and compared to control (WT grown on GlcNAc).

Differentially expressed genes (DEGs) were defined by fold change ≥|1.0| and a adjusted p-value (padj) of < 0.05 found by DESeq2 (RStudio 2022.07.2+576)

	Identifier (TRIREDRAFT)	log2fc	padj		Identifier (TRIREDRAFT)	log2fc	padj		Identifier (TRIREDRAFT)	log2fc	padj		Identifier (TRIREDRAFT)	log2fc	padj		Identifier (TRIREDRAFT)	log2fc	padj		Identifier (TRIREDRAFT)	log2fc	padj
1	112568	10,42	2,50E-17	101	122087	4,36	2,23E-89	201	57914	3,27	6,89E-173	301	105816	2,66	1,22E-15	401	30166	2,22	1,31E-17	501	110214	1,87	6,32E-34
2	44278	10,28	0	102	110757	4,35	5,90E-19	202	38640	3,26	4,24E-186	302	103614	2,66	2,62E-08	402	79271	2,22	2,02E-23	502	66175	1,86	2,09E-14
3	103136	9,80	2,76E-15	103	60635	4,34	2,45E-12	203	31134	3,26	6,79E-78	303	110330	2,65	1,78E-02	403	110261	2,21	2,61E-03	503	41208	1,86	1,23E-21
4	81087	9,25	3,48E-133	104	61055	4,32	1,44E-53	204	103129	3,25	1,69E-03	304	106885	2,64	1,73E-48	404	59322	2,21	2,12E-07	504	103470	1,86	3,29E-02
5	121441	9,06	1,05E-53	105	54227	4,32	2,83E-02	205	122614	3,24	1,20E-139	305	3716	2,64	3,98E-62	405	60889	2,21	2,61E-13	505	112085	1,86	1,45E-07
6	109361	8,97	3,10E-12	106	109811	4,29	6,91E-114	206	109234	3,23	3,45E-86	306	105260	2,63	4,25E-23	406	119823	2,21	1,09E-34	506	58511	1,86	2,76E-31
7	59689	8,83	2,81E-13	107	107340	4,27	0,00E+00	207	112031	3,21	2,92E-34	307	76155	2,62	1,54E-85	407	109746	2,20	7,62E-59	507	46266	1,86	2,34E-22
8	105279	8,23	7,99E-11	108	68019	4,26	2,43E-66	208	111897	3,21	5,56E-12	308	4124	2,61	1,50E-06	408	67579	2,20	8,23E-19	508	54239	1,86	7,62E-41
9	56289	8,08	2,06E-10	109	72526	4,23	9,60E-41	209	70021	3,20	5,39E-07	309	66751	2,61	1,10E-02	409	27395	2,19	8,94E-42	509	5270	1,86	4,52E-21
10	123234	7,99	1,80E-29	110	110848	4,23	2,98E-02	210	58857	3,19	1,56E-26	310	53961	2,61	5,46E-22	410	103108	2,19	3,60E-47	510	22785	1,85	3,31E-61
11	81275	7,92	8,26E-77	111	30758	4,22	8,09E-03	211	112126	3,18	1,71E-99	311	48211	2,61	6,15E-08	411	64937	2,19	1,57E-08	511	56064	1,85	3,31E-37
12	111915	7,85	2,64E-129	112	65037	4,21	2,87E-02	212	123095	3,18	6,96E-47	312	121308	2,61	9,59E-75	412	62053	2,19	8,34E-16	512	66795	1,84	7,62E-14
13	107867	7,36	2,03E-08	113	67639	4,21	3,15E-27	213	31248	3,17	3,20E-181	313	4726	2,60	5,61E-152	413	80149	2,17	8,54E-58	513	108885	1,84	1,02E-35
14	70800	7,28	4,84E-08	114	107960	4,20	1,51E-03	214	110855	3,17	6,79E-18	314	103275	2,60	2,61E-40	414	109146	2,17	8,47E-09	514	23408	1,84	4,37E-25
15	81536	7,16	4,50E-11	115	50104	4,19	1,30E-12	215	120784	3,17	3,22E-53	315	106181	2,59	2,11E-46	415	123473	2,17	4,55E-45	515	52073	1,84	9,03E-53
16	69736	7,13	6,45E-11	116	55802	4,17	4,82E-181	216	5337	3,17	2,68E-34	316	124222	2,57	3,37E-59	416	122374	2,17	6,74E-34	516	123199	1,84	2,60E-13
17	123550	7,10	1,53E-44	117	110471	4,16	2,15E-31	217	120473	3,16	8,94E-03	317	67377	2,57	7,54E-75	417	69557	2,16	4,55E-64	517	103393	1,83	1,01E-02
18	111932	6,88	1,12E-22	118	59391	4,16	4,55E-92	218	74198	3,15	1,78E-43	318	55887	2,56	4,93E-60	418	57647	2,16	1,71E-24	518	111955	1,83	1,17E-08
19	46794	6,87	0,00E+00	119	50323	4,15	2,64E-07	219	48747	3,15	3,80E-95	319	122963	2,56	3,51E-38	419	47432	2,16	8,67E-40	519	119931	1,83	6,96E-52
20	122495	6,82	7,56E-07	120	72627	4,10	8,94E-68	220	70334	3,14	5,52E-84	320	64656	2,56	1,44E-45	420	61127	2,15	4,03E-26	520	82626	1,83	7,69E-16
21	112128	6,72	4,65E-09	121	79921	4,10	1,88E-97	221	57857	3,14	1,24E-70	321	123818	2,55	2,75E-04	421	58802	2,14	1,18E-02	521	2322	1,83	1,33E-11
22	111138	6,71	1,12E-179	122	111527	4,09	2,08E-61	222	62611	3,12	7,29E-135	322	103131	2,55	4,81E-05	422	21873	2,13	1,80E-29	522	68990	1,83	1,25E-14
23	107869	6,65	1,91E-22	123	121127	4,09	2,13E-26	223	122198	3,10	3,40E-49	323	61114	2,55	4,21E-62	423	64720	2,13	8,86E-04	523	30084	1,83	3,48E-13
24	120837	6,63	0,00E+00	124	110220	4,07	4,05E-37	224	82619	3,10	1,87E-125	324	55636	2,54	2,65E-83	424	110276	2,13	5,41E-04	524	22510	1,82	8,66E-73
25	108349	6,62	1,44E-06	125	3717	4,02	2,10E-14	225	104073	3,10	3,82E-05	325	69245	2,54	2,22E-47	425	4027	2,12	2,67E-05	525	57592	1,82	1,90E-27
26	66819	6,62	5,06E-207	126	65986	4,01	3,97E-72	226	64906	3,10	1,17E-02	326	74453	2,54	1,04E-05	426	111923	2,12	1,09E-04	526	65950	1,82	1,08E-22
27	72632	6,60	3,71E-116	127	2223	4,00	3,34E-96	227	107669	3,08	4,94E-66	327	121306	2,53	8,87E-70	427	64667	2,12	3,85E-27	527	62576	1,81	1,44E-03
28	124051	6,53	7,51E-72	128	68705	3,99	2,50E-168	228	72379	3,07	1,72E-10	328	76696	2,53	3,00E-72	428	3449	2,12	1,24E-45	528	70932	1,81	2,12E-33
29	103451	6,48	0,00E+00	129	103695	3,96	1,16E-45	229	5107	3,06	6,46E-05	329	57555	2,53	2,12E-08	429	70956	2,11	5,75E-08	529	121156	1,81	2,79E-30
30	109235	6,42	7,68E-142	130	69857	3,95	6,89E-33	230	55709	3,06	8,33E-93	330	109779	2,51	1,15E-02	430	65141	2,11	8,59E-27	530	119576	1,81	6,85E-35
31	80659	6,37	8,22E-74	131	68230	3,95	5,26E-167	231	56470	3,04	2,80E-108	331	121743	2,51	3,30E-68	431	54972	2,11	1,13E-04	531	72567	1,81	4,84E-06
32	53029	6,35	9,09E-06	132	58848	3,94	2,63E-04	232	76227	3,02	1,00E-117	332	110440	2,47	1,43E-34	432	56625	2,11	3,39E-04	532	106625	1,81	2,37E-06
33	119790	6,30	8,84E-196	133	111236	3,94	1,27E-61	233	104215	3,02	1,09E-15	333	56684	2,47	5,89E-10	433	109311	2,11	1,06E-04	533	106130	1,81	5,74E-59
34	107868	6,27	1,10E-05	134	56328	3,93	3,18E-07	234	60374	3,01	3,08E-50	334	82039	2,47	4,18E-54	434	65198	2,11	5,21E-07	534	72076	1,81	3,51E-17
35	120176	6,27	0,00E+00	135	69811	3,92	1,33E-129	235	60988	3,01	4,18E-21	335	109673	2,47	1,77E-16	435	123914	2,10	7,74E-47	535	66583	1,81	4,38E-19
36	23415	6,27	0,00E+00	136	64181	3,90	3,33E-04	236	107279	3,00	2,49E-62	336	66598	2,47	1,04E-02	436	123955	2,10	2,75E-28	536	59801	1,80	6,02E-22
37	73897	6,25	2,45E-16	137	81430	3,89	4,03E-177	237	67035	2,99	1,12E-68	337	120688	2,46	1,98E-75	437	123865	2,09	5,21E-39	537	5275	1,80	2,93E-04
38	33387	6,24	1,38E-119	138	56726	3,88	2,11E-03	238	73110	2,99	3,33E-06	338	106706	2,46	1,60E-61	438	69972	2,09	1,82E-26	538	60187	1,80	1,45E-03
39	123978	6,22	0,00E+00	139	54226	3,85	1,98E-89	239	72524	2,98	8,04E-18	339	75027	2,46	2,24E-55	439	21422	2,09	1,13E-56	539	66592	1,80	1,92E-05
40	51365	6,16	0,00E+00	140	59952	3,84	4,77E-68	240	103059	2,97	1,61E-03	340	49589	2,45	1,87E-110	440	56003	2,08	5,30E-30	540	70092	1,80	8,88E-18
41	68427	6,13	4,24E-184	141	56996	3,83	5,49E-03	241	122745	2,96	1,58E-02	341	82208	2,45	4,38E-10	441	58952	2,08	1,21E-03	541	107947	1,79	5,00E-52
42	121164	6,04	0,00E+00	142	70933	3,82	2,72E-20	242	111094	2,96	1,01E-06	342	5502	2,44	2,84E-04	442	59771	2,08	1,70E-03	542	112134	1,79	1,08E-14
43	56646	6,01	2,04E-07	143	123261	3,82	2,58E-07	243	63526	2,94	2,58E-39	343	66611	2,44	1,09E-04	443	53611	2,08	7,44E-09	543	79405	1,79	5,63E-50
44	74563	5,96	4,02E-240	144	104322	3,81	3,73E-236	244	23062	2,94	9,57E-77	344	80340	2,42	3,37E-49	444	69066	2,08	7,04E-09	544	65229	1,79	1,05E-36
45	55881	5,92	8,13E-48	145	59388	3,79	4,24E-30	245	80875	2,94	4,86E-97	345	103335	2,42	1,89E-02	445	81522	2,07	2,81E-05	545	111446	1,79	1,90E-40
46	111890	5,83	2,85E-05	146	104277	3,79	1,78E-12	246	103886	2,94	1,23E-15	346	59642	2,41	6,28E-71	446	110455	2,07	3,89E-09	546	104549	1,78	7,84E-43
47	59843	5,80	1,79E-76	147	111442	3,78	6,08E-116	247	65055	2,93	1,20E-99	347	121968	2,40	1,68E-30	447	51415	2,07	7,75E-27	547	78552	1,78	2,50E-29
48	121495	5,76	2,46E-20	148	107494	3,77	3,35E-98	248	4941	2,93	4,38E-144	348	60445	2,39	4,52E-03	448	27181	2,06	5,05E-27	548	65164	1,78	1,09E-19
49	106537	5,74	4,81E-05	149	123029	3,75	2,18E-167	249	104084	2,92	1,43E-40	349	111145	2,39	4,77E-02	449	79202	2,06	4,06E-14	549	57370	1,78	1,33E-34
50	53079	5,67	1,03E-207	150	103799	3,74	2,48E-99	250	81843	2,92	7,27E-47	350	49753	2,39	2,68E-45	450	121717	2,04	4,72E-30	550	36703	1,77	4,50E-14
51	67692	5,67	2,01E-07	151	72183	3,72	2,31E-85	251	29993	2,90	4,63E-67	351	69468	2,39	3,57E-33	451	78828	2,03	2,28E-22	551	108201	1,77	4,12E-26
52	111729	5,67	1,10E-06	152	76065	3,71	8,92E-115	252	119860	2,90	2,20E-57	352	62172	2,38	9,82E-22	452	64345	2,03	1,94E-02	552	108157	1,77	5,32E-25
53	120017	5,59	5,38E-300	153	59382	3,71	2,47E-234	253	58701	2,89	7,55E-03	353	119789	2,38	6,86E-92	453	54166	2,03	6,43E-29	553	45717	1,77	1,36E-41
54	34493	5,49	4,48E-46	154	103458	3,70	2,58E-06	254	49946	2,89	3,42E-92	354	106879	2,38	6,62E-32	454	78713	2,03	9,78E-49	554	108835	1,76	1,88E-24
55	103423	5,34	6,43E-04	155	60329	3,70	3,02E-27	255	79726	2,87	2,59E-20	355	54954	2,38	8,07E-33	455	122736	2,02	9,79E-06	555	58068	1,76	7,18E-32
56	109122	5,32	2,54E-20	156	119552	3,70	3,13E-08	256	111395	2,86	3,79E-76	356	61536	2,37	9,01E-05	456	68254	2,02	5,96E-49	556	65735	1,76	1,34E-14
57	123274	5,31	4,20E-35	157	80058	3,69	4,51E-06	257	122007	2,85	1,21E-60	357	5924	2,37	2,87E-63	457	55990	2,02	2,26E-30	557	103668	1,76	3,65E-20
58	105150	5,26	2,41E-04	158	65915	3,68	1,35E-181	258	22912	2,84	1,35E-108	358	105027	2,37	1,71E-07	458	109078	2,02	7,78E-46	558	72091	1,76	2,61E-38
59	121883	5,25	3,77E-49	159	76852	3,65	7,66E-11	259	106697	2,84	1,10E-02	359	3363	2,37	1,64E-39	459	45445	2,01	2,01E-43	559	81553	1,76	2,55E-26
60	108671	5,17	3,30E-04	160	121482	3,64	1,85E-175	260	50616	2,83	3,64E-112	360	60370	2,36	4,43E-05	460	65739	2,00	4,41E-04	560	104394	1,75	2,53E-02
61	57643	5,10	1,18E-84	161	123510	3,62	8,09E-55	261	122582	2,82	2,65E-74	361	106538	2,36	2,43E-24	461	124169	2,00	7,78E-79	561	103813	1,75	2,44E-17
62	106129	5,09	5,23E-155	162	57179	3,60	4,56E-04	262	105269	2,81	2,65E-31	362	81517	2,36	2,14E-87	462	70961	1,99	5,16E-07	562	35777	1,75	4,35E-38
63	60418	5,08	5,59E-12	163	124115	3,60	9,05E-105	263	54962	2,81	6,57E-16	363	105763	2,36	1,52E-50	463	57671	1,99	8,86E-48	563	105771	1,74	7,24E-30
64	62872	5,08	1,58E-142	164	111357	3,59	6,86E-78	264	109335	2,81	8,66E-20	364	121735	2,35	3,50E-10	464	64784	1,99	3,62E-72	564	70172	1,74	9,8E-07
65	58823	5,07	1,93E-03	165	108145	3,59	3,32E-36	265	60085	2,81	8,76E-14	365	112094	2,35	3,35E-27	465	104333	1,99	2,25E-23	565	120120	1,74	1,44E-61
66	77093	5,05	7,44E-28	166	120698	3,58	2,18E-191	266	81778	2,81	1,04E-91	366	78683	2,35	9,39E-38	466	65741	1,98	1,54E-28	566	55561	1,74	2,29E-27
67	76897	5,02	3,50E-292	167	56687	3,53	1,05E-05	267	110709	2,81	7,42E-04	367	110493	2,34	5,10E-13	467	80096	1,98	4,73E-51	567	54550	1,74	1,82E-21
68	60116	4,98	1,31E-103	168	123639	3,51	2,89E-87	268	122108	2,80	1,19E-10	368	61212	2,34	1,17E-04	468	107644	1,98	4,86E-03	568	119956	1,74	2,97E-65
69	70341	4,98	2,29E-20	169	80645	3,51	9,42E-14	269	79606	2,80	6,99E-153	369	48080	2,34	5,27E-76	469	56432	1,98	3,31E-45	569	34252	1,74	5,56E-10
70	56314	4,98	1,34E-49	170	105765	3,50	2,46E-61	270	65162	2,79	8,27E-35	370	102966	2,33	2,79E-33	470	63914	1,97	4,50E-21	570	112230	1,73	9,46E-12
71	54219	4,94	3,16E-03	171	69881	3,50	4,97E-02	271	72339	2,78	3,74E-71	371	38080	2,33	3,52E-26	471	108540	1,96	9,61E-06	571	107137	1,73	2,26E-24
72	67541	4,94	7,21E-10	172	123226	3,49	4,44E-138	272	67084	2,78	3,92E-11	372	121396	2,33	5,60E-25	472	81668	1,96	2,14E-22	572	53777	1,73	7,18E-09
73	111405	4,88	1,12E-06	173	70967	3,49	1,36E-02	273	72611	2,77	3,65E-101	373	103801	2,32	4,16E-05	473	47603	1,96	2,32E-05	573	5347	1,73	1,37E-06
74	112115	4,85	5,59E-14	174	122556	3,48	9,70E-07	274	112247	2,77	2,70E-08	374	65215	2,32	7,21E-57	474	62439	1,96	1,56E-73	574	123283	1,73	4,12E-09
75	42152	4,85	4,75E-10	175	68508	3,47	3,38E-16	275	71080	2,75	3,13E-59	375	21412	2,32	7,43E-88	475	56095	1,95	7,35E-06	575	76734	1,72	2,63E-28
76	67752	4,68	0,00E+00	176	47286	3,46	1,56E-134	276	109538	2,75	3,20E-02	376	124177	2,31	1,40E-25	476	53428	1,95	7,67E-38	576	67494	1,72	2,13E-12
77	103009	4,66	3,39E-11	177	66753	3,46	2,85E-06	277	111129	2,75	5,69E-91	377	104606	2,31	9,70E-43	477	109960	1,95	1,18E-21	577	27697	1,72	0,000147
78	102820	4,62	4,98E-27	178	56587	3,45	1,97E-10	278	103130	2,75	3,44E-02	378	120765	2,30	1,47E-08	478	102785	1,95	3,34E-12	578	58561	1,72	1,08E-34
79	122972	4,62	1,12E-197	179	72072	3,44	1,57E-02	279	69611	2,75	1,44E-66	379	71059	2,30	2,35E-21	479	66394	1,95	2,81E-43	579	71170	1,71	3,65E-24
80	22210	4,60	9,43E-171	180	58282	3,44	1,33E-243	280	59205	2,74	1,04E-18	380	44700	2,30	4,12E-08	480	123911	1,94	1,05E-16	580	22004	1,71	1,44E-37
81	70373	4,59	2,37E-107	181	105924	3,43	4,37E-10	281	78611	2,73	1,84E-39	381	71072	2,30	3,82E-07	481	65854	1,94	1,31E-16	581	31367	1,71	3,75E-13
82	70608	4,58	1,04E-02	182	68590	3,43	7,15E-45	282	61293	2,73	9,26E-34	382	64274	2,29	2,14E-46	482	72137	1,93	9,20E-06	582	80654	1,71	5,25E-09
83	57749	4,58	3,51E-32	183	65153	3,42	1,05E-95	283	3739	2,73	1,85E-33	383	4308	2,29	7,26E-52	483	43893	1,93	6,51E-49	583	119896	1,70	2,72E-42
84	62693	4,57	2,87E-208	184	22093	3,40	4,40E-88	284	58381	2,73	5,10E-68	384	108870	2,29	3,43E-12	484	78626	1,91	9,96E-34	584	4454	1,70	0,001029
85	3914	4,55	3,41E-04	185	66657	3,39	7,86E-62	285	2489	2,73	3,17E-15	385	111514	2,29	3,95E-03	485	53475	1,91	7,31E-15	585	70319	1,70	0,000184
86	66776	4,55	1,54E-02	186	50894	3,39	1,91E-43	286	104140	2,71	3,32E-03	386	49048	2,28	1,47E-53	486	123888	1,90	3,93E-35	586	43397	1,70	4,44E-12
87	77743	4,54	2,11E-64	187	102999	3,39	1,79E-35	287	77299	2,71	9,60E-34	387	104551	2,28	5,40E-07	487	4430	1,90	9,44E-10	587	70803	1,69	3,51E-06
88	68122	4,52	4,74E-212	188	106330	3,38	1,17E-143	288	64397	2,71	2,49E-129	388	108477	2,28	1,06E-99	488	78833	1,90	2,02E-22	588	70808	1,69	3,1E-19
89	22459	4,51	1,77E-226	189	66544	3,37	1,77E-03	289	4109	2,71	6,47E-30	389	123723	2,27	4,96E-98	489	27770	1,90	5,32E-16	589	70351	1,69	1,14E-26
90	58899	4,51	4,88E-247	190	65891	3,34	9,94E-146	290	68036	2,70	3,14E-107	390	46002	2,27	1,36E-41	490	111362	1,89	1,43E-41	590	22252	1,69	6,63E-53
91	61374	4,51	3,31E-78	191	122874	3,34	1,51E-36	291	60565	2,70	3,29E-53	391	106676	2,25	1,28E-13	491	60378	1,89	1,32E-09	591	103147	1,69	3,92E-16
92	111849	4,50	5,66E-49	192	106575	3,33	1,01E-55	292	106168	2,70	4,02E-02	392	110238	2,25	8,44E-40	492	103959	1,88	4,73E-12	592	57098	1,68	1,43E-51
93	105718	4,48	1,47E-02	193	5345	3,32	2,72E-25	293	47268	2,69	2,97E-85	393	66929	2,25	2,43E-06	493	105888	1,88	3,94E-07	593	70842	1,68	4,59E-08
94	55630	4,48	4,32E-24	194	108340	3,30	1,12E-11	294	105237	2,68	1,51E-03	394	111957	2,24	5,64E-10	494	27259	1,88	8,30E-08	594	105810	1,68	3,71E-07
95	106043	4,45	5,26E-66	195	65744	3,30	1,05E-28	295	107935	2,68	7,02E-06	395	79835	2,23	5,15E-59	495	23367	1,88	4,28E-30	595	120381	1,68	1,3E-41
96	3049	4,42	4,34E-157	196	105408	3,30	2,70E-23	296	26163	2,67	4,03E-04	396	52520	2,23	6,63E-57	496	44175	1,87	4,58E-64	596	81014	1,68	2,18E-22
97	104077	4,40	1,40E-76	197	57322	3,29	2,23E-16	297	3718	2,67	2,43E-33	397	103034	2,23	8,06E-09	497	5366	1,87	1,20E-51	597	76822	1,68	4,87E-35
98	74745	4,40	5,75E-243	198	123241	3,29	1,42E-91	298	56830	2,67	3,59E-38	398	5807	2,22	1,12E-07	498	104393	1,87	3,08E-13	598	119839	1,68	2,68E-28
99	123649	4,38	2,65E-21	199	120079	3,28	5,52E-07	299	2211	2,66	5,31E-20	399	121418	2,22	6,04E-03	499	74070	1,87	2,06E-77	599	68455	1,67	8,39E-31
100	122375	4,38	5,47E-04	200	75290	3,27	1,83E-13	300	69613	2,66	1,34E-09	400	67938	2,22	4,12E-08	500	123771	1,87	7,18E-32	600	66854	1,67	3,92E-06

Supplementary Table S11 (continued): FULL list of DEGs from RNA-Seq data for Δ*ngs1* (jgi|Trire2|79669) grown on GlcNAc and compared to control (WT grown on GlcNAc).

Differentially expressed genes (DEGs) were defined by fold change ≥|1.0| and a adjusted p-value (padj) of < 0.05 found by DESeq2 (RStudio 2022.07.2+576)

	Identifier (TRIREDRAFT)	log2fc	padj		Identifier (TRIREDRAFT)	log2fc	padj		Identifier (TRIREDRAFT)	log2fc	padj		Identifier (TRIREDRAFT)	log2fc	padj		Identifier (TRIREDRAFT)	log2fc	padj
601	57860	1,67	5,57E-37	701	80026	1,47	9,13E-13	801	110878	1,30	4,81E-05	901	107502	1,17	5,63E-08	1001	3372	1,05	3,36E-19
602	5942	1,66	5,47E-12	702	104867	1,47	7,69E-03	802	65029	1,30	9,43E-14	902	65992	1,17	2,37E-14	1002	112319	1,05	2,61E-06
603	3405	1,66	4,59E-14	703	74215	1,47	1,53E-02	803	60282	1,30	1,33E-18	903	106242	1,17	4,84E-05	1003	119607	1,04	2,49E-08
604	59364	1,66	5,65E-08	704	82374	1,47	7,64E-13	804	123699	1,29	9,04E-24	904	57237	1,17	3,08E-09	1004	122717	1,04	1,76E-15
605	112520	1,65	2,31E-09	705	22694	1,47	3,53E-24	805	60897	1,29	6,65E-23	905	122995	1,16	1,25E-10	1005	61863	1,04	2,03E-20
606	56418	1,65	8,72E-06	706	107848	1,47	8,54E-15	806	78799	1,29	7,52E-37	906	120654	1,16	7,56E-20	1006	122147	1,04	6,26E-06
607	120106	1,65	4,29E-48	707	43961	1,47	1,67E-34	807	60578	1,29	1,74E-21	907	119933	1,16	2,48E-08	1007	59796	1,04	8,55E-18
608	64018	1,65	1,50E-09	708	123922	1,47	2,30E-41	808	49970	1,29	1,30E-36	908	123327	1,16	1,03E-13	1008	78496	1,04	2,47E-18
609	2231	1,65	1,94E-33	709	67983	1,47	2,15E-35	809	81457	1,28	3,48E-23	909	105251	1,16	8,76E-11	1009	120404	1,03	1,35E-04
610	122666	1,64	4,45E-43	710	62556	1,46	2,26E-17	810	59073	1,28	5,42E-16	910	53558	1,16	1,14E-06	1010	121297	1,03	2,37E-09
611	21659	1,64	1,05E-45	711	108553	1,46	3,97E-14	811	62300	1,28	1,12E-08	911	107386	1,16	3,22E-12	1011	65969	1,03	6,13E-22
612	54659	1,63	2,12E-05	712	106829	1,45	7,32E-21	812	67931	1,28	6,98E-18	912	108357	1,16	8,50E-16	1012	75424	1,03	4,35E-02
613	65718	1,63	1,05E-13	713	105979	1,45	6,49E-05	813	78738	1,28	1,05E-20	913	80200	1,16	2,88E-33	1013	42264	1,03	1,49E-06
614	2916	1,63	3,95E-20	714	62747	1,45	5,29E-13	814	65085	1,28	1,29E-02	914	80240	1,16	3,39E-14	1014	4009	1,03	5,60E-34
615	74601	1,62	2,54E-50	715	65965	1,45	9,00E-36	815	76218	1,28	2,94E-23	915	67718	1,15	3,11E-09	1015	63692	1,03	9,74E-04
616	102906	1,62	5,59E-06	716	112516	1,45	2,53E-19	816	105238	1,28	4,80E-08	916	50593	1,15	3,58E-10	1016	33482	1,03	1,71E-05
617	103498	1,62	5,73E-15	717	74771	1,45	1,31E-15	817	71532	1,27	2,78E-06	917	71380	1,15	1,41E-28	1017	2399	1,03	5,44E-05
618	60490	1,62	1,16E-13	718	104838	1,45	9,45E-24	818	45688	1,27	8,81E-24	918	119859	1,15	3,35E-07	1018	22402	1,02	1,37E-15
619	105980	1,62	1,36E-11	719	74129	1,45	2,27E-17	819	104599	1,27	1,05E-19	919	112180	1,15	1,10E-21	1019	65782	1,02	1,64E-06
620	57975	1,61	2,63E-15	720	5656	1,45	2,40E-32	820	106029	1,27	1,59E-06	920	70355	1,15	1,54E-09	1020	108920	1,02	3,61E-15
621	60232	1,61	2,26E-15	721	21960	1,44	2,76E-44	821	124341	1,27	3,95E-18	921	43129	1,15	7,25E-10	1021	110790	1,02	2,97E-13
622	44366	1,61	3,75E-09	722	60052	1,44	2,99E-12	822	123429	1,27	1,05E-19	922	23190	1,15	1,04E-13	1022	120475	1,01	4,87E-12
623	103694	1,61	1,11E-19	723	21817	1,44	5,22E-31	823	75998	1,26	5,55E-25	923	30759	1,15	9,00E-05	1023	120697	1,01	7,95E-10
624	35202	1,61	1,90E-02	724	69823	1,44	9,34E-15	824	122102	1,26	8,03E-08	924	103064	1,15	3,63E-05	1024	80685	1,01	1,39E-10
625	122140	1,61	7,93E-05	725	56117	1,44	6,79E-27	825	35186	1,26	8,77E-04	925	110342	1,14	1,28E-04	1025	61471	1,01	2,11E-14
626	47930	1,61	2,08E-36	726	82309	1,44	4,44E-06	826	68412	1,26	2,58E-25	926	103907	1,14	1,26E-08	1026	65869	1,01	7,53E-08
627	32364	1,61	6,17E-10	727	122212	1,44	8,06E-37	827	64834	1,26	4,90E-08	927	111059	1,14	5,12E-12	1027	121098	1,01	3,13E-11
628	122187	1,61	1,42E-06	728	69574	1,44	4,49E-19	828	111681	1,26	2,49E-16	928	79169	1,14	5,93E-14	1028	122091	1,01	2,09E-22
629	68615	1,61	1,54E-38	729	4146	1,44	1,92E-06	829	103812	1,26	1,46E-15	929	3653	1,14	1,23E-10	1029	122497	1,00	1,96E-04
630	51562	1,60	5,64E-52	730	103446	1,44	9,76E-17	830	27939	1,26	2,91E-20	930	81652	1,14	1,73E-25	1030	122262	1,00	2,46E-11
631	108940	1,60	4,46E-33	731	55077	1,43	1,14E-14	831	75403	1,26	2,05E-18	931	107507	1,13	2,94E-13	1031	108356	1,00	9,70E-11
632	72086	1,60	3,34E-06	732	26160	1,43	2,46E-05	832	59315	1,26	5,11E-04	932	61020	1,13	3,27E-12	1032	123820	1,00	3,49E-02
633	67446	1,60	1,16E-39	733	32243	1,42	1,26E-02	833	3481	1,25	4,06E-23	933	68358	1,13	3,28E-02	1033	78357	1,00	1,82E-06
634	47315	1,59	3,08E-16	734	67751	1,42	2,36E-15	834	102908	1,25	3,53E-15	934	123786	1,13	2,20E-17	1034	81296	1,00	3,42E-12
635	56934	1,59	3,46E-16	735	41663	1,42	7,65E-13	835	107111	1,25	4,08E-19	935	2837	1,13	4,39E-03				
636	109277	1,59	1,78E-02	736	120371	1,42	2,74E-02	836	110813	1,25	3,70E-26	936	73039	1,13	7,47E-03				
637	80920	1,59	1,20E-14	737	111237	1,42	1,39E-09	837	70316	1,24	1,78E-11	937	70548	1,12	1,04E-24				
638	30668	1,59	3,82E-05	738	110767	1,42	1,55E-07	838	67605	1,24	1,28E-04	938	111672	1,12	2,38E-02				
639	124092	1,59	2,20E-02	739	23146	1,42	5,74E-27	839	31210	1,24	5,56E-13	939	41761	1,12	7,74E-13				
640	120912	1,58	2,33E-28	740	57776	1,42	2,73E-06	840	46902	1,24	1,46E-31	940	111888	1,12	1,69E-05				
641	80756	1,58	5,65E-51	741	119534	1,41	5,02E-24	841	108637	1,24	2,99E-07	941	74807	1,12	2,81E-18				
642	122527	1,58	5,94E-14	742	5598	1,41	4,33E-36	842	121597	1,24	1,83E-20	942	121439	1,12	1,97E-04				
643	70397	1,58	6,31E-18	743	123278	1,41	1,16E-04	843	108591	1,24	4,20E-05	943	107253	1,12	1,48E-04				
644	68843	1,58	2,81E-29	744	59095	1,41	2,93E-16	844	70542	1,24	3,46E-19	944	23083	1,12	3,85E-16				
645	122153	1,58	2,14E-32	745	110414	1,41	4,71E-15	845	77512	1,24	3,98E-24	945	109929	1,12	2,10E-14				
646	110768	1,58	5,03E-14	746	67484	1,40	1,21E-29	846	66480	1,23	2,03E-35	946	70500	1,11	1,72E-02				
647	80091	1,57	3,33E-23	747	107914	1,40	1,55E-02	847	67117	1,23	7,95E-12	947	79725	1,10	7,89E-08				
648	21758	1,57	8,61E-16	748	81420	1,40	7,32E-04	848	60270	1,23	2,36E-15	948	63653	1,10	3,30E-04				
649	109677	1,57	1,10E-18	749	121475	1,40	3,71E-37	849	123354	1,23	2,55E-24	949	62165	1,10	3,59E-07				
650	121251	1,57	9,27E-24	750	105970	1,40	2,03E-16	850	69926	1,22	3,56E-02	950	121889	1,10	8,58E-19				
651	68130	1,57	7,64E-42	751	4480	1,40	1,54E-25	851	73881	1,22	1,35E-24	951	123499	1,10	2,12E-29				
652	78499	1,56	1,34E-43	752	53500	1,40	3,98E-31	852	111887	1,22	1,93E-03	952	110490	1,10	7,13E-03				
653	107704	1,56	9,06E-06	753	70859	1,39	3,80E-28	853	106154	1,22	4,22E-02	953	108191	1,10	8,98E-08				
654	123658	1,56	4,54E-34	754	41171	1,39	7,07E-09	854	55668	1,22	1,47E-10	954	108014	1,10	1,60E-04				
655	69695	1,56	2,80E-07	755	102487	1,39	1,00E-13	855	105239	1,22	4,44E-17	955	111245	1,09	6,15E-05				
656	123989	1,56	6,53E-10	756	68961	1,39	3,88E-04	856	74406	1,22	2,74E-28	956	68000	1,09	1,12E-04				
657	122974	1,56	1,43E-22	757	62805	1,38	1,13E-02	857	120189	1,22	8,05E-24	957	123460	1,09	8,76E-13				
658	105844	1,56	1,31E-08	758	121608	1,38	2,45E-14	858	119876	1,22	2,23E-15	958	60458	1,09	1,64E-02				
659	74971	1,56	6,31E-19	759	81110	1,38	1,94E-29	859	106720	1,22	3,68E-08	959	82317	1,09	7,63E-07				
660	81097	1,55	3,12E-19	760	70630	1,38	4,73E-33	860	78462	1,22	8,98E-05	960	77373	1,09	1,79E-06				
661	41518	1,54	3,59E-50	761	30250	1,37	1,01E-02	861	109275	1,22	3,17E-15	961	61532	1,09	2,78E-26				
662	80973	1,54	1,33E-23	762	124338	1,37	1,02E-05	862	70907	1,22	1,75E-06	962	55731	1,08	2,58E-22				
663	82403	1,54	9,05E-36	763	124059	1,37	1,67E-11	863	124157	1,21	3,73E-20	963	5363	1,08	8,20E-07				
664	49295	1,53	1,28E-38	764	69375	1,37	2,26E-09	864	54502	1,21	6,89E-20	964	56744	1,08	3,77E-16				
665	60422	1,53	3,74E-15	765	105863	1,37	5,44E-24	865	79823	1,21	1,41E-23	965	54366	1,08	1,12E-08				
666	107670	1,53	1,36E-32	766	53091	1,36	4,39E-02	866	44178	1,21	5,26E-21	966	53824	1,08	4,54E-02				
667	123686	1,53	3,75E-24	767	106584	1,36	4,83E-02	867	55407	1,21	2,97E-13	967	109895	1,08	1,54E-21				
668	60177	1,53	6,81E-12	768	54846	1,36	4,34E-11	868	122920	1,21	5,46E-32	968	121942	1,08	1,01E-17				
669	76324	1,53	2,19E-04	769	107012	1,36	6,59E-23	869	73179	1,21	2,18E-11	969	103158	1,08	2,31E-04				
670	65677	1,52	2,59E-17	770	53561	1,35	1,06E-08	870	25224	1,21	2,26E-09	970	121252	1,08	6,62E-27				
671	65547	1,52	2,75E-07	771	78391	1,35	5,61E-19	871	22560	1,21	4,19E-16	971	121602	1,08	3,30E-11				
672	107339	1,52	5,40E-03	772	122131	1,35	1,63E-16	872	123827	1,21	3,28E-22	972	67239	1,08	6,33E-15				
673	81511	1,52	2,82E-20	773	60517	1,35	1,14E-06	873	78797	1,21	1,41E-22	973	112613	1,07	1,06E-02				
674	65190	1,52	3,13E-04	774	108819	1,35	3,83E-04	874	77739	1,21	1,97E-02	974	77364	1,07	1,13E-10				
675	104072	1,52	1,37E-04	775	103482	1,35	1,01E-11	875	70098	1,21	7,20E-11	975	122571	1,07	1,65E-13				
676	69316	1,52	7,35E-08	776	76141	1,35	4,08E-18	876	123992	1,20	1,23E-07	976	123616	1,07	2,84E-09				
677	74057	1,51	3,08E-20	777	112551	1,35	2,27E-14	877	3464	1,20	9,88E-26	977	58587	1,07	2,41E-18				
678	28185	1,51	1,24E-03	778	110658	1,35	2,60E-32	878	62285	1,20	7,12E-13	978	25244	1,07	8,83E-15				
679	104511	1,51	1,99E-09	779	66111	1,34	1,61E-36	879	59778	1,20	1,33E-13	979	103282	1,07	8,10E-19				
680	108557	1,50	8,11E-08	780	45624	1,34	6,60E-29	880	22661	1,20	9,28E-29	980	58011	1,07	4,93E-09				
681	70201	1,50	2,14E-02	781	62171	1,34	3,53E-02	881	75175	1,20	5,75E-07	981	47710	1,07	2,24E-06				
682	105330	1,50	4,10E-05	782	58387	1,34	2,62E-33	882	58285	1,20	1,10E-02	982	58391	1,07	3,05E-15				
683	103825	1,50	1,12E-03	783	47479	1,33	1,24E-16	883	104364	1,20	1,59E-29	983	76758	1,07	3,35E-02				
684	82544	1,50	5,17E-28	784	53373	1,33	9,47E-12	884	63152	1,20	5,00E-19	984	53903	1,07	2,20E-13				
685	61278	1,49	1,23E-04	785	64172	1,33	7,56E-20	885	122284	1,20	7,08E-25	985	59338	1,07	3,72E-04				
686	107042	1,49	1,93E-02	786	104925	1,32	2,94E-15	886	32849	1,19	1,51E-11	986	77667	1,07	5,57E-21				
687	82146	1,49	5,40E-16	787	82145	1,32	3,06E-14	887	54870	1,19	1,37E-22	987	58765	1,06	1,59E-06				
688	120008	1,49	1,35E-32	788	70639	1,32	1,03E-06	888	34221	1,19	1,43E-02	988	122828	1,06	2,67E-08				
689	111135	1,49	3,38E-09	789	58772	1,32	2,77E-21	889	120943	1,19	3,56E-20	989	102723	1,06	1,34E-27				
690	70108	1,49	4,05E-03	790	121285	1,31	1,05E-16	890	112330	1,19	2,58E-09	990	123571	1,06	2,90E-19				
691	75742	1,48	6,61E-40	791	110901	1,31	3,01E-02	891	32293	1,18	1,68E-03	991	42848	1,06	2,10E-14				
692	59649	1,48	3,19E-04	792	69026	1,31	5,46E-16	892	70383	1,18	9,69E-21	992	103852	1,06	2,51E-19				
693	26255	1,48	2,63E-07	793	67538	1,31	5,78E-05	893	63202	1,18	6,49E-15	993	31227	1,06	1,92E-18				
694	120998	1,48	3,06E-63	794	60018	1,31	8,50E-06	894	102737	1,18	1,74E-19	994	106244	1,06	1,09E-10				
695	104390	1,48	5,68E-27	795	110877	1,31	1,01E-09	895	59028	1,18	9,78E-04	995	59152	1,06	2,96E-04				
696	108833	1,48	3,15E-25	796	110880	1,31	6,45E-03	896	73516	1,18	9,47E-25	996	123244	1,05	9,13E-23				
697	30578	1,48	5,54E-03	797	123675	1,31	6,61E-09	897	123120	1,18	7,50E-17	997	2392	1,05	4,83E-20				
698	121500	1,48	1,61E-22	798	122856	1,31	3,82E-37	898	23458	1,17	1,90E-11	998	69489	1,05	1,35E-18				
699	102743	1,47	1,36E-22	799	80252	1,31	9,04E-20	899	62367	1,17	2,38E-16	999	120749	1,05	2,14E-05				
700	61304	1,47	1,03E-05	800	124002	1,30	3,83E-12	900	78650	1,17	5,25E-08	1000	102776	1,05	3,25E-11				

Supplementary Table S12: FULL list of DEGs from RNA-Seq data for Δ*ron1* (jgi|Trire2|79673) grown on GlcNAc and compared to control (WT grown on GlcNAc).

Differentially expressed genes (DEGs) were defined by fold change ≥|1.0| and a adjusted p-value (padj) of < 0.05 found by DESeq2 (RStudio 2022.07.2+576)

	Identifier (TRIREDRAFT)	log2fc	padj		Identifier (TRIREDRAFT)	log2fc	padj		Identifier (TRIREDRAFT)	log2fc	padj		Identifier (TRIREDRAFT)	log2fc	padj		Identifier (TRIREDRAFT)	log2fc	padj		Identifier (TRIREDRAFT)	log2fc	padj
1	79673	-12,03	2,88E-23	101	76620	-4,18	0	201	57088	-2,96	2,58E-96	301	50212	-2,51	1,32E-119	401	121654	-2,12	9,71E-52	501	59351	-1,88	3,51E-06
2	55374	-9,00	2,07E-17	102	81819	-4,15	1,98E-03	202	55172	-2,95	3,02E-73	302	22251	-2,51	4,77E-64	402	105454	-2,12	1,03E-14	502	106895	-1,88	3,96E-16
3	79816	-8,32	2,00E-62	103	105449	-4,12	1,34E-02	203	110456	-2,94	4,67E-42	303	4561	-2,50	3,90E-30	403	75105	-2,10	1,03E-24	503	68279	-1,88	2,37E-18
4	106660	-8,03	3,44E-10	104	23240	-4,10	6,31E-72	204	69115	-2,94	1,83E-46	304	122301	-2,50	3,95E-105	404	121990	-2,10	4,39E-35	504	76169	-1,88	1,59E-47
5	111121	-7,88	6,09E-177	105	105983	-4,09	1,55E-02	205	110761	-2,94	1,78E-119	305	75165	-2,49	7,88E-30	405	123733	-2,10	6,60E-45	505	58698	-1,88	3,32E-22
6	106928	-7,46	6,28E-226	106	68588	-4,06	8,47E-211	206	74187	-2,93	2,12E-17	306	105313	-2,49	3,01E-49	406	124283	-2,10	2,50E-51	506	21388	-1,88	4,02E-29
7	112491	-7,24	5,08E-08	107	105448	-4,05	4,69E-02	207	47066	-2,93	1,23E-167	307	60773	-2,47	1,08E-56	407	21176	-2,10	4,09E-45	507	107577	-1,88	6,84E-32
8	77552	-7,21	2,93E-141	108	107974	-4,05	4,69E-02	208	104219	-2,92	1,00E-04	308	81442	-2,47	4,50E-74	408	80792	-2,10	7,22E-51	508	80268	-1,87	1,51E-36
9	30465	-7,21	2,25E-123	109	64049	-4,00	1,09E-45	209	60419	-2,92	2,76E-06	309	60067	-2,47	1,62E-58	409	108684	-2,09	8,44E-20	509	61298	-1,87	2,04E-02
10	5330	-7,18	6,69E-08	110	65817	-4,00	5,98E-03	210	71029	-2,89	4,79E-81	310	70996	-2,47	4,98E-27	410	3579	-2,09	5,23E-22	510	120877	-1,87	2,44E-51
11	105882	-6,88	1,52E-27	111	122992	-3,99	1,81E-66	211	43671	-2,89	8,08E-78	311	79813	-2,46	5,03E-82	411	110862	-2,09	4,63E-18	511	78836	-1,87	1,68E-79
12	103061	-6,60	2,29E-20	112	5000	-3,98	8,15E-06	212	79623	-2,89	1,57E-71	312	75506	-2,45	2,07E-79	412	69171	-2,08	1,11E-67	512	107866	-1,87	5,44E-04
13	121416	-6,47	1,14E-27	113	79741	-3,95	1,35E-215	213	57940	-2,88	7,93E-67	313	106223	-2,45	4,48E-110	413	107674	-2,08	3,57E-10	513	66551	-1,87	4,77E-04
14	102499	-6,45	2,02E-16	114	82032	-3,94	1,09E-102	214	105445	-2,87	1,71E-15	314	41152	-2,45	9,97E-12	414	2036	-2,08	1,01E-16	514	74214	-1,87	8,02E-26
15	124043	-6,44	3,99E-06	115	67806	-3,91	4,12E-86	215	107524	-2,86	2,74E-88	315	23382	-2,44	8,21E-04	415	122584	-2,07	4,78E-61	515	79817	-1,87	1,29E-43
16	107881	-6,43	0,00E+00	116	69956	-3,90	1,35E-58	216	56384	-2,86	6,77E-74	316	60676	-2,44	5,85E-86	416	105242	-2,07	5,75E-44	516	121801	-1,87	2,08E-44
17	69901	-6,38	6,77E-06	117	22110	-3,89	2,78E-42	217	122993	-2,85	1,34E-28	317	52976	-2,44	2,79E-70	417	74158	-2,06	3,05E-43	517	102416	-1,86	1,08E-23
18	69650	-6,25	5,65E-229	118	69834	-3,89	5,93E-226	218	123084	-2,85	9,88E-33	318	105455	-2,44	7,90E-40	418	68924	-2,06	1,15E-27	518	59991	-1,86	1,92E-29
19	6103	-6,17	1,68E-05	119	61223	-3,85	3,83E-122	219	44362	-2,84	3,70E-79	319	31075	-2,43	1,46E-56	419	22013	-2,06	4,92E-70	519	121074	-1,86	1,53E-44
20	58366	-6,16	2,50E-05	120	58574	-3,84	1,09E-09	220	54242	-2,84	1,71E-69	320	121399	-2,43	6,10E-89	420	123633	-2,06	6,85E-82	520	123946	-1,86	3,48E-66
21	103062	-6,12	2,13E-137	121	52489	-3,83	2,40E-196	221	122523	-2,83	3,60E-96	321	76082	-2,42	9,27E-68	421	109944	-2,06	5,58E-04	521	5164	-1,86	4,70E-43
22	58563	-6,11	2,30E-05	122	32798	-3,83	4,08E-108	222	105860	-2,83	7,92E-119	322	59402	-2,41	3,57E-20	422	65179	-2,06	8,85E-100	522	105287	-1,85	6,93E-03
23	111750	-6,08	0,00E+00	123	104335	-3,83	3,27E-02	223	81964	-2,82	5,00E-09	323	103012	-2,41	6,22E-30	423	75335	-2,06	1,53E-39	523	59002	-1,85	4,62E-03
24	69555	-6,05	6,29E-05	124	5119	-3,78	9,96E-185	224	58117	-2,82	2,77E-90	324	120570	-2,41	2,33E-49	424	103671	-2,06	1,48E-48	524	76766	-1,85	1,05E-45
25	104260	-6,03	3,65E-05	125	5787	-3,77	8,98E-91	225	105155	-2,82	1,12E-02	325	120362	-2,41	3,55E-69	425	64820	-2,05	2,56E-50	525	56176	-1,85	1,17E-31
26	108018	-6,02	1,09E-58	126	76359	-3,75	1,99E-84	226	78401	-2,82	1,50E-102	326	67013	-2,39	1,49E-06	426	81599	-2,05	9,80E-59	526	121294	-1,85	2,36E-51
27	111122	-6,01	1,93E-242	127	110653	-3,74	4,46E-269	227	61830	-2,81	4,35E-03	327	77557	-2,39	1,32E-09	427	74818	-2,05	1,97E-48	527	104491	-1,84	8,97E-51
28	111082	-6,01	2,20E-294	128	123979	-3,74	8,44E-248	228	73101	-2,81	7,20E-08	328	70090	-2,39	9,60E-04	428	120064	-2,05	3,70E-54	528	69141	-1,84	1,04E-37
29	82041	-5,94	1,19E-90	129	56804	-3,73	3,34E-02	229	58584	-2,80	1,57E-103	329	58456	-2,39	1,93E-05	429	105393	-2,04	2,05E-02	529	120864	-1,84	8,96E-30
30	110740	-5,94	1,61E-10	130	43701	-3,73	7,40E-299	230	58356	-2,79	6,09E-124	330	80922	-2,38	1,21E-70	430	110173	-2,04	1,11E-61	530	123437	-1,84	3,13E-44
31	123086	-5,94	0,00E+00	131	124295	-3,72	3,82E-64	231	68466	-2,78	1,85E-37	331	5612	-2,38	1,90E-44	431	66436	-2,04	1,53E-32	531	105977	-1,84	5,88E-13
32	122820	-5,85	7,93E-05	132	70962	-3,72	8,23E-03	232	65522	-2,76	1,59E-56	332	123475	-2,38	3,16E-90	432	106680	-2,04	1,16E-38	532	106315	-1,83	1,87E-24
33	109239	-5,80	2,91E-59	133	108749	-3,71	8,76E-123	233	121664	-2,76	3,46E-128	333	107667	-2,38	5,49E-04	433	76949	-2,03	3,77E-88	533	1702	-1,83	1,93E-44
34	73250	-5,77	8,21E-45	134	77547	-3,71	2,83E-16	234	45343	-2,75	4,28E-27	334	105848	-2,38	6,54E-20	434	77806	-2,03	4,03E-65	534	45912	-1,83	4,42E-66
35	121136	-5,77	1,41E-57	135	60493	-3,71	8,81E-03	235	5135	-2,75	2,35E-63	335	53053	-2,37	3,66E-25	435	69879	-2,03	1,30E-55	535	5812	-1,83	8,27E-31
36	52315	-5,74	1,45E-68	136	82633	-3,70	6,28E-25	236	106686	-2,74	3,37E-65	336	26871	-2,37	2,92E-56	436	76833	-2,03	8,71E-53	536	111451	-1,83	6,93E-10
37	70365	-5,73	2,96E-109	137	57015	-3,68	4,72E-07	237	122941	-2,74	3,41E-92	337	75247	-2,37	6,30E-03	437	104890	-2,02	9,09E-56	537	81690	-1,82	6,68E-44
38	121350	-5,70	0,00E+00	138	80639	-3,67	3,10E-170	238	3434	-2,73	9,13E-49	338	60337	-2,37	1,53E-03	438	34297	-2,02	1,65E-16	538	60086	-1,81	8,59E-18
39	122824	-5,51	1,21E-54	139	111239	-3,67	4,07E-08	239	55566	-2,73	2,22E-06	339	107073	-2,36	1,09E-23	439	44449	-2,02	5,08E-61	539	35109	-1,81	2,02E-04
40	49274	-5,43	1,24E-158	140	64167	-3,66	5,41E-134	240	78387	-2,72	3,31E-105	340	22689	-2,36	3,48E-51	440	65743	-2,02	1,58E-11	540	65191	-1,80	2,70E-41
41	69904	-5,36	5,04E-96	141	106661	-3,65	4,21E-02	241	57632	-2,70	2,85E-73	341	67008	-2,35	1,42E-11	441	77770	-2,01	5,45E-08	541	104595	-1,80	5,43E-47
42	68371	-5,36	7,91E-53	142	110762	-3,64	1,05E-30	242	41501	-2,70	1,64E-16	342	106606	-2,35	4,69E-20	442	70520	-2,01	2,01E-37	542	4244	-1,80	2,53E-16
43	22331	-5,34	7,16E-244	143	104106	-3,64	1,34E-02	243	69625	-2,70	1,40E-08	343	121107	-2,34	1,55E-129	443	103039	-2,01	9,98E-13	543	106626	-1,79	5,29E-28
44	112034	-5,26	7,04E-19	144	72922	-3,63	1,09E-114	244	59272	-2,69	1,78E-67	344	123702	-2,34	2,20E-90	444	105820	-2,00	2,33E-04	544	52248	-1,79	7,52E-31
45	50793	-5,23	1,22E-41	145	112014	-3,61	1,61E-03	245	80867	-2,69	2,07E-125	345	63687	-2,33	5,51E-43	445	4294	-2,00	3,66E-35	545	77969	-1,79	1,12E-28
46	78072	-5,19	1,51E-05	146	76800	-3,57	5,57E-273	246	68662	-2,69	5,02E-30	346	42181	-2,33	1,96E-21	446	121111	-2,00	8,47E-10	546	105775	-1,79	4,82E-25
47	122795	-5,18	1,47E-162	147	4860	-3,56	3,01E-94	247	58731	-2,69	4,59E-03	347	110711	-2,32	2,51E-08	447	78062	-1,99	3,77E-42	547	62225	-1,79	5,82E-35
48	69696	-5,09	1,54E-09	148	4517	-3,56	3,87E-11	248	82049	-2,68	3,78E-78	348	123539	-2,32	1,60E-16	448	54129	-1,99	2,50E-03	548	109206	-1,79	1,95E-09
49	81576	-5,04	0,00E+00	149	123468	-3,55	1,04E-16	249	39587	-2,68	8,64E-04	349	48301	-2,32	1,29E-47	449	105171	-1,99	1,24E-02	549	78732	-1,79	4,10E-66
50	3094	-5,02	1,39E-87	150	80056	-3,54	2,81E-12	250	59746	-2,68	5,50E-08	350	2716	-2,31	4,47E-15	450	69537	-1,99	2,55E-03	550	107037	-1,79	1,09E-10
51	122324	-4,97	7,31E-04	151	121139	-3,53	9,99E-123	251	124277	-2,68	3,02E-05	351	122084	-2,30	8,67E-27	451	80863	-1,98	7,03E-43	551	75304	-1,79	2,27E-57
52	76763	-4,95	1,19E-33	152	2852	-3,52	5,13E-31	252	67026	-2,67	4,32E-72	352	108409	-2,30	4,77E-49	452	5710	-1,98	2,84E-49	552	112001	-1,79	8,72E-36
53	105444	-4,91	7,91E-210	153	47424	-3,51	6,84E-53	253	64869	-2,67	1,82E-06	353	80872	-2,30	5,19E-80	453	124296	-1,98	2,27E-06	553	43115	-1,78	1,76E-14
54	59655	-4,91	1,50E-06	154	70949	-3,51	1,01E-19	254	121686	-2,67	8,17E-151	354	105729	-2,29	4,58E-02	454	70527	-1,98	1,85E-04	554	3823	-1,78	1,66E-48
55	23353	-4,89	1,03E-193	155	109372	-3,51	2,39E-28	255	109945	-2,67	5,48E-60	355	81586	-2,29	1,77E-67	455	3641	-1,98	5,14E-76	555	121868	-1,78	3,01E-37
56	76238	-4,85	1,33E-300	156	107641	-3,50	1,58E-02	256	75704	-2,67	2,25E-87	356	122031	-2,27	1,11E-17	456	22386	-1,97	2,16E-06	556	21453	-1,78	2,53E-28
57	112643	-4,84	4,18E-24	157	68585	-3,49	2,50E-11	257	66935	-2,66	5,92E-44	357	54064	-2,27	1,94E-52	457	110765	-1,97	1,78E-03	557	68803	-1,78	1,87E-16
58	73623	-4,83	2,67E-06	158	58853	-3,45	6,45E-144	258	30776	-2,65	9,36E-03	358	51130	-2,27	1,03E-73	458	74943	-1,97	6,70E-44	558	56196	-1,78	3,43E-20
59	65410	-4,82	0,00E+00	159	76374	-3,44	5,68E-61	259	105533	-2,65	4,42E-32	359	105465	-2,26	3,54E-11	459	22264	-1,97	6,81E-38	559	77647	-1,78	4,58E-13
60	110127	-4,82	0,00E+00	160	120359	-3,44	1,88E-79	260	45138	-2,64	2,13E-106	360	72408	-2,26	6,81E-23	460	121372	-1,96	4,32E-49	560	64312	-1,78	1,34E-13
61	80086	-4,82	5,96E-114	161	56771	-3,44	3,25E-39	261	109101	-2,64	1,33E-12	361	108683	-2,25	1,04E-05	461	122076	-1,96	1,72E-55	561	63774	-1,77	7,97E-25
62	121534	-4,80	4,94E-270	162	27706	-3,43	3,27E-09	262	59065	-2,64	4,56E-11	362	120879	-2,25	3,85E-22	462	78956	-1,96	1,26E-36	562	120147	-1,77	7,3018E-40
63	80879	-4,79	2,85E-184	163	106161	-3,42	3,38E-46	263	110316	-2,64	5,84E-49	363	109850	-2,24	1,70E-85	463	68988	-1,96	4,65E-02	563	109846	-1,77	5,8853E-05
64	122825	-4,74	3,55E-104	164	69692	-3,42	2,62E-20	264	122629	-2,63	2,58E-40	364	3671	-2,23	4,76E-86	464	66154	-1,95	5,72E-42	564	123999	-1,77	3,3333E-29
65	104067	-4,73	2,83E-06	165	65039	-3,41	4,72E-67	265	66517	-2,63	1,13E-61	365	124333	-2,22	3,30E-22	465	108299	-1,95	6,53E-57	565	74356	-1,76	7,4416E-49
66	103193	-4,72	8,79E-03	166	102500	-3,41	9,83E-17	266	46128	-2,63	6,84E-22	366	122572	-2,21	1,61E-81	466	103822	-1,95	5,15E-20	566	110363	-1,76	0,00266688
67	124084	-4,67	1,61E-57	167	23292	-3,40	1,21E-53	267	103179	-2,63	2,68E-07	367	122233	-2,21	2,18E-64	467	107835	-1,95	6,73E-05	567	42053	-1,75	7,9148E-23
68	59876	-4,62	7,19E-66	168	67109	-3,39	1,23E-26	268	69742	-2,63	1,90E-07	368	56291	-2,21	7,68E-21	468	120568	-1,94	5,48E-100	568	22914	-1,75	4,0128E-64
69	66345	-4,60	0,00E+00	169	60182	-3,38	6,99E-11	269	46285	-2,62	1,99E-19	369	65315	-2,21	4,90E-31	469	80820	-1,94	4,42E-66	569	107297	-1,75	3,3893E-10
70	68831	-4,60	2,86E-43	170	61526	-3,36	4,82E-48	270	106627	-2,62	2,80E-52	370	124282	-2,20	7,09E-105	470	5742	-1,94	9,82E-32	570	123251	-1,75	3,4826E-19
71	105805	-4,59	1,24E-02	171	111966	-3,35	2,16E-05	271	108583	-2,62	6,49E-78	371	66041	-2,20	9,66E-51	471	59597	-1,94	1,48E-03	571	49112	-1,75	2,1617E-44
72	111838	-4,59	7,20E-142	172	109373	-3,34	1,53E-43	272	72780	-2,61	2,47E-70	372	65883	-2,20	1,13E-06	472	68812	-1,94	8,07E-37	572	46244	-1,75	1,7641E-35
73	105291	-4,58	1,41E-82	173	120923	-3,34	2,97E-06	273	121337	-2,61	6,93E-03	373	109394	-2,20	2,14E-10	473	31798	-1,93	1,09E-24	573	122296	-1,74	2,1147E-36
74	63125	-4,54	3,15E-27	174	111865	-3,32	6,45E-05	274	104081	-2,60	3,92E-15	374	79244	-2,20	1,14E-85	474	52222	-1,93	7,13E-31	574	62769	-1,74	2,1543E-28
75	110457	-4,54	1,35E-137	175	58472	-3,32	1,95E-08	275	56911	-2,60	2,12E-13	375	78679	-2,20	2,32E-99	475	107335	-1,93	6,48E-36	575	5233	-1,74	3,6929E-59
76	103049	-4,48	1,49E-32	176	105154	-3,31	2,79E-02	276	54089	-2,60	7,05E-04	376	121706	-2,20	1,65E-58	476	123009	-1,92	5,50E-25	576	74913	-1,74	6,0452E-20
77	54461	-4,48	2,01E-79	177	59333	-3,29	4,62E-04	277	70491	-2,60	1,16E-04	377	65975	-2,19	3,15E-39	477	81742	-1,92	4,47E-32	577	80748	-1,74	1,2668E-29
78	69181	-4,48	1,52E-02	178	105931	-3,29	2,89E-04	278	82095	-2,59	5,63E-66	378	121498	-2,18	9,72E-06	478	58227	-1,92	7,46E-06	578	122531	-1,74	4,026E-42
79	3488	-4,47	5,68E-66	179	79738	-3,24	4,12E-84	279	111881	-2,59	1,33E-02	379	120260	-2,18	1,59E-50	479	4152	-1,92	4,38E-65	579	55766	-1,73	2,0409E-40
80	76601	-4,44	3,93E-22	180	81096	-3,23	1,11E-19	280	104181	-2,58	1,03E-02	380	111345	-2,18	3,71E-07	480	75116	-1,92	2,51E-51	580	34112	-1,73	3,5821E-50
81	120953	-4,42	1,28E-124	181	112493	-3,20	6,83E-04	281	39637	-2,57	3,34E-55	381	111739	-2,17	2,00E-37	481	74442	-1,92	4,80E-32	581	103112	-1,73	6,0005E-12
82	66766	-4,41	1,52E-10	182	76215	-3,18	4,79E-129	282	58774	-2,57	3,82E-07	382	59624	-2,17	1,90E-23	482	76722	-1,91	2,31E-41	582	108332	-1,73	1,1155E-31
83	3327	-4,41	1,75E-25	183	111912	-3,17	3,41E-71	283	109371	-2,56	7,00E-18	383	108686	-2,16	2,11E-23	483	58753	-1,91	3,72E-29	583	63657	-1,73	2,2025E-35
84	108784	-4,39	5,80E-32	184	77481	-3,15	9,68E-132	284	64617	-2,56	1,41E-35	384	69652	-2,16	8,20E-21	484	6108	-1,91	3,98E-21	584	22129	-1,73	1,0464E-27
85	111837	-4,37	3,64E-170	185	105379	-3,14	3,42E-03	285	80607	-2,56	4,37E-12	385	109243	-2,16	1,63E-04	485	104200	-1,91	1,33E-30	585	74480	-1,73	2,9551E-37
86	67778	-4,37	7,02E-03	186	56350	-3,12	4,32E-07	286	67509	-2,56	4,30E-11	386	68997	-2,16	4,11E-06	486	110409	-1,91	1,32E-44	586	124056	-1,73	0,002155
87	81082	-4,36	6,40E-149	187	122074	-3,12	2,98E-105	287	74156	-2,56	6,04E-37	387	111251	-2,16	8,40E-44	487	112556	-1,91	2,57E-17	587	107850	-1,73	7,6151E-13
88	69863	-4,34	6,33E-200	188	61350	-3,12	8,15E-60	288	49898	-2,55	3,06E-29	388	112288	-2,16	1,07E-04	488	109443	-1,91	2,74E-43	588	70934	-1,73	0,00139519
89	109321	-4,34	2,28E-02	189	58333	-3,11	1,39E-74	289	108160	-2,55	4,16E-08	389	50215	-2,15	3,26E-59	489	77795	-1,91	4,23E-72	589	106452	-1,72	2,3743E-19
90	122506	-4,32	1,65E-173	190	69605	-3,11	1,08E-03	290	123718	-2,55	2,84E-42	390	124119	-2,14	1,23E-71	490	65948	-1,90	1,29E-11	590	121214	-1,72	1,2841E-41
91	62716	-4,31	4,68E-133	191	68813	-3,10	8,76E-77	291	107229	-2,55	2,53E-02	391	82296	-2,14	3,75E-92	491	104222	-1,90	1,42E-25	591	61605	-1,71	0,02148644
92	105840	-4,31	1,04E-198	192	105869	-3,08	3,56E-05	292	122792	-2,54	1,21E-62	392	76971	-2,14	4,95E-57	492	58767	-1,90	3,04E-03	592	63107	-1,71	1,7268E-05
93	110879	-4,31	3,18E-09	193	60847	-3,05	3,72E-125	293	102607	-2,54	1,98E-80	393	4654	-2,14	1,33E-81	493	73523	-1,90	8,83E-80	593	119788	-1,71	3,2787E-42
94	105156	-4,29	1,30E-12	194	123149	-3,05	5,04E-72	294	6005	-2,53	8,28E-53	394	103032	-2,14	3,83E-02	494	79037	-1,90	4,92E-58	594	111023	-1,71	8,6343E-29
95	124198	-4,27	3,47E-11	195	65615	-3,04	6,31E-29	295	38812	-2,53	3,33E-67	395	66963	-2,13	7,07E-36	495	104050	-1,90	1,46E-02	595	60758	-1,71	4,8827E-13
96	105224	-4,26	4,68E-113	196	43101	-3,02	4,77E-11	296	60144	-2,53	1,55E-03	396	110382	-2,13	6,45E-03	496	123648	-1,90	1,79E-17	596	49979	-1,71	1,306E-40
97	108781	-4,23	8,09E-143	197	73536	-3,02	4,74E-129	297	110830	-2,53	8,71E-07	397	53238	-2,13	1,31E-52	497	73631	-1,89	7,93E-18	597	107187	-1,70	7,6963E-50
98	110317	-4,22	3,10E-02	198	109045	-3,00	6,76E-03	298	53673	-2,52	5,88E-110	398	56434	-2,13	5,51E-59	498	123220	-1,89	7,18E-42	598	67325	-1,70	4,0346E-22
99	107003	-4,22	3,28E-02	199	65583	-2,97	1,14E-12	299	78264	-2,52	1,94E-70	399	124149	-2,13	9,69E-57	499	103222	-1,89	9,10E-63	599	122778	-1,70	1,6539E-25
100	59362	-4,21	1,66E-12	200	62463	-2,97	3,67E-64	300	112665	-2,51	3,19E-10	400	70830	-2,13	2,51E-25	500	67907	-1,88	3,24E-36	600	106437	-1,70	2,3469E-13

Supplementary Table S12 (continued): FULL list of DEGs from RNA-Seq data for Δ*ron1* (jgi|Trire2|79673) grown on GlcNAc and compared to control (WT grown on GlcNAc).

Differentially expressed genes (DEGs) were defined by fold change ≥|1.0| and a adjusted p-value (padj) of < 0.05 found by DESeq2 (RStudio 2022.07.2+576)

	Identifier (TRIREDRAFT)	log2fc	padj		Identifier (TRIREDRAFT)	log2fc	padj		Identifier (TRIREDRAFT)	log2fc	padj		Identifier (TRIREDRAFT)	log2fc	padj		Identifier (TRIREDRAFT)	log2fc	padj		Identifier (TRIREDRAFT)	log2fc	padj
601	79545	-1,70	2,27E-52	701	21658	-1,56	1,45E-68	801	59770	-1,45	1,53E-03	901	61550	-1,34	5,61E-22	1001	63157	-1,24	6,85E-18	1101	106798	-1,13	1,60E-16
602	121133	-1,69	2,21E-50	702	104359	-1,56	1,43E-07	802	3501	-1,45	2,79E-22	902	112146	-1,34	9,37E-09	1002	124010	-1,24	3,84E-40	1102	54448	-1,13	1,34E-17
603	109081	-1,69	2,60E-06	703	54865	-1,56	2,03E-10	803	120540	-1,45	3,98E-31	903	121757	-1,34	5,94E-22	1003	21663	-1,24	2,12E-15	1103	67430	-1,13	3,27E-09
604	112131	-1,69	5,74E-21	704	68606	-1,56	7,07E-32	804	109833	-1,45	1,10E-15	904	79059	-1,34	5,67E-43	1004	123396	-1,24	1,64E-02	1104	123382	-1,13	4,53E-29
605	105011	-1,69	3,29E-33	705	43814	-1,56	6,17E-35	805	22667	-1,45	1,40E-13	905	4442	-1,34	2,59E-04	1005	120607	-1,23	2,37E-27	1105	22143	-1,13	2,49E-18
606	123976	-1,69	2,72E-13	706	111663	-1,56	2,70E-26	806	120020	-1,45	8,76E-37	906	107857	-1,34	2,74E-21	1006	80515	-1,23	7,73E-20	1106	82547	-1,13	1,31E-27
607	42766	-1,68	7,52E-09	707	4952	-1,56	1,89E-04	807	75414	-1,44	2,05E-35	907	122287	-1,33	5,59E-32	1007	23090	-1,23	2,42E-13	1107	106160	-1,13	1,15E-08
608	75271	-1,68	1,09E-32	708	53972	-1,55	2,58E-27	808	122422	-1,44	3,10E-24	908	81389	-1,33	7,10E-16	1008	73450	-1,23	4,20E-42	1108	124256	-1,13	1,12E-21
609	4117	-1,68	4,68E-28	709	3719	-1,55	3,56E-02	809	56835	-1,44	3,03E-04	909	106821	-1,33	3,57E-02	1009	77288	-1,23	8,18E-05	1109	102593	-1,13	2,58E-16
610	78162	-1,68	3,47E-34	710	81803	-1,55	2,34E-19	810	75687	-1,44	1,12E-30	910	122794	-1,33	2,03E-31	1010	58815	-1,23	1,99E-37	1110	45852	-1,13	1,09E-10
611	42267	-1,68	1,14E-03	711	62100	-1,55	1,04E-18	811	61595	-1,44	6,75E-57	911	105647	-1,33	5,70E-18	1011	110140	-1,23	4,65E-03	1111	123174	-1,13	2,00E-25
612	61279	-1,67	2,26E-26	712	122448	-1,55	1,46E-18	812	123455	-1,44	7,52E-04	912	120267	-1,33	4,12E-30	1012	61153	-1,23	1,25E-14	1112	122176	-1,13	5,04E-21
613	76247	-1,67	1,44E-38	713	52267	-1,54	3,42E-33	813	121003	-1,44	7,23E-53	913	121906	-1,33	1,16E-53	1013	55790	-1,23	1,31E-18	1113	120610	-1,13	1,87E-15
614	122079	-1,67	1,34E-18	714	119864	-1,54	1,14E-31	814	3591	-1,43	1,60E-17	914	109338	-1,33	2,72E-06	1014	111787	-1,22	3,89E-17	1114	121579	-1,13	3,61E-03
615	123631	-1,67	1,77E-54	715	82246	-1,54	4,38E-17	815	66432	-1,43	2,28E-13	915	80025	-1,33	8,52E-53	1015	110171	-1,22	4,96E-24	1115	59078	-1,12	9,23E-05
616	102676	-1,67	7,44E-24	716	73985	-1,54	4,66E-43	816	121837	-1,43	1,64E-51	916	5536	-1,32	1,60E-19	1016	58867	-1,22	7,43E-05	1116	110708	-1,12	3,53E-03
617	121904	-1,67	3,35E-69	717	70311	-1,54	1,93E-32	817	123779	-1,43	8,71E-05	917	103132	-1,32	2,05E-25	1017	4240	-1,22	9,29E-04	1117	49205	-1,12	1,98E-17
618	109341	-1,67	6,74E-12	718	1927	-1,53	1,94E-18	818	60879	-1,43	2,15E-16	918	26029	-1,32	2,98E-21	1018	107918	-1,22	2,30E-10	1118	42249	-1,12	3,17E-22
619	51212	-1,66	9,44E-60	719	47795	-1,53	3,88E-57	819	105518	-1,43	3,65E-30	919	62059	-1,32	1,55E-19	1019	112596	-1,21	1,53E-15	1119	70829	-1,12	4,50E-04
620	107202	-1,66	3,37E-07	720	121278	-1,53	2,05E-39	820	120872	-1,43	3,72E-32	920	67493	-1,32	4,44E-18	1020	80484	-1,21	2,70E-35	1120	123614	-1,12	6,73E-23
621	123202	-1,66	9,96E-56	721	78870	-1,53	1,62E-39	821	23327	-1,42	4,05E-37	921	120571	-1,32	1,34E-18	1021	2912	-1,21	9,09E-22	1121	112083	-1,12	1,17E-02
622	51722	-1,66	6,49E-43	722	43906	-1,53	3,47E-56	822	73594	-1,42	7,65E-28	922	73560	-1,32	9,13E-17	1022	64959	-1,21	7,42E-22	1122	81362	-1,12	5,07E-10
623	23059	-1,66	6,01E-64	723	43662	-1,53	6,77E-33	823	75564	-1,42	1,36E-43	923	120752	-1,32	1,58E-23	1023	54198	-1,21	5,77E-22	1123	4064	-1,11	8,92E-20
624	57494	-1,66	5,60E-34	724	66865	-1,53	4,94E-02	824	21407	-1,42	2,31E-38	924	66616	-1,32	2,87E-03	1024	55252	-1,21	5,16E-19	1124	103599	-1,11	3,65E-19
625	123176	-1,66	1,43E-73	725	46490	-1,53	1,56E-54	825	102904	-1,42	2,64E-03	925	104557	-1,32	2,91E-04	1025	44628	-1,20	3,30E-08	1125	60328	-1,11	1,08E-09
626	74374	-1,66	2,25E-33	726	58607	-1,53	1,66E-38	826	72259	-1,42	1,37E-06	926	121834	-1,32	2,41E-26	1026	111893	-1,20	1,47E-08	1126	77495	-1,11	1,42E-27
627	51103	-1,66	2,68E-53	727	42919	-1,53	1,25E-40	827	78233	-1,42	1,05E-53	927	5855	-1,32	3,06E-02	1027	82017	-1,20	6,11E-15	1127	74252	-1,11	6,22E-16
628	5064	-1,66	9,28E-25	728	71363	-1,53	4,03E-35	828	109793	-1,42	2,95E-03	928	61750	-1,32	4,88E-28	1028	60338	-1,20	8,48E-17	1128	58427	-1,11	1,48E-21
629	106081	-1,66	2,02E-48	729	79106	-1,53	2,42E-34	829	122036	-1,42	6,42E-56	929	119845	-1,32	4,63E-12	1029	121820	-1,20	3,19E-20	1129	58396	-1,11	9,97E-03
630	36391	-1,66	2,68E-15	730	53395	-1,53	1,99E-16	830	2185	-1,42	1,78E-11	930	71970	-1,32	1,91E-15	1030	103506	-1,20	1,93E-14	1130	62643	-1,11	2,32E-05
631	103189	-1,66	2,67E-06	731	122278	-1,53	1,33E-05	831	32261	-1,41	6,52E-16	931	4981	-1,32	8,12E-19	1031	71783	-1,20	2,59E-23	1131	74987	-1,11	3,86E-10
632	39221	-1,66	6,11E-19	732	120781	-1,53	1,39E-56	832	69035	-1,41	1,08E-17	932	60591	-1,32	2,51E-12	1032	61576	-1,20	3,65E-26	1132	62335	-1,11	1,35E-21
633	105968	-1,66	3,62E-08	733	111053	-1,53	1,25E-23	833	65672	-1,41	1,14E-31	933	78409	-1,31	1,88E-36	1033	78576	-1,20	4,53E-18	1133	107743	-1,11	2,48E-02
634	6015	-1,65	4,19E-27	734	122879	-1,53	8,02E-39	834	55183	-1,41	1,32E-14	934	106116	-1,31	1,38E-02	1034	102744	-1,20	3,49E-22	1134	75514	-1,10	2,70E-19
635	110850	-1,65	1,03E-05	735	21890	-1,52	1,26E-48	835	111564	-1,41	1,97E-23	935	54616	-1,31	1,67E-15	1035	2348	-1,20	7,84E-10	1135	78320	-1,10	3,02E-15
636	109828	-1,65	1,55E-17	736	55660	-1,52	4,06E-20	836	109790	-1,40	4,65E-14	936	82667	-1,31	9,26E-19	1036	53133	-1,20	5,95E-14	1136	79287	-1,10	1,39E-17
637	58299	-1,65	3,83E-23	737	111818	-1,52	7,63E-03	837	65819	-1,40	9,02E-07	937	104353	-1,31	1,09E-07	1037	48281	-1,19	1,10E-23	1137	62213	-1,10	1,44E-24
638	103149	-1,65	1,77E-16	738	78340	-1,52	2,57E-13	838	107197	-1,40	1,42E-14	938	122141	-1,31	1,51E-51	1038	5227	-1,19	6,42E-26	1138	39942	-1,10	1,81E-23
639	75609	-1,65	1,37E-77	739	21170	-1,52	8,12E-50	839	22611	-1,40	2,92E-32	939	23208	-1,30	2,02E-08	1039	121336	-1,19	2,40E-11	1139	120219	-1,10	1,32E-21
640	62462	-1,65	1,76E-32	740	21396	-1,52	2,35E-46	840	122724	-1,40	4,71E-13	940	122998	-1,30	1,76E-08	1040	2591	-1,19	1,04E-10	1140	51430	-1,10	2,15E-33
641	105866	-1,65	2,19E-31	741	122127	-1,51	2,37E-19	841	43242	-1,40	8,49E-08	941	61965	-1,30	1,51E-33	1041	46958	-1,19	4,72E-39	1141	103138	-1,10	1,08E-14
642	107058	-1,65	2,04E-31	742	82623	-1,51	3,89E-18	842	82280	-1,40	9,62E-29	942	22739	-1,30	4,67E-12	1042	60346	-1,19	3,39E-14	1142	78953	-1,09	1,06E-19
643	109548	-1,65	5,07E-04	743	105785	-1,51	4,44E-02	843	111192	-1,40	4,24E-12	943	60752	-1,30	3,22E-02	1043	58790	-1,19	7,12E-28	1143	65711	-1,09	1,36E-21
644	104104	-1,64	1,42E-06	744	109378	-1,51	3,12E-02	844	119619	-1,40	1,20E-08	944	122659	-1,30	8,54E-29	1044	58971	-1,19	1,16E-10	1144	60255	-1,09	1,76E-23
645	122350	-1,64	4,55E-29	745	121126	-1,51	4,73E-37	845	45503	-1,40	7,19E-20	945	76677	-1,30	9,54E-22	1045	123476	-1,19	5,86E-14	1145	73837	-1,09	7,92E-28
646	120646	-1,64	7,50E-65	746	45736	-1,51	5,87E-60	846	123145	-1,40	1,90E-55	946	81089	-1,30	2,43E-15	1046	27948	-1,19	3,96E-14	1146	74774	-1,09	4,50E-28
647	23332	-1,64	6,71E-33	747	122587	-1,51	2,84E-41	847	78377	-1,40	1,53E-24	947	102497	-1,30	4,93E-31	1047	38833	-1,19	4,77E-11	1147	74486	-1,09	5,81E-20
648	80578	-1,64	4,27E-08	748	50159	-1,51	2,11E-12	848	111875	-1,40	3,99E-03	948	105191	-1,29	1,13E-14	1048	124202	-1,19	1,16E-19	1148	53669	-1,09	3,09E-21
649	56206	-1,64	4,39E-31	749	123753	-1,51	4,06E-40	849	74177	-1,40	8,91E-31	949	77906	-1,29	2,12E-35	1049	121051	-1,19	7,54E-38	1149	74346	-1,09	2,02E-22
650	122124	-1,64	1,06E-16	750	63869	-1,51	2,81E-14	850	120779	-1,39	2,87E-37	950	82516	-1,29	3,81E-23	1050	120081	-1,19	7,99E-24	1150	111074	-1,09	1,53E-21
651	51426	-1,64	1,83E-47	751	122040	-1,50	7,16E-42	851	73937	-1,39	2,28E-32	951	76740	-1,29	4,13E-23	1051	74390	-1,18	5,76E-21	1151	2250	-1,09	7,85E-08
652	60780	-1,64	1,52E-25	752	119530	-1,50	9,69E-13	852	108783	-1,39	2,40E-11	952	55240	-1,29	6,81E-04	1052	38603	-1,18	3,45E-12	1152	105613	-1,08	2,59E-14
653	76633	-1,63	1,69E-54	753	120195	-1,50	3,78E-40	853	3333	-1,39	1,03E-05	953	122505	-1,29	1,72E-13	1053	103143	-1,18	2,09E-02	1153	47136	-1,08	3,45E-24
654	124110	-1,63	1,59E-79	754	62377	-1,50	5,12E-31	854	121741	-1,39	3,78E-55	954	80746	-1,29	3,81E-04	1054	119855	-1,18	3,44E-24	1154	106641	-1,08	5,56E-07
655	110444	-1,63	5,44E-15	755	76995	-1,50	3,84E-02	855	58428	-1,39	3,69E-06	955	62820	-1,29	1,36E-21	1055	75923	-1,18	2,29E-25	1155	120430	-1,08	9,64E-16
656	104251	-1,63	5,31E-07	756	53701	-1,50	9,52E-25	856	1751	-1,39	5,83E-36	956	119555	-1,29	6,39E-27	1056	59197	-1,18	4,76E-07	1156	122416	-1,08	6,97E-09
657	106039	-1,63	3,58E-48	757	122363	-1,50	3,40E-08	857	53529	-1,39	7,45E-17	957	57291	-1,28	6,24E-14	1057	1925	-1,18	3,80E-26	1157	82613	-1,08	6,90E-23
658	121232	-1,62	7,33E-41	758	108676	-1,49	2,08E-07	858	46209	-1,38	3,20E-14	958	79495	-1,28	2,10E-34	1058	2087	-1,18	4,30E-19	1158	103442	-1,08	3,19E-14
659	54667	-1,62	7,07E-37	759	73765	-1,49	6,41E-60	859	108005	-1,38	9,48E-30	959	81713	-1,28	1,34E-44	1059	73873	-1,18	7,58E-25	1159	54596	-1,08	1,10E-09
660	68086	-1,62	3,34E-26	760	3394	-1,49	3,26E-15	860	120363	-1,38	1,56E-07	960	43418	-1,28	5,52E-24	1060	65997	-1,18	4,61E-02	1160	78688	-1,08	3,90E-21
661	122811	-1,62	7,04E-63	761	119568	-1,49	8,55E-35	861	58627	-1,38	2,99E-23	961	70197	-1,28	1,57E-04	1061	2186	-1,18	1,67E-24	1161	109031	-1,08	1,26E-06
662	104535	-1,62	1,49E-29	762	59472	-1,49	5,23E-07	862	120892	-1,38	5,75E-27	962	121605	-1,28	4,06E-27	1062	106259	-1,17	2,03E-02	1162	121178	-1,08	2,0843E-10
663	60300	-1,62	6,43E-33	763	122001	-1,49	3,34E-62	863	108132	-1,38	2,94E-12	963	71791	-1,28	1,86E-19	1063	65406	-1,17	5,74E-19	1163	104807	-1,08	2,7354E-15
664	23184	-1,62	1,12E-42	764	76360	-1,48	7,74E-03	864	59248	-1,38	1,74E-21	964	4040	-1,28	5,90E-09	1064	21398	-1,17	5,82E-12	1164	64375	-1,08	5,7999E-15
665	106491	-1,62	8,55E-04	765	2583	-1,48	5,68E-19	865	109221	-1,38	2,18E-02	965	123673	-1,28	1,39E-08	1065	104968	-1,17	1,37E-15	1165	119603	-1,08	1,4968E-24
666	119626	-1,62	4,60E-54	766	106480	-1,48	1,57E-06	866	59723	-1,38	1,11E-07	966	56467	-1,28	6,42E-03	1066	112640	-1,17	1,46E-03	1166	103162	-1,08	8,353E-07
667	49038	-1,62	2,92E-22	767	73621	-1,48	2,91E-08	867	81541	-1,37	1,39E-16	967	66311	-1,28	8,37E-05	1067	77284	-1,17	6,23E-21	1167	108827	-1,08	4,3478E-14
668	102920	-1,62	1,64E-14	768	121878	-1,48	5,57E-55	868	74568	-1,37	3,03E-55	968	122457	-1,27	8,09E-33	1068	55545	-1,17	1,11E-25	1168	61804	-1,08	2,2169E-17
669	78970	-1,62	3,80E-33	769	23323	-1,48	4,36E-26	869	112018	-1,37	3,54E-20	969	44434	-1,27	2,93E-42	1069	75921	-1,17	7,59E-14	1169	73519	-1,07	1,1421E-09
670	76532	-1,62	5,59E-79	770	63378	-1,47	1,96E-43	870	29333	-1,37	3,02E-17	970	119609	-1,27	1,29E-24	1070	66161	-1,17	2,01E-16	1170	75000	-1,07	1,9945E-08
671	44389	-1,61	2,35E-42	771	120868	-1,47	2,61E-59	871	80115	-1,37	6,88E-30	971	57558	-1,27	2,95E-04	1071	69663	-1,17	1,17E-22	1171	66562	-1,07	7,5188E-19
672	109187	-1,61	2,17E-25	772	109015	-1,47	9,08E-31	872	79568	-1,37	2,98E-39	972	110655	-1,27	1,31E-13	1072	54427	-1,16	2,68E-06	1172	22245	-1,07	4,4248E-23
673	107639	-1,61	3,45E-20	773	64065	-1,47	1,43E-21	873	123659	-1,37	2,21E-06	973	80890	-1,27	1,03E-14	1073	3568	-1,16	2,23E-03	1173	105832	-1,07	1,1733E-19
674	4213	-1,60	1,43E-16	774	65225	-1,47	7,20E-20	874	51499	-1,37	6,47E-34	974	62244	-1,27	2,74E-11	1074	2499	-1,16	1,81E-06	1174	3891	-1,07	2,776E-09
675	102855	-1,60	6,74E-12	775	121766	-1,47	3,63E-34	875	123204	-1,37	1,15E-24	975	105143	-1,26	1,17E-02	1075	2648	-1,16	8,68E-19	1175	106287	-1,07	1,8239E-13
676	48170	-1,60	7,40E-69	776	121818	-1,47	8,35E-49	876	73840	-1,36	7,78E-46	976	49770	-1,26	2,28E-14	1076	66687	-1,16	6,69E-08	1176	2845	-1,07	1,0267E-17
677	68909	-1,60	3,07E-43	777	104419	-1,47	3,18E-16	877	53153	-1,36	2,49E-09	977	110893	-1,26	3,77E-05	1077	109905	-1,16	2,64E-14	1177	1947	-1,07	8,1696E-13
678	69838	-1,59	1,97E-25	778	108953	-1,47	3,83E-17	878	106314	-1,36	8,22E-17	978	72643	-1,26	4,08E-15	1078	123471	-1,16	1,80E-24	1178	121087	-1,06	1,0564E-19
679	77656	-1,59	4,14E-62	779	112281	-1,47	7,04E-04	879	120621	-1,36	3,27E-36	979	52924	-1,26	3,30E-14	1079	67882	-1,16	1,89E-11	1179	21595	-1,06	2,1122E-05
680	120415	-1,59	1,63E-38	780	5970	-1,47	2,25E-10	880	72941	-1,36	2,37E-27	980	28050	-1,26	6,33E-25	1080	43615	-1,16	5,86E-26	1180	112328	-1,06	3,6563E-09
681	57666	-1,59	8,57E-36	781	106781	-1,47	4,31E-35	881	55029	-1,36	4,08E-15	981	68444	-1,26	4,14E-15	1081	112384	-1,16	8,00E-17	1181	68582	-1,06	9,3403E-23
682	107601	-1,59	1,22E-21	782	62153	-1,47	2,42E-28	882	70991	-1,35	9,83E-06	982	77119	-1,26	1,04E-21	1082	122882	-1,15	6,33E-24	1182	112525	-1,06	2,4501E-06
683	70204	-1,59	1,09E-02	783	54768	-1,47	5,11E-29	883	36373	-1,35	8,92E-17	983	81906	-1,26	3,29E-23	1083	21435	-1,15	2,27E-19	1183	73842	-1,06	6,1561E-21
684	46607	-1,59	1,62E-39	784	46545	-1,46	9,03E-32	884	71039	-1,35	1,93E-19	984	59515	-1,26	1,28E-17	1084	121405	-1,15	3,72E-19	1184	122868	-1,06	1,7466E-27
685	41149	-1,58	4,53E-21	785	122909	-1,46	4,24E-23	885	72012	-1,35	1,85E-28	985	123777	-1,26	5,64E-25	1085	21363	-1,15	1,04E-18	1185	109536	-1,06	5,6033E-14
686	107784	-1,58	1,06E-52	786	63503	-1,46	6,32E-14	886	123588	-1,35	1,22E-26	986	33692	-1,26	2,57E-28	1086	3596	-1,15	1,51E-03	1186	54144	-1,06	5,746E-13
687	103033	-1,58	2,35E-02	787	121773	-1,46	7,36E-24	887	120978	-1,35	1,35E-33	987	110152	-1,26	1,46E-16	1087	121471	-1,14	2,63E-26	1187	111764	-1,06	0,00107629
688	120821	-1,58	1,80E-47	788	120747	-1,46	3,24E-10	888	111307	-1,35	3,66E-16	988	81896	-1,26	2,49E-34	1088	72615	-1,14	3,28E-29	1188	66895	-1,06	1,0264E-13
689	62198	-1,58	7,19E-30	789	59354	-1,46	1,19E-16	889	55123	-1,35	1,99E-25	989	66210	-1,26	3,16E-08	1089	82334	-1,14	1,38E-24	1189	66563	-1,05	8,3571E-12
690	4514	-1,58	1,47E-17	790	49373	-1,46	4,55E-19	890	60784	-1,35	5,74E-06	990	120833	-1,25	1,32E-45	1090	63180	-1,14	8,22E-09	1190	23298	-1,05	1,9941E-19
691	75646	-1,57	1,91E-36	791	52763	-1,46	4,66E-56	891	1959	-1,35	7,64E-06	991	72581	-1,25	2,22E-05	1091	27085	-1,14	2,61E-14	1191	104261	-1,05	0,00596957
692	3001	-1,57	1,84E-32	792	71304	-1,46	7,69E-39	892	64420	-1,35	1,65E-25	992	75169	-1,25	2,99E-29	1092	120114	-1,14	3,85E-17	1192	79644	-1,05	1,6246E-08
693	57185	-1,57	2,61E-04	793	68184	-1,46	5,57E-20	893	79111	-1,34	1,56E-29	993	111807	-1,25	5,52E-07	1093	64778	-1,14	5,13E-11	1193	60616	-1,05	2,3478E-05
694	65685	-1,57	1,04E-20	794	112532	-1,46	3,52E-03	894	102837	-1,34	2,53E-28	994	124113	-1,25	1,54E-34	1094	52839	-1,14	7,31E-17	1194	111682	-1,05	0,00025803
695	59988	-1,57	2,99E-31	795	41248	-1,46	6,98E-12	895	62470	-1,34	1,98E-40	995	81139	-1,25	2,18E-15	1095	124314	-1,14	6,89E-23	1195	123090	-1,05	6,5258E-14
696	58404	-1,57	3,98E-32	796	103065	-1,46	8,61E-03	896	56896	-1,34	1,42E-13	996	111124	-1,24	7,20E-06	1096	46238	-1,14	4,59E-18	1196	60564	-1,05	4,4213E-15
697	54535	-1,57	1,45E-26	797	112019	-1,46	1,16E-06	897	2100	-1,34	7,28E-24	997	3856	-1,24	2,68E-14	1097	58651	-1,14	8,04E-24	1197	119875	-1,05	3,2851E-07
698	120012	-1,57	3,04E-57	798	112081	-1,46	6,34E-39	898	81570	-1,34	1,53E-30	998	53446	-1,24	1,04E-15	1098	122376	-1,14	1,04E-15	1198	120952	-1,05	1,8677E-16
699	70960	-1,56	7,24E-11	799	78695	-1,45	3,05E-39	899	105167	-1,34	1,81E-02	999	122956	-1,24	2,41E-28	1099	22737	-1,14	1,65E-25	1199	66804	-1,04	1,8505E-12
700	123850	-1,56	1,67E-49	800	80211	-1,45	3,38E-50	900	76939	-1,34	1,33E-38	1000	108778	-1,24	1,27E-23	1100	109249	-1,13	1,11E-02	1200	72287	-1,04	2,1745E-05

Supplementary Table S12 (continued): FULL list of DEGs from RNA-Seq data for Δ*ron1* (jgi|Trire2|79673) grown on GlcNAc and compared to control (WT grown on GlcNAc).

Differentially expressed genes (DEGs) were defined by fold change ≥|1.0| and a adjusted p-value (padj) of < 0.05 found by DESeq2 (RStudio 2022.07.2+576)

	Identifier (TRIREDRAFT)	log2fc	padj
1201	106797	-1,04	5,96E-13
1202	62502	-1,04	1,79E-09
1203	80997	-1,04	9,29E-18
1204	58363	-1,04	1,72E-13
1205	77613	-1,04	5,60E-30
1206	61815	-1,04	1,10E-14
1207	81460	-1,04	3,88E-07
1208	109296	-1,04	7,24E-04
1209	74258	-1,04	1,12E-18
1210	104744	-1,04	1,48E-11
1211	60855	-1,03	6,10E-12
1212	68660	-1,03	2,17E-09
1213	108144	-1,03	8,61E-03
1214	78864	-1,03	3,15E-10
1215	55508	-1,03	1,17E-07
1216	67840	-1,03	2,42E-09
1217	123636	-1,03	5,50E-19
1218	63709	-1,03	1,66E-12
1219	3007	-1,03	6,24E-14
1220	109346	-1,02	2,05E-08
1221	82085	-1,02	2,59E-20
1222	82105	-1,02	1,40E-09
1223	81756	-1,02	5,07E-16
1224	121135	-1,02	3,43E-02
1225	103366	-1,02	7,31E-04
1226	57280	-1,02	5,22E-03
1227	64385	-1,02	2,12E-13
1228	110415	-1,01	4,51E-08
1229	1993	-1,01	9,59E-12
1230	75992	-1,01	2,08E-08
1231	122175	-1,01	1,25E-11
1232	121464	-1,01	8,91E-12
1233	68107	-1,01	4,55E-19
1234	59068	-1,01	2,74E-17
1235	65496	-1,01	3,25E-16
1236	62086	-1,01	5,71E-10
1237	74983	-1,01	6,46E-13
1238	73829	-1,00	1,83E-12
1239	56344	-1,00	1,72E-10
1240	81001	-1,00	1,99E-13
1241	68876	-1,00	1,66E-09
1242	78288	-1,00	7,14E-12

Supplementary Table S12 (continued): FULL list of DEGs from RNA-Seq data for Δ*ron1* (jgi|Trire2|79673) grown on GlcNAc and compared to control (WT grown on GlcNAc).

Differentially expressed genes (DEGs) were defined by fold change ≥|1.0| and a adjusted p-value (padj) of < 0.05 found by DESeq2 (RStudio 2022.07.2+576)

	Identifier (TRIREDRAFT)	log2fc	padj		Identifier (TRIREDRAFT)	log2fc	padj		Identifier (TRIREDRAFT)	log2fc	padj		Identifier (TRIREDRAFT)	log2fc	padj		Identifier (TRIREDRAFT)	log2fc	padj		Identifier (TRIREDRAFT)	log2fc	padj
1	112568	10,64	2,88E-18	101	57749	4,20	5,23E-110	201	65162	3,09	4,23E-58	301	60889	2,54	2,01E-16	401	79271	2,14	3,58E-08	501	108201	1,83	1,63E-24
2	44278	10,14	0,00E+00	102	68230	4,18	2,62E-178	202	66657	3,09	1,38E-65	302	72137	2,54	1,78E-10	402	120473	2,13	3,48E-02	502	107137	1,83	3,68E-40
3	103136	9,92	2,20E-15	103	67541	4,17	3,48E-07	203	103131	3,08	2,98E-07	303	121743	2,54	4,16E-48	403	61114	2,13	6,94E-44	503	43893	1,83	6,78E-51
4	121441	9,55	2,27E-58	104	110471	4,16	1,23E-31	204	31248	3,06	3,34E-219	304	104551	2,53	5,21E-09	404	109779	2,13	4,13E-02	504	62439	1,83	6,34E-47
5	81087	8,66	1,04E-107	105	109811	4,15	2,07E-103	205	48747	3,06	1,17E-127	305	59205	2,53	5,27E-15	405	66583	2,12	5,51E-34	505	104867	1,82	5,30E-04
6	59689	8,62	8,56E-22	106	104077	4,15	8,84E-54	206	60374	3,05	4,40E-50	306	108672	2,53	7,91E-03	406	121396	2,12	1,66E-35	506	74070	1,82	4,28E-60
7	111915	7,93	5,94E-157	107	107340	4,15	1,75E-224	207	109234	3,05	3,00E-18	307	105763	2,51	6,13E-51	407	110167	2,11	1,81E-02	507	56934	1,82	1,70E-04
8	81275	7,74	2,79E-54	108	79921	4,15	3,11E-113	208	112126	3,04	4,25E-85	308	105260	2,51	5,95E-21	408	82208	2,11	8,88E-09	508	67030	1,82	2,22E-02
9	56289	7,71	2,51E-09	109	58823	4,13	4,11E-02	209	82619	3,04	2,17E-119	309	49589	2,51	6,27E-132	409	102785	2,11	2,52E-11	509	58952	1,81	8,98E-03
10	123234	7,56	2,37E-27	110	68019	4,13	1,72E-58	210	4941	3,04	6,09E-122	310	67377	2,51	1,81E-58	410	108477	2,10	2,20E-77	510	123888	1,81	5,30E-44
11	109361	7,31	3,48E-08	111	72183	4,10	4,65E-103	211	103886	3,04	1,08E-10	311	69611	2,51	2,39E-41	411	4454	2,10	6,98E-06	511	60187	1,81	1,30E-03
12	105279	7,27	7,75E-08	112	111849	4,09	1,71E-48	212	70021	3,04	4,55E-06	312	21873	2,50	1,96E-46	412	68254	2,10	2,35E-83	512	110414	1,81	2,91E-05
13	108349	7,22	5,86E-08	113	65986	4,08	1,16E-112	213	107279	3,04	1,68E-69	313	4726	2,50	6,89E-73	413	80920	2,10	2,68E-05	513	65950	1,80	2,85E-26
14	69736	7,07	9,55E-11	114	61055	4,07	1,48E-34	214	106575	3,04	3,38E-38	314	67579	2,50	1,40E-21	414	63914	2,10	3,30E-23	514	65854	1,80	2,39E-26
15	81536	7,07	1,56E-10	115	110220	4,07	2,51E-32	215	60445	3,00	7,17E-05	315	4308	2,49	2,05E-12	415	61127	2,10	1,49E-30	515	69557	1,80	4,31E-58
16	111932	6,97	3,18E-22	116	3049	4,06	7,12E-165	216	75290	2,98	5,46E-09	316	69245	2,49	1,49E-42	416	64784	2,10	1,11E-81	516	2916	1,80	2,41E-35
17	122495	6,97	2,97E-07	117	81430	4,04	1,58E-154	217	119552	2,98	1,08E-04	317	49366	2,49	3,53E-38	417	103393	2,09	3,06E-03	517	64720	1,78	3,06E-02
18	123550	6,80	3,68E-39	118	107494	4,03	1,07E-100	218	70334	2,98	2,89E-90	318	104073	2,48	1,10E-04	418	64996	2,09	3,12E-02	518	121418	1,78	3,16E-02
19	119790	6,80	5,43E-250	119	123029	4,02	2,17E-204	219	76227	2,97	3,53E-123	319	106181	2,47	1,01E-50	419	27770	2,09	4,22E-16	519	119896	1,78	3,01E-20
20	46794	6,79	0,00E+00	120	106043	4,00	1,38E-53	220	106697	2,96	9,14E-03	320	122963	2,47	4,20E-38	420	45445	2,08	3,89E-90	520	105771	1,78	2,64E-31
21	111138	6,75	2,83E-152	121	112115	3,97	2,33E-08	221	68508	2,95	6,33E-53	321	81517	2,47	8,77E-96	421	2211	2,07	1,14E-11	521	66611	1,78	1,14E-02
22	120837	6,64	0,00E+00	122	104171	3,94	1,83E-02	222	111129	2,94	5,07E-78	322	105408	2,47	9,92E-15	422	28185	2,07	6,22E-08	522	65718	1,78	3,31E-04
23	33387	6,52	2,43E-55	123	107960	3,93	4,01E-03	223	57857	2,93	8,41E-18	323	68036	2,47	5,23E-57	423	4430	2,07	7,04E-14	523	64018	1,77	7,38E-16
24	80659	6,41	7,69E-17	124	121127	3,93	1,83E-07	224	122198	2,92	4,38E-57	324	64656	2,47	9,42E-35	424	123865	2,07	2,65E-51	524	112134	1,77	1,46E-13
25	109235	6,41	1,02E-129	125	69857	3,92	5,81E-29	225	103335	2,91	1,34E-03	325	2489	2,46	1,45E-11	425	110709	2,05	3,39E-02	525	30166	1,77	1,18E-11
26	107867	6,39	1,22E-05	126	104322	3,91	7,01E-219	226	72379	2,91	6,21E-09	326	22912	2,46	6,81E-61	426	27395	2,05	3,94E-34	526	23408	1,76	1,57E-35
27	124051	6,35	4,21E-32	127	124115	3,91	4,96E-113	227	56830	2,91	3,90E-46	327	65741	2,44	3,20E-76	427	70803	2,05	3,30E-11	527	122153	1,76	2,54E-38
28	120176	6,33	2,05E-262	128	67639	3,91	3,48E-24	228	81843	2,90	1,66E-44	328	57179	2,44	3,87E-02	428	78713	2,05	3,85E-52	528	71072	1,76	5,89E-04
29	70800	6,33	8,33E-06	129	55630	3,86	2,39E-66	229	4124	2,90	2,45E-08	329	48211	2,44	1,08E-35	429	62053	2,05	2,63E-13	529	104549	1,76	2,97E-43
30	72632	6,23	6,91E-105	130	22210	3,86	6,39E-73	230	111395	2,89	1,25E-57	330	60370	2,44	5,69E-05	430	80149	2,05	1,15E-61	530	121251	1,76	8,74E-29
31	51365	6,12	1,89E-298	131	68705	3,86	5,91E-170	231	23062	2,89	2,36E-105	331	124177	2,44	5,40E-41	431	66175	2,04	2,00E-28	531	41208	1,76	5,37E-30
32	66819	6,12	3,65E-173	132	122556	3,85	8,69E-09	232	62611	2,88	1,94E-114	332	62172	2,44	9,16E-22	432	59771	2,04	6,41E-04	532	68843	1,76	3,45E-31
33	123978	6,10	1,22E-267	133	123241	3,85	2,31E-179	233	109335	2,84	3,42E-20	333	57647	2,44	4,11E-39	433	5807	2,04	2,29E-06	533	108833	1,75	4,51E-41
34	103451	6,08	1,12E-299	134	58848	3,83	8,22E-05	234	65055	2,84	1,70E-55	334	110440	2,43	6,96E-21	434	21422	2,03	1,88E-38	534	55561	1,75	1,66E-33
35	121164	6,04	0,00E+00	135	111357	3,82	2,35E-103	235	122007	2,84	7,67E-92	335	59322	2,43	2,89E-08	435	74453	2,02	5,21E-05	535	124092	1,75	9,41E-03
36	107869	6,04	6,20E-19	136	111442	3,82	2,59E-85	236	123095	2,84	5,53E-40	336	111957	2,43	3,83E-10	436	3449	2,02	6,07E-34	536	54239	1,75	2,93E-27
37	73897	6,00	2,71E-40	137	65153	3,81	1,82E-111	237	5502	2,84	2,85E-06	337	21412	2,41	3,39E-77	437	69066	2,01	1,14E-07	537	70351	1,75	7,79E-31
38	66776	5,99	4,02E-05	138	111527	3,80	3,99E-83	238	61293	2,83	4,68E-27	338	61536	2,41	6,47E-05	438	105027	2,01	1,83E-05	538	56064	1,75	1,80E-54
39	23415	5,99	0,00E+00	139	111236	3,78	1,10E-58	239	120079	2,83	3,63E-08	339	63526	2,41	8,19E-42	439	123989	2,00	7,66E-19	539	103668	1,75	1,03E-15
40	56646	5,91	4,35E-07	140	103799	3,77	2,14E-27	240	111897	2,83	2,12E-13	340	78828	2,40	4,14E-41	440	53475	2,00	2,48E-17	540	47930	1,74	2,45E-45
41	74563	5,90	2,66E-283	141	69811	3,75	7,45E-185	241	76155	2,81	1,53E-164	341	111362	2,39	1,38E-71	441	69972	2,00	1,08E-36	541	70961	1,74	3,39E-29
42	106537	5,86	2,10E-05	142	22093	3,74	8,82E-251	242	122582	2,78	5,27E-96	342	106538	2,39	5,85E-27	442	78833	1,99	4,34E-31	542	51562	1,74	2,38E-63
43	59843	5,80	1,14E-85	143	122874	3,74	2,27E-80	243	79726	2,78	1,44E-19	343	120688	2,38	1,04E-50	443	123955	1,99	5,50E-30	543	67751	1,73	7,90E-23
44	62872	5,70	8,12E-190	144	59388	3,74	2,56E-27	244	107935	2,78	3,85E-06	344	5924	2,38	1,62E-49	444	109746	1,99	3,02E-57	544	79405	1,73	2,43E-49
45	111890	5,70	4,89E-05	145	120698	3,70	3,94E-208	245	60988	2,77	1,79E-16	345	110330	2,38	4,74E-02	445	122374	1,98	7,04E-63	545	34221	1,73	8,19E-05
46	68427	5,65	1,64E-152	146	59952	3,67	4,49E-64	246	105269	2,77	2,39E-35	346	106676	2,37	9,20E-16	446	58068	1,96	8,16E-48	546	59028	1,73	2,49E-08
47	53029	5,65	2,13E-04	147	108145	3,65	1,85E-37	247	106168	2,76	3,46E-02	347	49753	2,37	2,61E-43	447	123914	1,96	1,20E-41	547	2322	1,73	6,15E-18
48	121495	5,62	1,33E-17	148	123639	3,65	3,81E-11	248	64397	2,75	1,63E-142	348	106879	2,36	8,62E-33	448	108540	1,96	2,97E-06	548	67538	1,72	5,38E-10
49	53079	5,56	5,59E-60	149	60329	3,64	2,19E-27	249	104084	2,75	5,96E-42	349	3363	2,35	7,59E-63	449	123723	1,95	1,06E-66	549	110214	1,72	1,37E-27
50	107868	5,56	3,15E-04	150	59382	3,64	2,14E-182	250	3716	2,75	1,56E-113	350	112247	2,35	3,78E-17	450	66394	1,95	6,00E-54	550	123922	1,71	5,37E-47
51	55881	5,52	3,01E-45	151	104277	3,63	1,53E-54	251	5345	2,75	3,25E-17	351	49048	2,35	4,14E-52	451	119823	1,95	3,19E-49	551	59396	1,71	1,43E-02
52	112128	5,50	4,18E-06	152	123226	3,61	3,10E-127	252	49946	2,74	6,93E-115	352	76696	2,35	1,64E-78	452	78626	1,95	7,91E-76	552	62747	1,71	5,29E-05
53	80058	5,47	1,16E-16	153	121482	3,60	2,27E-196	253	72339	2,74	3,26E-109	353	3718	2,34	1,27E-18	453	123911	1,95	1,42E-20	553	122666	1,71	1,60E-44
54	105150	5,41	1,21E-04	154	105924	3,56	2,44E-11	254	106885	2,74	1,37E-43	354	75027	2,34	6,86E-65	454	81014	1,95	7,87E-34	554	76734	1,70	3,77E-24
55	50104	5,40	2,52E-32	155	2223	3,55	1,43E-108	255	72524	2,72	5,94E-14	355	119789	2,33	7,70E-117	455	108885	1,94	1,15E-39	555	41518	1,70	2,33E-49
56	111729	5,39	4,92E-06	156	70933	3,55	1,60E-15	256	124222	2,72	4,48E-98	356	66544	2,33	2,51E-03	456	30668	1,94	1,48E-07	556	109277	1,70	1,12E-02
57	120017	5,35	0,00E+00	157	58857	3,53	7,06E-10	257	73005	2,72	7,67E-03	357	59642	2,33	2,47E-62	457	103694	1,94	9,89E-51	557	27697	1,69	2,52E-04
58	121883	5,32	3,58E-43	158	65915	3,52	7,29E-144	258	47268	2,71	3,65E-68	358	56684	2,33	5,40E-10	458	105787	1,94	2,85E-02	558	22252	1,69	7,35E-64
59	34493	5,18	4,43E-235	159	68590	3,52	1,84E-52	259	29993	2,71	1,22E-64	359	123818	2,33	3,42E-04	459	123473	1,94	4,60E-35	559	104394	1,69	2,86E-02
60	102820	5,12	3,54E-36	160	106330	3,50	3,16E-162	260	121968	2,71	4,19E-57	360	105237	2,32	9,46E-03	460	65229	1,93	2,70E-44	560	70808	1,69	3,00E-17
61	106129	5,04	2,53E-150	161	31134	3,49	8,21E-181	261	81778	2,71	1,45E-105	361	46002	2,32	6,73E-113	461	3405	1,93	7,74E-22	561	70319	1,69	4,01E-05
62	111405	5,02	5,43E-07	162	58282	3,49	0,00E+00	262	67938	2,69	7,48E-14	362	81668	2,32	3,39E-22	462	104140	1,93	1,35E-02	562	65677	1,68	1,09E-17
63	103423	4,99	3,99E-03	163	58701	3,48	4,40E-04	263	26163	2,69	2,85E-04	363	106706	2,31	2,65E-79	463	54166	1,92	1,81E-28	563	44366	1,68	2,51E-07
64	109122	4,98	6,67E-12	164	3914	3,47	1,79E-02	264	30758	2,69	1,31E-02	364	102966	2,30	1,45E-40	464	54550	1,92	1,06E-35	564	58511	1,68	3,77E-42
65	57643	4,97	2,56E-113	165	38640	3,46	4,16E-154	265	80875	2,69	6,77E-118	365	110238	2,30	2,68E-44	465	121717	1,92	1,16E-59	565	122527	1,68	3,19E-25
66	76897	4,97	0,00E+00	166	56687	3,44	2,75E-05	266	78683	2,68	2,62E-08	366	77299	2,29	1,47E-20	466	70956	1,92	3,99E-07	566	57592	1,68	5,45E-23
67	54219	4,97	3,12E-03	167	102999	3,41	1,75E-38	267	121735	2,68	2,06E-59	367	5270	2,28	3,99E-30	467	104393	1,92	9,19E-14	567	70842	1,68	1,33E-08
68	70341	4,93	8,47E-19	168	54226	3,41	3,44E-52	268	82628	2,66	2,25E-02	368	112094	2,28	2,15E-28	468	65198	1,91	1,32E-07	568	108075	1,67	0,014064
69	56314	4,93	5,13E-44	169	54962	3,39	4,42E-26	269	121306	2,65	9,09E-82	369	55887	2,27	3,06E-40	469	110261	1,91	1,94E-03	569	30084	1,67	1,14E-10
70	103009	4,92	1,09E-12	170	108340	3,39	3,11E-13	270	79606	2,65	2,66E-153	370	109673	2,27	1,67E-11	470	78552	1,91	3,27E-32	570	60177	1,66	1,15E-10
71	123274	4,91	5,54E-45	171	60635	3,38	1,25E-06	271	80340	2,65	5,55E-69	371	111514	2,27	7,62E-03	471	58561	1,91	2,52E-40	571	120106	1,66	2,01E-55
72	108671	4,82	1,25E-03	172	105765	3,38	2,24E-57	272	38080	2,64	2,93E-59	372	48080	2,26	3,91E-56	472	108157	1,90	3,17E-32	572	119931	1,66	1,37E-44
73	64181	4,78	2,72E-06	173	47286	3,38	3,22E-135	273	67035	2,64	3,67E-93	373	103825	2,25	1,35E-08	473	53611	1,90	1,14E-07	573	109677	1,65	2,21E-21
74	50323	4,71	4,04E-10	174	57322	3,35	4,55E-14	274	112523	2,62	2,56E-02	374	79202	2,25	3,54E-18	474	57671	1,90	3,15E-35	574	56625	1,65	3,09E-05
75	77093	4,68	5,17E-24	175	120784	3,33	7,07E-79	275	103275	2,62	2,87E-51	375	52520	2,24	3,63E-80	475	64667	1,90	5,54E-08	575	108835	1,65	1E-28
76	60418	4,67	8,71E-11	176	67084	3,32	6,52E-19	276	110493	2,62	6,79E-17	376	108870	2,23	2,52E-11	476	80026	1,89	1,14E-47	576	57370	1,65	2,32E-38
77	61374	4,66	1,34E-66	177	76065	3,31	3,99E-83	277	80096	2,61	6,55E-58	377	47432	2,23	1,45E-67	477	66929	1,89	4,95E-04	577	106591	1,64	0,013073
78	22459	4,63	2,45E-231	178	123510	3,30	1,97E-53	278	5337	2,61	1,91E-39	378	46266	2,22	3,76E-36	478	111923	1,89	1,01E-03	578	70932	1,64	8,85E-35
79	70373	4,60	4,65E-142	179	122614	3,28	3,14E-151	279	109538	2,61	4,72E-02	379	103108	2,22	1,30E-77	479	5347	1,89	1,36E-07	579	31367	1,64	1,6E-18
80	122972	4,60	3,07E-198	180	65744	3,24	6,33E-29	280	72611	2,61	7,59E-117	380	57975	2,22	4,18E-35	480	22510	1,89	1,55E-51	580	107947	1,64	1,06E-46
81	74745	4,59	0,00E+00	181	103129	3,23	3,27E-03	281	73110	2,61	3,95E-06	381	54954	2,21	1,97E-30	481	5942	1,88	2,71E-28	581	123658	1,64	1,15E-39
82	58899	4,55	0,00E+00	182	110855	3,23	2,47E-56	282	121308	2,61	1,00E-49	382	65215	2,21	4,42E-80	482	82403	1,88	3,26E-47	582	111446	1,64	3,39E-34
83	42152	4,50	1,12E-08	183	57914	3,19	1,48E-110	283	119860	2,60	1,36E-36	383	120765	2,21	3,28E-05	483	27259	1,88	1,04E-07	583	65164	1,63	3,29E-16
84	62693	4,48	2,62E-229	184	56328	3,18	3,47E-10	284	79835	2,60	2,79E-97	384	107669	2,20	7,25E-16	484	22785	1,87	2,49E-40	584	70092	1,63	8,5E-15
85	67692	4,46	1,90E-06	185	80645	3,18	2,16E-17	285	103614	2,60	3,24E-07	385	71059	2,19	1,37E-16	485	52073	1,87	8,71E-58	585	76822	1,63	2,84E-34
86	68122	4,43	5,69E-258	186	56587	3,15	6,89E-13	286	111094	2,60	9,82E-05	386	123771	2,19	2,48E-42	486	5107	1,87	7,16E-07	586	22694	1,63	8,2E-46
87	122087	4,39	1,33E-78	187	50894	3,15	3,13E-34	287	3739	2,59	4,40E-33	387	65735	2,19	1,75E-38	487	44700	1,87	1,41E-40	587	60517	1,63	5,61E-09
88	59391	4,38	3,67E-215	188	103130	3,15	6,88E-03	288	71080	2,59	1,58E-58	388	69468	2,19	6,76E-23	488	104333	1,87	1,21E-18	588	120912	1,63	4,42E-25
89	55802	4,37	1,19E-239	189	58381	3,15	3,98E-226	289	4109	2,59	8,34E-45	389	109146	2,19	5,80E-08	489	56432	1,86	4,85E-67	589	123199	1,63	1,84E-11
90	67752	4,36	0,00E+00	190	50616	3,15	9,92E-136	290	105816	2,58	3,97E-11	390	44175	2,18	2,46E-95	490	124169	1,86	2,76E-81	590	122736	1,63	2,7E-05
91	122375	4,35	6,47E-04	191	103458	3,14	5,02E-04	291	78611	2,58	2,31E-87	391	82039	2,17	9,70E-55	491	57555	1,86	2,92E-04	591	109960	1,62	3,01E-14
92	60116	4,32	1,51E-34	192	65891	3,14	2,34E-88	292	112031	2,58	7,24E-36	392	64937	2,17	4,06E-07	492	53428	1,85	2,71E-27	592	106625	1,62	8,35E-05
93	103695	4,29	4,02E-69	193	76852	3,14	6,80E-09	293	60565	2,58	3,31E-45	393	27181	2,17	2,93E-30	493	82626	1,85	1,18E-19	593	72993	1,62	0,029781
94	3717	4,28	3,75E-81	194	104215	3,13	4,09E-18	294	69613	2,57	9,34E-09	394	109078	2,17	3,31E-61	494	103959	1,85	9,41E-07	594	68615	1,62	1,19E-37
95	72627	4,27	1,50E-66	195	123261	3,12	7,83E-05	295	122108	2,57	2,37E-64	395	51415	2,17	1,87E-09	495	60490	1,84	1,62E-31	595	57098	1,61	9,27E-47
96	123649	4,25	2,30E-19	196	56470	3,10	4,84E-116	296	55636	2,57	2,71E-70	396	104606	2,17	3,25E-39	496	35777	1,84	4,93E-33	596	110880	1,61	3,98E-05
97	110757	4,24	5,81E-18	197	103059	3,10	1,06E-03	297	60085	2,56	3,67E-11	397	121156	2,16	2,23E-37	497	43397	1,84	2,30E-14	597	47603	1,61	7,51E-06
98	77743	4,23	1,99E-20	198	56996	3,10	4,23E-02	298	53961	2,56	2,59E-16	398	64274	2,16	3,45E-39	498	23367	1,84	1,02E-70	598	57860	1,61	2,51E-35
99	58717	4,22	3,61E-02	199	55709	3,09	4,82E-77	299	58802	2,56	4,68E-04	399	110455	2,15	3,37E-09	499	5366	1,84	3,30E-52	599	71170	1,61	1,21E-20
100	72526	4,21	7,49E-37	200	66753	3,09	2,27E-04	300	74198	2,55	1,41E-24	400	56003	2,14	4,68E-33	500	61212	1,84	6,07E-06	600	107848	1,61	5,33E-13

Supplementary Table S12 (continued): FULL list of DEGs from RNA-Seq data for Δ*ron1* (jgi|Trire2|79673) grown on GlcNAc and compared to control (WT grown on GlcNAc)

Differentially expressed genes (DEGs) were defined by fold change ≥|1.0| and a adjusted p-value (padj) of < 0.05 found by DESeq2 (RStudio 2022.07.2+576)

	Identifier (TRIREDRAFT)	log2fc	padj
601	82374	1,61	1,17E-15
602	105844	1,60	3,09E-08
603	122131	1,60	8,36E-25
604	111955	1,60	1,21E-05
605	55990	1,59	3,07E-21
606	4027	1,59	8,92E-03
607	110877	1,59	7,17E-20
608	74601	1,59	4,28E-45
609	81553	1,58	2,08E-19
610	103801	1,57	1,52E-02
611	65739	1,57	8,92E-03
612	65141	1,57	6,61E-14
613	65965	1,57	9,93E-40
614	106877	1,57	1,68E-02
615	103034	1,57	4,12E-08
616	82317	1,57	1,17E-20
617	49295	1,57	8,80E-40
618	110878	1,57	3,71E-08
619	66592	1,56	3,82E-04
620	5656	1,56	3,35E-37
621	59801	1,56	2,29E-19
622	65190	1,56	1,08E-04
623	72091	1,56	4,91E-46
624	80654	1,56	6,22E-08
625	106130	1,56	4,18E-54
626	111135	1,56	1,88E-09
627	80091	1,55	4,39E-22
628	121608	1,55	1,18E-17
629	29346	1,55	2,16E-04
630	59364	1,55	4,54E-07
631	69026	1,55	6,10E-20
632	119576	1,55	4,27E-21
633	119839	1,55	9,12E-25
634	108940	1,54	5,04E-34
635	45717	1,54	1,78E-36
636	73516	1,54	1,00E-36
637	120943	1,54	6,07E-22
638	120381	1,53	6,04E-41
639	105888	1,53	4,03E-05
640	72567	1,53	2,80E-04
641	67494	1,52	2,23E-09
642	34252	1,52	3,70E-12
643	36703	1,52	5,62E-14
644	4480	1,52	5,32E-28
645	75742	1,52	8,37E-39
646	119956	1,52	9,24E-51
647	56095	1,52	1,37E-03
648	70859	1,52	2,03E-46
649	80756	1,52	1,06E-42
650	121475	1,51	1,41E-56
651	81511	1,51	2,13E-15
652	69316	1,51	1,14E-06
653	122974	1,51	3,28E-23
654	102743	1,50	1,82E-32
655	68000	1,50	4,16E-14
656	103813	1,50	3,09E-10
657	121877	1,49	3,67E-02
658	21817	1,49	2,03E-34
659	81097	1,49	4,95E-22
660	123278	1,49	2,85E-05
661	105980	1,49	1,62E-10
662	32849	1,49	5,43E-16
663	61304	1,49	4,16E-06
664	122140	1,49	4,21E-04
665	74971	1,48	6,77E-14
666	68130	1,48	1,14E-29
667	60378	1,48	4,93E-04
668	21758	1,48	5,73E-17
669	63653	1,48	1,23E-07
670	72076	1,48	1,07E-15
671	112085	1,48	1,13E-04
672	70542	1,48	1,26E-18
673	70108	1,48	1,42E-03
674	21659	1,47	2,97E-47
675	108557	1,47	1,62E-07
676	123686	1,47	3,89E-26
677	2231	1,47	5,90E-35
678	104390	1,47	1,56E-23
679	120998	1,47	1,47E-64
680	106829	1,47	6,20E-29
681	107035	1,46	1,94E-03
682	105979	1,46	6,90E-05
683	66795	1,46	1,06E-09
684	82544	1,46	3,61E-34
685	123699	1,46	7,60E-26
686	109275	1,45	4,74E-22
687	3464	1,45	8,92E-44
688	60270	1,45	1,42E-28
689	68455	1,45	6,56E-44
690	80973	1,45	3,74E-18
691	109311	1,45	4,43E-03
692	104511	1,44	2,54E-09
693	121439	1,44	9,06E-07
694	62805	1,43	8,97E-03
695	78499	1,43	8,05E-27
696	122212	1,43	2,70E-26
697	119859	1,43	2,42E-12
698	53777	1,42	2,20E-10
699	107339	1,42	3,70E-03
700	32364	1,42	3,95E-10

	Identifier (TRIREDRAFT)	log2fc	padj
701	78391	1,42	5,90E-21
702	122187	1,41	1,97E-05
703	109147	1,41	3,35E-02
704	124059	1,41	5,90E-11
705	43961	1,41	1,78E-33
706	110158	1,41	9,66E-03
707	105810	1,41	6,10E-05
708	59095	1,41	7,99E-15
709	59649	1,41	1,20E-03
710	67718	1,41	1,51E-15
711	41663	1,41	2,09E-10
712	59338	1,40	1,53E-06
713	108591	1,40	1,95E-06
714	32293	1,40	4,32E-05
715	67983	1,39	5,05E-31
716	68961	1,39	1,04E-04
717	60422	1,39	8,70E-14
718	105863	1,39	7,16E-25
719	65547	1,39	1,24E-06
720	42848	1,39	9,26E-20
721	70397	1,39	2,40E-24
722	107914	1,39	1,65E-02
723	81522	1,39	5,01E-04
724	110658	1,38	4,22E-28
725	22004	1,38	5,89E-35
726	107704	1,38	1,04E-05
727	26160	1,38	2,11E-05
728	123283	1,38	5,36E-08
729	3481	1,38	7,89E-23
730	120873	1,38	3,01E-02
731	80252	1,37	9,75E-26
732	121889	1,37	5,58E-31
733	54659	1,37	5,67E-04
734	64172	1,37	1,46E-13
735	120008	1,37	2,76E-37
736	53373	1,37	9,65E-12
737	108819	1,37	3,53E-04
738	57776	1,37	9,18E-06
739	60282	1,37	5,67E-21
740	56418	1,36	3,13E-04
741	103147	1,36	5,55E-23
742	121500	1,36	1,97E-29
743	104364	1,36	4,39E-38
744	62556	1,35	8,44E-14
745	44178	1,35	9,23E-16
746	5275	1,35	6,47E-03
747	108553	1,35	6,27E-09
748	122975	1,35	2,58E-02
749	122102	1,35	2,05E-09
750	60232	1,34	1,37E-14
751	104599	1,34	9,51E-26
752	119933	1,33	1,53E-12
753	55077	1,33	4,58E-13
754	120120	1,33	1,64E-14
755	120189	1,33	1,76E-30
756	60052	1,33	5,97E-10
757	107507	1,33	2,07E-23
758	23146	1,33	1,28E-20
759	22661	1,33	4,52E-35
760	70630	1,33	3,90E-19
761	122856	1,32	1,48E-33
762	61278	1,32	3,23E-04
763	106154	1,32	3,09E-02
764	104838	1,32	1,41E-19
765	62576	1,32	6,10E-03
766	107012	1,32	4,15E-20
767	103446	1,31	2,99E-14
768	102908	1,31	3,64E-20
769	70894	1,31	4,11E-04
770	74129	1,31	9,12E-22
771	67446	1,31	6,66E-27
772	75998	1,31	5,71E-32
773	55407	1,31	4,91E-15
774	74057	1,30	3,14E-22
775	123120	1,30	1,90E-20
776	104925	1,30	1,28E-14
777	121653	1,30	3,14E-06
778	124338	1,30	1,67E-04
779	2392	1,30	1,67E-41
780	49970	1,30	6,71E-34
781	64834	1,30	1,91E-08
782	76141	1,30	1,90E-18
783	67484	1,30	6,71E-22
784	73179	1,29	2,22E-09
785	58587	1,29	8,16E-29
786	110768	1,29	1,83E-12
787	58387	1,29	1,89E-28
788	60018	1,29	9,20E-07
789	119534	1,28	3,14E-16
790	123354	1,28	5,75E-31
791	78799	1,28	3,23E-38
792	107670	1,28	1,39E-25
793	81110	1,28	7,76E-30
794	107111	1,28	8,68E-20
795	103498	1,27	5,34E-12
796	122284	1,27	7,26E-34
797	21960	1,27	7,09E-34
798	22484	1,27	1,48E-25
799	53500	1,27	1,26E-23
800	47479	1,27	2,76E-21

	Identifier (TRIREDRAFT)	log2fc	padj
801	5598	1,26	9,61E-33
802	112516	1,26	2,04E-11
803	50593	1,26	2,82E-12
804	105330	1,26	9,62E-04
805	66854	1,26	1,44E-04
806	62285	1,26	3,96E-15
807	107260	1,26	7,98E-26
808	108357	1,26	7,38E-23
809	47315	1,26	3,00E-15
810	123827	1,26	1,81E-17
811	102487	1,25	3,49E-11
812	60897	1,25	3,58E-26
813	121285	1,25	3,08E-18
814	70172	1,25	1,84E-05
815	54502	1,25	1,62E-19
816	35186	1,24	1,36E-03
817	72086	1,24	1,05E-06
818	45688	1,24	1,49E-19
819	120404	1,24	3,25E-07
820	79169	1,24	6,33E-17
821	2703	1,23	1,35E-08
822	53561	1,23	2,06E-05
823	54846	1,23	5,49E-09
824	102735	1,23	2,85E-20
825	63202	1,23	1,50E-10
826	103482	1,23	9,34E-09
827	112520	1,23	9,54E-05
828	23190	1,22	2,85E-17
829	4876	1,22	5,30E-09
830	71380	1,22	2,03E-27
831	21120	1,22	6,44E-07
832	111887	1,22	1,17E-03
833	124341	1,22	9,24E-15
834	105970	1,21	1,51E-11
835	65029	1,21	9,49E-12
836	68412	1,21	2,53E-22
837	58772	1,21	7,16E-24
838	67605	1,21	2,49E-04
839	45624	1,21	1,89E-23
840	124002	1,20	3,41E-10
841	110813	1,20	2,07E-17
842	60578	1,20	2,11E-19
843	22771	1,20	1,18E-09
844	106471	1,20	4,35E-15
845	70375	1,20	2,58E-21
846	54366	1,20	6,42E-08
847	112613	1,20	4,15E-03
848	80240	1,19	1,42E-16
849	78357	1,19	1,74E-14
850	65925	1,19	2,54E-02
851	53569	1,19	6,93E-13
852	72800	1,19	1,32E-12
853	59073	1,19	9,19E-14
854	121597	1,18	2,49E-24
855	70383	1,18	3,81E-22
856	46902	1,18	6,47E-37
857	76218	1,18	1,85E-31
858	54972	1,18	4,53E-02
859	112230	1,18	1,00E-07
860	70639	1,18	2,29E-05
861	81296	1,18	1,52E-17
862	76324	1,18	1,69E-10
863	105349	1,17	4,99E-08
864	123616	1,17	2,12E-26
865	23083	1,17	4,11E-15
866	65969	1,17	2,09E-33
867	110276	1,17	1,89E-04
868	70548	1,16	7,88E-34
869	63692	1,16	1,50E-04
870	73881	1,16	1,44E-20
871	103812	1,16	3,35E-14
872	81457	1,16	1,05E-19
873	60482	1,16	1,58E-09
874	76652	1,15	1,18E-15
875	56117	1,15	2,45E-19
876	70316	1,15	2,43E-09
877	41761	1,15	3,82E-20
878	110767	1,15	7,53E-07
879	65085	1,15	2,05E-02
880	123499	1,15	8,59E-29
881	33482	1,15	1,61E-06
882	67133	1,14	2,47E-12
883	107386	1,14	2,59E-10
884	22637	1,14	1,30E-09
885	2837	1,14	2,18E-03
886	53091	1,14	3,14E-02
887	107244	1,14	7,96E-03
888	69489	1,14	1,66E-26
889	66111	1,14	7,53E-29
890	102737	1,14	3,03E-26
891	80200	1,14	1,21E-26
892	119607	1,13	6,97E-10
893	104072	1,13	1,21E-02
894	5016	1,13	3,84E-06
895	70098	1,13	4,50E-08
896	106029	1,13	6,93E-09
897	119876	1,13	4,03E-10
898	80881	1,13	1,65E-26
899	111681	1,13	2,52E-11
900	22402	1,12	4,30E-16

	Identifier (TRIREDRAFT)	log2fc	padj
901	75175	1,12	1,10E-02
902	106051	1,12	2,72E-16
903	123571	1,12	1,39E-24
904	31227	1,12	7,81E-17
905	74771	1,12	9,30E-14
906	104288	1,12	2,29E-06
907	59152	1,11	2,00E-04
908	65292	1,11	8,17E-09
909	121252	1,11	1,14E-29
910	55731	1,11	2,06E-14
911	67931	1,11	2,89E-11
912	52718	1,11	2,67E-15
913	78797	1,11	1,03E-24
914	65992	1,11	1,39E-10
915	123429	1,11	1,69E-12
916	108191	1,11	9,48E-06
917	120110	1,11	2,58E-24
918	66480	1,10	1,12E-27
919	55774	1,10	5,18E-10
920	105106	1,10	3,13E-22
921	82146	1,10	1,58E-13
922	69695	1,10	1,30E-04
923	107253	1,10	4,16E-04
924	111803	1,10	2,29E-07
925	59778	1,10	2,46E-18
926	71532	1,10	2,15E-04
927	63152	1,10	1,19E-16
928	79237	1,10	2,99E-11
929	78738	1,10	2,56E-18
930	81303	1,09	1,64E-21
931	5363	1,09	1,09E-05
932	81652	1,09	3,20E-22
933	53824	1,09	1,76E-02
934	60489	1,09	3,59E-02
935	109929	1,09	1,31E-12
936	68990	1,09	2,49E-02
937	122995	1,09	3,24E-09
938	109756	1,09	9,14E-08
939	58130	1,08	1,23E-19
940	2038	1,08	1,05E-02
941	65380	1,08	3,93E-12
942	109895	1,08	4,89E-20
943	52446	1,08	1,23E-16
944	81070	1,08	2,26E-18
945	56744	1,08	2,56E-16
946	103754	1,08	1,01E-13
947	122571	1,08	1,28E-14
948	57237	1,07	2,32E-07
949	107268	1,07	2,59E-14
950	3889	1,07	4,01E-17
951	75295	1,07	3,57E-12
952	25244	1,07	4,34E-17
953	82145	1,07	2,74E-16
954	75886	1,07	2,92E-14
955	111778	1,07	3,27E-08
956	62300	1,07	4,59E-05
957	123244	1,07	3,05E-27
958	69574	1,07	7,85E-16
959	110490	1,07	1,61E-02
960	74406	1,06	9,57E-22
961	42418	1,06	5,70E-07
962	67483	1,06	2,73E-09
963	28409	1,06	9,32E-03
964	79823	1,06	3,23E-19
965	81150	1,05	8,98E-08
966	54870	1,05	3,99E-25
967	104494	1,05	7,71E-03
968	79153	1,05	2,18E-15
969	70547	1,05	3,37E-10
970	123675	1,05	1,73E-05
971	62938	1,05	4,18E-04
972	122262	1,04	8,83E-14
973	105978	1,04	3,53E-05
974	123460	1,04	7,74E-15
975	3918	1,04	2,46E-15
976	102960	1,04	8,30E-08
977	123720	1,04	1,75E-15
978	62367	1,04	2,33E-13
979	76250	1,04	2,20E-15
980	54991	1,04	8,49E-17
981	69823	1,04	1,47E-15
982	102906	1,04	1,70E-03
983	123327	1,04	1,81E-10
984	78650	1,04	1,56E-10
985	105239	1,04	4,05E-16
986	62362	1,04	1,04E-17
987	22560	1,04	3,47E-12
988	105251	1,04	1,04E-07
989	42264	1,04	1,79E-04
990	120749	1,03	6,05E-05
991	59315	1,03	1,64E-03
992	4146	1,03	4,99E-04
993	75403	1,03	2,63E-17
994	108920	1,03	1,59E-16
995	74807	1,03	9,06E-18
996	65533	1,03	5,63E-16
997	122920	1,03	1,52E-14
998	67902	1,03	2,73E-16
999	37067	1,03	2,73E-12
1000	3653	1,03	5,71E-09

	Identifier (TRIREDRAFT)	log2fc	padj
1001	62535	1,03	1,42E-04
1002	108356	1,02	2,85E-09
1003	110088	1,02	1,18E-12
1004	79774	1,02	1,86E-09
1005	22464	1,02	3,39E-11
1006	79166	1,02	1,47E-20
1007	75794	1,02	2,49E-18
1008	3372	1,02	2,27E-21
1009	124157	1,02	7,70E-11
1010	78496	1,01	1,39E-18
1011	106037	1,01	4,91E-19
1012	79725	1,01	3,97E-07
1013	78042	1,01	4,57E-16
1014	70500	1,01	1,96E-02
1015	4009	1,01	1,26E-27
1016	103852	1,01	6,72E-19
1017	5863	1,01	2,20E-12
1018	69496	1,01	5,70E-04
1019	67239	1,01	8,91E-12
1020	27939	1,01	2,08E-11
1021	120654	1,00	5,46E-24
1022	112551	1,00	3,26E-07
1023	121163	1,00	7,80E-08
1024	121056	1,00	1,17E-18
